# Broadly conserved protective epitopes on the lyme disease vaccine antigen, OspA

**DOI:** 10.1371/journal.ppat.1013740

**Published:** 2026-04-21

**Authors:** Graham G. Willsey, Michael J. Rudolph, Carol Lyn Piazza, Yang Chen, Grace Freeman-Gallant, Lisa A. Cavacini, David J. Vance, Nicholas J. Mantis

**Affiliations:** 1 Division of Infectious Diseases, Wadsworth Center, New York State Department of Health, Albany, New York, United States of America; 2 New York Structural Biology Center, New York, New York, United States of America; 3 Department of Medicine, University of Massachusetts Chan Medical School, Worcester, Massachusetts, United States of America; Centre National de la Recherche Scientifique, FRANCE

## Abstract

Lyme disease, caused by the spirochete, *Borrelia burgdorferi* sensu latu (Bbsl), is a tickborne infection of increasing incidence in North America, Europe and Asia. While vaccines based on Outer surface protein A (OspA) have proven highly efficacious at blocking Bbsl tick-to-human transmission, the high degree of antigenic variability among the major OspA serotypes (ST) has made the development of a broadly cross protective vaccine difficult. Recent profiling of protective human monoclonal antibodies (mAbs) has suggested the existence of conserved epitopes situated within OspA’s central β-sheet (CBS), although direct comparisons of cross-serotype functionality has been hindered by biological differences among the major Bbsl genospecies. To address these issues, we developed a panel of isogenic *B. burgdorferi* reporter strains expressing the seven major OspA serotypes (ST1–7) and probed them with CBS-targeting mAbs to evaluate their complement-dependent borreliacidal activity. The mAbs segregated into three distinct classes: class 1 mAbs exhibited potent killing against all seven OspA serotypes, while classes 2 and 3 had restricted or no activity against two of the seven serotypes. Structural analysis of Fabs derived from each class of mAbs in complex with OspA ST1 showed that they target overlapping epitopes spanning β-strands 6–10 and involve contact with largely invariant residues. Further analysis of *B. burgdorferi* reporter strains expressing OspA variants from 17 additional Bbsl genospecies identified Lys-107 as a determinant of susceptibility for nearly all CBS mAbs. Taken together, these findings raise the prospect of structure-based design of a broadly protective monovalent Lyme disease vaccine.

## Author summary

Lyme disease, caused by the spirochete *Borrelia burgdorferi* sensu latu (Bbsl), is a tickborne infection of increasing incidence in North America, Europe and Asia. While vaccines based on Outer surface protein A (OspA) have proven highly efficacious at blocking Bbsl tick-to-human transmission, the genetic and serological heterogeneity of OspA across *Borrelia* genospecies has complicated matters. In this report, we use a collection of transmission-blocking human monoclonal antibodies to delineate a protective region (epitope) within the central core of OspA that is conserved across all major OspA serotypes. These results have important implications for engineering a broadly reactive Lyme disease vaccine.

## Introduction

The spirochete *Borrelia burgdorferi* sensu stricto (Bbss) is the primary etiologic agent of Lyme disease, the most common tick-borne infection in the United States [1]. The disease typically presents with a characteristic “bullseye” rash (erythema migrans) before potentially progressing to a disseminated infection, which may result in carditis, neuroborreliosis, and/or Lyme arthritis [2]. Although antibiotic treatment is generally effective at any stage of infection, a subset of individuals experience persistent symptoms known as post-treatment Lyme disease (PTLD) syndrome [3]. In Europe and Asia, Lyme disease is also caused by three additional genospecies within the *Borrelia burgdorferi* sensu lato (Bbsl) complex: *B. afzelii*, *B. garinii*, and *B. bavariensis*. While these four genospecies share key pathogenic traits, they are genetically and antigenically heterogeneous and exhibit distinct clinical manifestations. This heterogeneity has posed significant challenges to the development of broadly protective Lyme disease vaccines suitable for use across North America, Europe, and Asia.

The only licensed Lyme disease vaccine for human use in the United States was LYMERix, which was based on outer surface protein A (OspA) serotype 1 (ST1), the predominant OspA ST expressed by *B. burgdorferi* in North America [4,5]. OspA is a ~ 31 kDa lipoprotein abundantly expressed on the surface of *B. burgdorferi* in the midgut of unfed ticks. During feeding, OspA expression is downregulated as the spirochete migrates through the hemolymph and salivary glands. Nonetheless, transmission is effectively blocked if a tick feeds on a host with pre-existing OspA antibodies induced by vaccination [6–10]. Similarly, administration of certain mouse or human monoclonal antibodies (mAbs) targeting OspA prior to tick challenge is sufficient to protect rodents and non-human primates from *B. burgdorferi* infection [7,11,12]. While the precise mechanisms by which these antibodies block transmission are not fully understood, evidence supports a model in which they engage spirochetes within the tick midgut [10,13].

As the incidence of Lyme disease continues to rise in both the United States and Europe, there is renewed interest in developing OspA-based vaccines. To provide broad protection beyond North America, such vaccines must target not only *B. burgdorferi* (ST1), but also six additional OspA serotypes (ST2–7) associated with *B. afzelii* (ST2), *B. garinii* (ST3, ST5–7), and *B. bavariensis* (ST4) [14,15]. Research dating back over three decades has shown that OspA-based immunization typically confers strong protection against homologous serotype challenge but limited or no protection against heterologous serotypes [16–18]. These observations, combined with the identification of immunodominant, highly protective B cell epitopes localized to OspA’s C-terminal region, have driven the design and evaluation of multivalent OspA vaccines [9,19–22]. One such candidate, VLA15, which incorporates chimeric heterodimers of the C-terminal third of OspA ST1–6, is currently in Phase 3 clinical trials [23–26].

Monoclonal antibodies (mAbs) have been instrumental in identifying cross-protective epitopes on highly variable surface antigens from pathogens such as influenza virus hemagglutinin [27,28], HIV-1 envelope glycoprotein [29], and *Plasmodium* species [30], among others [31]. In the case of Lyme disease, we employed a collection of human mAbs alongside alpaca-derived single-domain antibodies (V_H_Hs) generated against OspA serotype 1 (OspA_ST1_) to derive the first detailed epitope map of OspA_ST1_ [11,12,32–34]. Three spatially distinct epitope “bins” encompassing more than half the predicted surface-exposed area of OspA were identified [32]. Bin 1 corresponds to OspA_ST1_’s central β-sheet (β-strands 8–10), Bin 2 corresponds to β-strands 11–13, and Bin 3 to β-strands 16–20 and the C-terminal α-helix. To identify possible epitopes shared across serotypes, Wang and colleagues screened the collection of human mAbs for complement-dependent borreliacidal activity against Bbsl strains expressing different OspA serotypes: *B. burgdorferi* (ST1), *B. afzelii* (ST2), and *B. bavariensis* (ST4) [11]. Monoclonal antibodies from Bin 1 (e.g., 221-7, 857-2) had potent borreliacidal activities across all three Bbsl strains, whereas mAbs targeting bins 2 and 3 (e.g., LA-2) were only active against *B. burgdorferi* (ST1), demonstrating that certain epitopes within Bin 1 are conserved across OspA serotypes. In support of this notion, we demonstrated by ELISA using recombinant OspA antigens that 857-2 reacts with all seven OspA serotypes [34]. The structural elucidation of 221-7 Fab-OspA_ST1_ co-complex not only underscored the possibility that there are epitopes within Bin 1 that are conserved across OspA but that these epitope(s) may also be broadly protective [12]. 221-7, for example, blocks tick-mediated transmission of *B. burgdorferi* (ST1) in both mouse and a non-human primate models of pre-exposure prophylaxis [11,12]. In this report we sought to better define the Bin 1 mAbs and their potential to impart complement-dependent borreliacidal activities against the seven major OspA serotypes.

## Results

Differences in *Bbsl* genospecies growth rates, intrinsic serum sensitivities, and susceptibility to commercially available complement sources have hindered the development of a standardized OspA binding and complement-dependent bactericidal assay suitable for all Bbsl serotypes and in silico types (ISTs) [14,15,23,25,35]. To resolve this issue, we constructed a panel of isogenic *B. burgdorferi* reporter strains expressing each of the seven major OspA serotypes (OspA_ST1–7_). This was accomplished by modifying an existing IPTG-inducible *mScarlet-I* viability reporter plasmid (pGW189) to encode different *ospA* alleles under the control of the native *ospAB* promoter from *B. burgdorferi* B31 [34]. Reporter plasmids encoding *ospA* alleles from *B. burgdorferi* B31 (ST1), *B. afzelii* PKo (ST2), *B. garinii* PBr (ST3), *B. bavariensis* PBi (ST4), *B. garinii* PHei (ST5), *B. garinii* DK29 (ST6), and *B. garinii* T25 (ST7) were introduced into *B. burgdorferi* strain HB19-R1, a high passage North American isolate that lacks *ospA* due to the loss of lp54 (**S1** **and** **S2 Tables**) [36,37].

**Fig 1:**
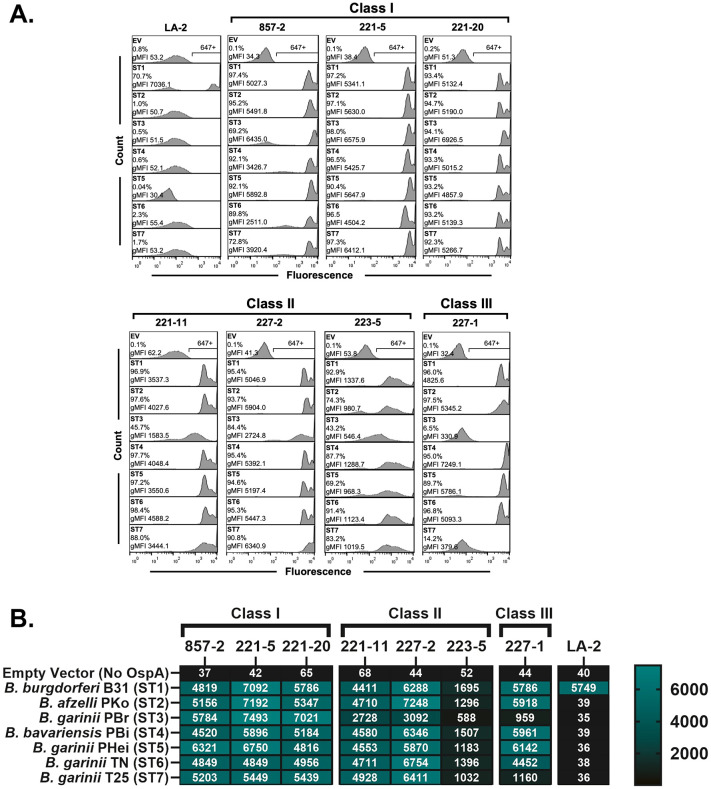
Surface binding profiles of anti-OspA_ST1_ Bin 1 mAbs to *B. burgdorferi* OspA ST1-7. **(A)** Representative flow cytometric histograms of live *B. burgdorferi* HB19-R1 reporter strains expressing OspA ST 1-7, each probed with 10 µg/ml of mAbs indicated in bold text above each column, followed by Alexa 647-labeled goat anti-human IgG secondary antibody. HB19-R1 harboring the empty vector (EV) was used as a negative control. The horizontal bracket represents the region of events positive for fluorescence labeling (647+) on the subsequent plots. The percent (%) of events positive for Alexa 647 fluorescence labeling and the geometric mean fluorescence intensity (gMFI) are shown in the top left corner of each box. Compared to the empty vector (EV) controls, the gMFI values were significantly greater for binding of LA-2 to ST1 (p < 0.0001); 857-2 to ST1-7 (p < 0.0001); 221-5 to ST1-7 (p < 0.0001); 221-20 to ST1-7 (p < 0.0001); 221-11 to ST1 (p < 0.001), ST2, 4-7 (p < 0.0001), and ST3 (p < 0.05); 227-2 to ST1-2, 4-7 (p < 0.0001) and ST3 (p < 0.05); 227-1 to ST1-2, 4-6 (p ≤ 0.0001). N = 3. Statistical comparisons were performed using two-way ANOVA with Dunnet’s multiple-comparison test. Panels and plots were assembled using FlowJo. **(B)** Heat map summarizing the average gMFIs (with scale shown on right) of *B. burgdorferi* HB19-R1 reporter strains probed with each of the different mAbs depicted in Panel **A.** The results per box are the average of three biological replicates.

To validate OspA expression on the surface of the reporter strains, we assessed the reactivity of two mAbs, 857-2 and LA-2, against the panel and compared their profiles to corresponding native Bbsl strains (Fig 1 and S1 Fig). 857-2 was reactive with all seven OspA serotypes expressed in the HB19-R1 background, with median fluorescence intensity (MFI) values ranging from 2500-6400. In contrast, LA-2 was only reactive with OspA_ST1_ (>70% reactivity; MFI > 7,000). Neither 857-2 nor LA-2 reacted with HB19-R1 carrying the empty vector (MFI < 55). The same OspA reactivity profiles were observed when 857-2 and LA-2 were used to probe the native Bbsl strains (**S1 Fig**): 857-2 recognized all seven OspA serotypes, while LA-2 only recognized OspA_ST1_. These results not only demonstrate that the HB19-R1 reporter strains are reliable proxies for assessing antibody binding to different OspA serotypes but also confirm that 857-2 can recognize the seven major OspA serotypes [34].

The HB19-R1 reporter strains were then probed with the additional mAbs targeting OspA_ST1_’s central β-sheet (so-called Bin 1 mAbs) to assess breadth of OspA serotype recognition (**Table 1**). Interestingly, the mAbs fell into three distinct classes (**Fig 1**). Class I mAbs (857-2, 221-5, 221-20) reacted strongly with OspA_ST1–7_ with MFIs > 4000, while the Class II mAbs (221-11, 227-2, 223-5) had diminished recognition of OspA_ST3_. A single mAb, 227-1, was defined as Class III because of significantly reduced binding to OspA_ST3_ and OspA_ST7_. To determine if these class designations are a function of mAb binding to the different OspA serotypes and not an artefact of expressing heterologous OspA types on the surface of HB19-R1, we evaluated mAb binding to recombinant OspA_ST1–7_ by microsphere immunoassays (MIA). The mAb binding profiles to recombinant OspA_ST1–7_ mirrored exactly those observed with the HB19-R1 reporter strains (**S2 Fig**). These results indicate that a subset of Bin 1 mAbs, namely those designated as Class I (857-2, 221-5 and 221-20), recognize epitopes that are conserved across the major OspA serotypes.

**Table 1:** Characteristics of OspA mAbs used in this study.

mAb	bin^*a*^	class^*b*^	K_D_^*c*^	iGL^*d*^ V_H_	iGL V_L_	HCDR3	PDB
221-7^*e*^	1	(2)	0.66	HV5–51	KV1–13	ARGILRYFDWFLDY	7JWG
857-2	1	1	0.30	HV5–51	KV1–13	ARHITTHTYRGFFDF	8TUZ
221-5	1	1	0.52	HV5–51	KV3–11	ARHLGIDYFFGMDV	8TV3
221-20	1	1	0.37	HV5–51	KV3–11	ARHLGIDYFFGLDV	n.a.
221-11	1	2	0.35	HV5–51	KV1–13	VRGIGRYSNWFLDY	8TVD
227-2	1	2	0.48	HV5–51	KV1–13	VRSISRSGPGYILSWFFDY	n.a.
223-5	1	2	0.17	HV5–51	KV1–13	ARYVTVGAFDF	n.a.
227-1	1	3	1.7	HV5–51	KV1–13	TRSISRSGPGFSLSWFFDY	8TVJ
LA-2	3	n.a.	64	n.a.	n.a.	n.a.	1FJ1

^*a*^, from Haque et al 2022; ^*b*^, n.a. not applicable, n.d. not determined; ^*c*^, nM; ^*d*^, inferred germline sequence; ^*e*^, from Schiller et al, 2022; 221-7 inferred to be in class 2, based on S2 Fig;

### Cross-serotype borreliacidal activity associated with Bin 1 mAbs

**Fig 2:**
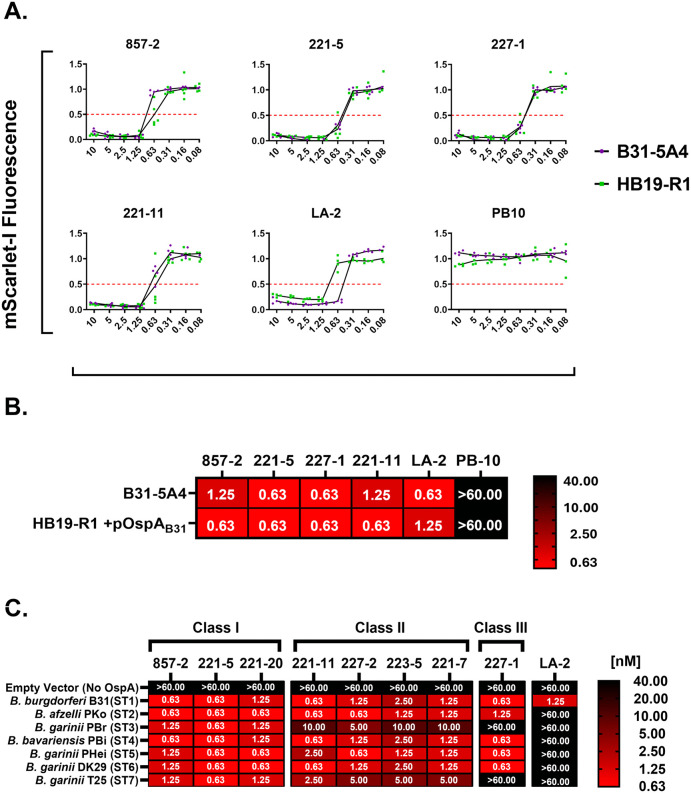
OspA Bin1 mAbs promote complement-dependent killing of recombinant *B. burgdorferi* strains expressing OspA ST1-7. **(A)** Borreliacidal mAb titration curves and **(B)** heat map comparing the susceptibilities of an infectious *B. burgdorferi* B31-5A4 *mScarlet-I* viability reporter strain and an HB19-R1 reporter strain expressing the same OspA_ST1_ (B31) allele to complement-dependent killing mediated by OspA_ST1_ mAbs or an isotype IgG1 (PB10) control. Experiments were performed under conditions optimized for each strain as described within the Materials and Methods. Ec_50_ values represent the mean minimum concentration (nM) of mAb resulting in >50% reduction in MFI relative to controls, as determined from 3-5 independent experiments per strain. Statistical differences in mAb susceptibility were assessed via two-way ANOVA followed by Tukey’s multiple comparison test. No significant differences were observed. **(C**) Heat map depicting the mean Ec_50_ (nM) of Bin 1 mAbs against recombinant *B. burgdorferi* strains expressing OspA ST 1-7. Complement-dependent borreliacidal assays were performed with anti-OspA_ST1_ Bin 1 mAbs and a panel of *B. burgdorferi* HB19-R1 (*ospA* negative) strains harboring an IPTG-inducible *mScarlet-I* viability reporter plasmid and expressing *ospA* ST1-7 under conditions described in the Materials and Methods. Controls included an HB19-R1 strain carrying the IPTG-inducible viability reporter plasmid alone (empty vector, *ospA* negative) and a mAb with borreliacidal activity restricted to OspA_ST1_ (LA-2). EC_50_ values shown represent the mean lowest concentration of antibody (nM) resulting in >50% reduction in MFI relative to controls. Reporter strains that exhibited resistance to complement-mediated killing by mAbs at 10 nM were later retested at a single higher dose (66.6 nM). The data shown encompasses 3-5 independent experiments per strain with data normalized as described within the Materials and Methods section. Corresponding antibody titration curves and statistical comparisons are shown in **S3 Fig**.

We next wished to determine the functional capacity of the Bin 1 mAbs to promote complement-dependent borreliacidal activity across the different OspA serotypes. To validate the HB19-R1 OspA reporter strains for this purpose, we compared complement-dependent killing of an infectious *B. burgdorferi* B31-5A4-based reporter strain [34] to the HB19-R1 OspA_ST1_ derivative. The assays were performed under conditions optimized for each strain, due to slight differences in growth kinetics and intrinsic complement sensitivities. Cultures were mixed with serial mAb dilutions (0.08-10 nM) in medium containing 10% (B31-5A4) or 2.5% (HB19-R1) guinea pig complement. The following day, IPTG was added to the cultures to induce *mScarlet-I* expression and MFI was measured 24 h (HB19-R1) or 48 h (B31-5A4) later, as described in the Materials and Methods. Under these conditions, the five Bin 1 mAbs had EC₅₀ values ranging from 0.63 to 1.25 nM against both the B31-5A4 and HB19-R1 OspA_ST1_ reporter, while an IgG isotype control (PB10) had no effect on spirochete viability (**Fig 2A and 2B**). Moreover, the EC_50_ values for 857-2 and 221-7 obtained using the HB19-R1 OspA_ST1_ strain closely match those reported for *B. burgdorferi* B31 derived using a completely different viability assay [11]. Collectively, these results demonstrate that OspA expression is comparable in both *B. burgdorferi* genetic backgrounds. Thus, the HB19-R1 reporter strains served as reliable readouts of OspA-mediated complement-dependent borreliacidal activity.

We therefore used the panel of HB19-R1 OspA reporters to assess the ability of the full collection Bin 1 mAbs (8 in total) to confer cross-serotype, complement-dependent borreliacidal activity (**Fig 2C**). The results reflected the OspA binding profiles in that **Class I** mAbs (857-2, 221-20, and 221-5) exhibited potent borreliacidal activity against all seven OspA serotypes, with EC₅₀ values ranging from 0.63 to 1.25 nM. Providing further validation of our HB19-R1 reporter system, 857-2’s EC_50_ against the ST4 reporter strain is consistent with the previously reported value for *B. bavariensis* strain (PBi), from which the OspA_ST4_ sequence was derived [11]. **Class II** mAbs (221-11, 223-5, 227-2, and 221-7) had reduced potency against OspA ST3 and ST7 (EC_50_ 2.5-10 nM), while 227-1 (**Class III**) was completely devoid of ST3 and ST7 killing.

### Structural basis of OspA_ST1_ recognition by Bin 1 mAbs

**Fig 3:**
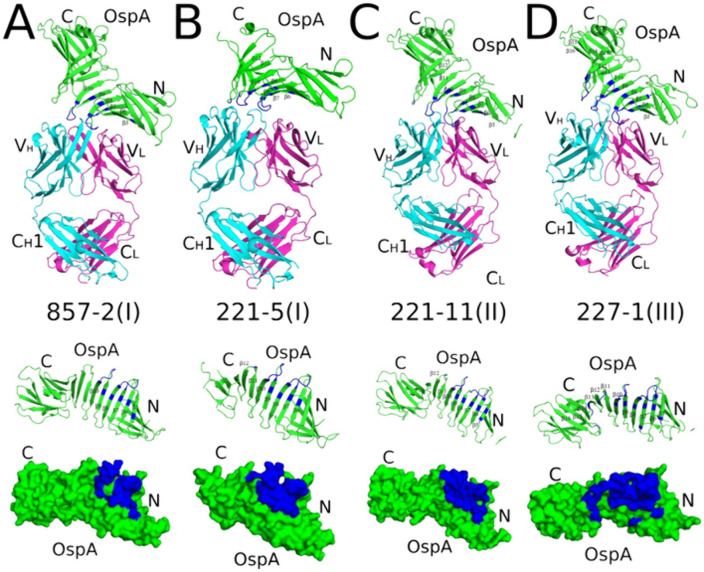
Crystral structures of Fab-OspA complexes reveal conserved epitopes on OspA_ST1_. (**top row**) Ribbon diagrams of OspA_ST1_ (green) in complex with Fabs from (A) 857-2 (class **I)**, (B) 221-5 (class **I)**, (C) 221-11 (class **II)**, and (D) 227-1 (class **III)**. Fab heavy chains (V_H_ and C_H_1) are colored cyan; light chains (V_L_ and C_L_) are colored magenta. The OspA N- and C-termini are labelled accordingly, along with selected strand numbers. (**middle row**) The four OspA (green) ribbon representations aligned in the same orientation with respective Fab contract points colored in blue. (**bottom row**) The ribbon and surface representations of OspA highlight the Fab-interacting residues, shown in blue. The N and C-termini of OspA are labelled N and C, respectively. PDB ID for each structure are provided in Table 2.

To elucidate the epitopes recognized by the three different classes of Bin 1 mAbs, we solved the X-ray crystal structures of Fabs 857-2 (**Class I**), 221-5 (**Class I**), 221-11 (**Class II**), and 227-1 (**Class III**) in 1:1 stoichiometric complexes with OspA_ST1_ at resolutions ranging from 2.2 Å to 3.2 Å (**Tables 2 and** **S3** **and Fig 3**). Attempts to solve the X-ray crystal structure of 221-20 Fabs in complex with OspA were unsuccessful. After molecular replacement, the resulting phase information was used to calculate electron density maps employed to manually insert the corresponding residues into each model while manually building additional regions within each Fab-OspA model. Refinement of the four Fab-OspA structures revealed molecular models that possessed excellent geometry and that were consistent with the crystallographic data (**S4 Table**).

**Table 2:** Summary of OspA-Fab interactions.

mAb	class ^*a*^	BSA^*b*^	H-b^*c*^	SB^*d*^	SC^*e*^	PDB ID^*f*^
857-2	1	1397	7	3	0.61	8TUZ
221-5	1	1423	8	5	0.74	8TV3
221-11	2	1348	6	3	0.52	8TVD
227-1	3	1704	5	3	0.47	8TVJ

^*a*^, Buried surface area (Å^2^); ^*b*^, hydrogen bonds; ^*c*^, salt bridges; ^*d*^, shape complementarity; ^*e*^, Protein Data Bank ID (https://www.rcsb.org/).

In each of the four complexes, the Fabs assumed a canonical structure with two heavy chain immunoglobulin domains (V_H_, C_H_1) and two light immunoglobulin domains (V_L_, C_L_) each containing 7–9 β-strands arranged in two β-sheets that folded into a two-layer sandwich with all six CDRs (L1-3; H1-3) on one face of each molecule. OspA was comprised of one antiparallel β-sheet with 21 β-strands (referred to as β-strands 1–21) connecting globular N- and C-terminal domains with a single α-helix (referred to as α-helix A) at the C-terminus (**Fig 3**). All 21 β-strands were present in their respective electron density maps of OspA complexed with Fabs 227-1 and 857-2. In the OspA-221-11 complex β-strands 1 and 2 were not visible in the electron density maps, while β-strands 17–21 were absent in the electron density maps of the OspA-221-5 complex, likely because of greater flexibility within these regions of OspA when bound to the particular Fabs. The OspA molecules from each of the four complexes were structurally similar to OspA alone (PDB ID: 2G8C), as evidenced by Root Mean Square Deviation (RMSD) of 0.8 Å to 2.4 Å upon superposition (**S3 Fig**). Thus, the Fabs did not induce any major conformational changes within OspA.

The OspA-Fab structures revealed that 857-2 (**Class I**), 221-5 **(Class I**), 221-11 (**Class II**), and 227-1 (**Class III**) recognize overlapping but distinct epitopes on OspA with similar modes of engagement (**Fig 3**). The four Fabs contact OspA β-strands 6–10 and loop regions between β-strands 7–8 (loop 7–8) and 9–10 (loop 9–10), including residues 87, 100, 102, 105, 106, 107–109, 112, 124, 126, 128–132 (Fig 3). All but 857-2 Fab contacts OspA loop 11–12.

**Fig 4:**
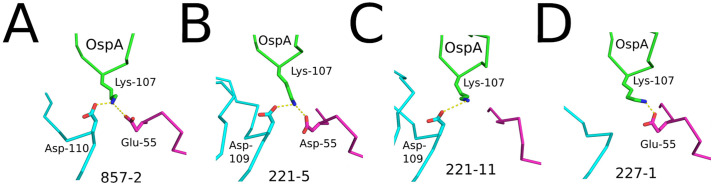
Key interactions between each Fab and OspA. Close-up of notable salt bridges between OspA Lys-107 (green) and Fab residues are shown as yellow dashed lines. Fab heavy chains (V_H_/C_H_1) are colored cyan, and light chains (V_L_/C_L_) are colored magenta. **(A)** Fab 857-2 engages Lys-107 through Glu-55 and Asp-110. **(B)** Fab 221-5 interacts with Lys-107 via Asp-55 and Asp-109. **(C)** Fab 221-11 contacts Lys-107 through Asp-109. **(D)** Fab 227-1 engages Lys-107 through Glu-55. All side chains are drawn as sticks and color coordinated to the main chain color with nitrogen atoms blue and oxygen atoms red.

Fab 857-2 (**Class I;** K_D_ 0.30 nM) and 221-5 (**class 1;** K_D_ 0.52 nM) buried 1,397 Å² and 1,423 Å^2^, respectively, involving CDRs H1, H2, H3, and L2. 857-2 formed seven hydrogen bonds and three salt bridges with OspA, while 221-5 forms 8 hydrogen bonds and five salt bridges with OspA (**Table 2**). The Fabs each form two salt bridges with OspA residue Lys-107; one via CDR-L2 residue Asp/Glu-55 and the other with CDR-H3 residue Asp-109/110 (**Fig 4A-4B**). Fab 221-5 makes contacts with Ser-152 in loop 11–12 via framework region (FR) residue Arg-59 but is too distant (4.2 Å) to form an H-bond. 857-2 does not interact with loop 11–12 at all, as Arg-59 is > 9 Å from OspA Ser-152.

Fab 221-11 (**Class II;** K_D_ 0.35 nM) engages with OspA via CDRs H1, H2, H3, and L2 and has a total buried surface area of 1,348 Å^2^ (**Table 2**). It forms 9 polar contacts with OspA, including a hydrogen bond between CDR-H1 Tyr-32 and OspA Asp-105. Like 857-2 and 221-5, 221-11 also forms a salt bridge between CDR-H3 residue Asp-109 and OspA residue Lys-107 **(Fig 4C)**. However, unlike the other two mAbs, 221-11 light chain FR Glu-55 does not form a second H-bond with Lys-107 (**Fig 4C**).

Fab 227-1 (**Class III;** K_D_ 1.7 nM) stands out from the other three Fabs in that it forms the most extensive interface with OspA, burying a total of 1,704 Å² with contributions from five of the six CDRs (H1-3, L1-2). Fab 227-1 establishes five hydrogen bonds and three salt bridges with OspA, including with Lys-107 (**Fig 4D**). FR residue Arg-59 forms a hydrogen bond with Ser-152 of OspA, as will be discussed in detail below. 227-1 makes additional contacts with OspA not observed in the other three Fabs, including with loop 11–12 (residues 153–154), β-strand 13, loop 13–14 (residues 171–173), and loop 15–16 (residue 193). These additional interfaces result in 239 Å² of total buried surface area. This larger binding surface likely comes at a cost, considering that 227-1 (**Class III**) has a restricted OspA serotype profile (i.e., fails to bind OspA_ST3_ and OspA_ST7_) relative to 857-2 and 221-5, which bind the seven major OspA types.

### Structural basis of cross serotype restriction of Bin 1 mAbs

**Fig 5:**
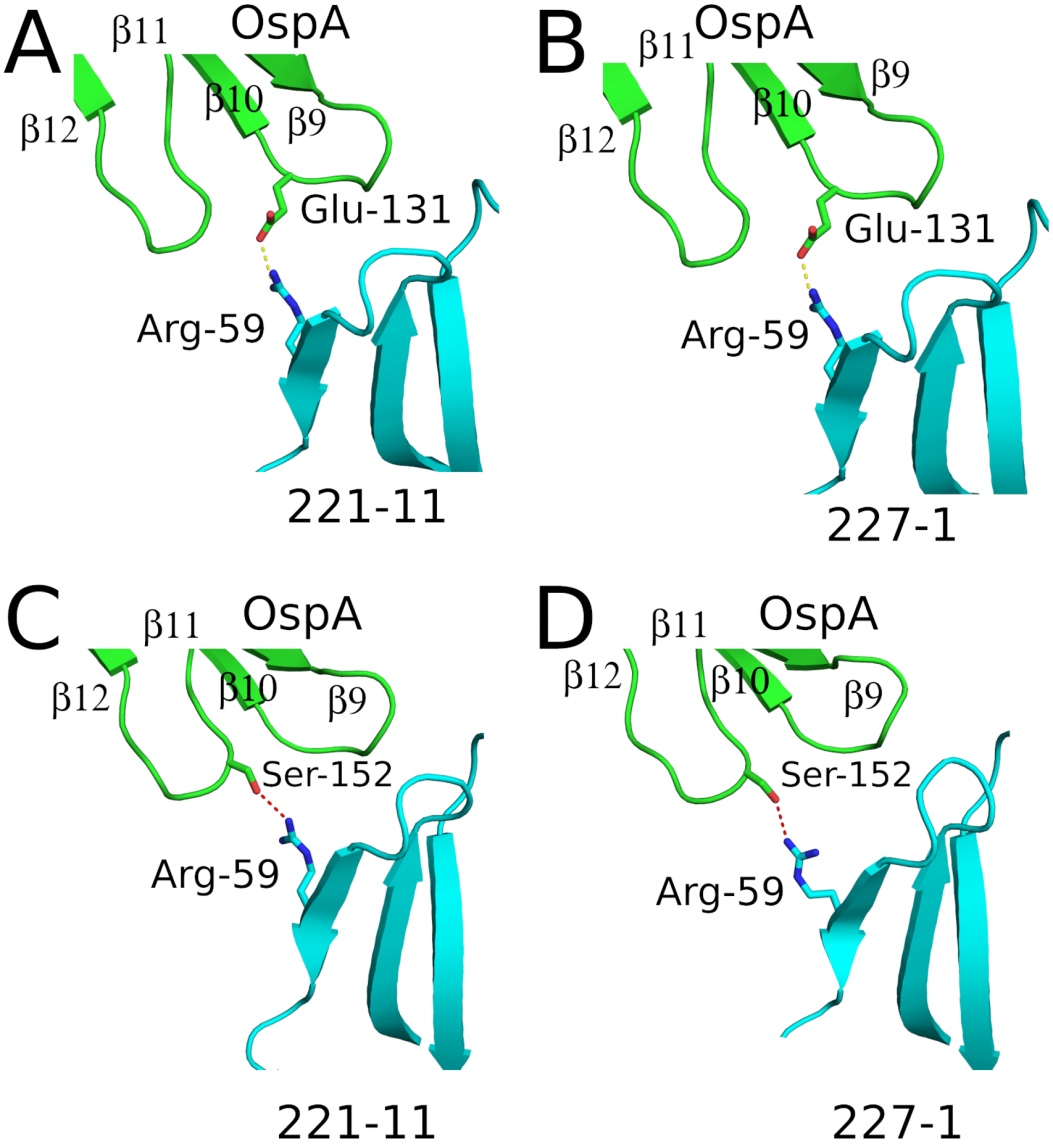
Influence of OspA primary sequence deviations on antibody function. **(A)** Structural basis for Fabs 221-11 and 227-1 cross-reactivity loss in OspA serotypes 3 and 7. Distinct hydrogen bond networks between Fab Arg-59 (cyan) and OspA residues (green) contribute to serotype specificity. **(A)** In Fab 221-11, Arg-59 forms a salt bridge with OspA Glu-131. **(B)** The same Glu-131 interaction is observed in Fab 227-1. **(C)** In 221-11, Arg-59 hydrogen bonds with OspA Ser-152. **(D)** Similarly, Fab 227-1 also engages Ser-152 through Arg-59. The primary sequence difference distinguishing serotypes (STs) 1, 2, 4, 5, and 6 from STs 3 and 7 involves a Glu-Lys substitution at position 131 and a Ser-Asn at position 152. These two primary sequence differences conceivably disrupt the Arg-59–Glu-131 salt bridge and the Arg-59 hydrogen bond with Ser-152, consequently reducing the cross-reactivity of Fabs 221-11 and 227-1 with ST3 and ST7. All side chains are drawn as sticks and color coordinated to the main chain color with nitrogen atoms blue and oxygen atoms red. Salt bridges are represented as yellow dashes and hydrogen bonds are red dashes.

Based on the structural analysis of the OspA-Fab interactions, we postulate that two distinct molecular interactions account for the reduced binding of 221-11 (**Class II**) and 227-1 (**Class III**) to OspA ST3 and ST7. The first interaction involves the Glu_ST1_ > Lys_ST3/7_ polymorphism at position 131 (**Fig 5A and 5B**). For example, 227-1 V_H_ FR3 residue Arg-59 forms a salt bridge with Glu-131 in OspA_ST1_, which would be disrupted by the substitution of a Lys at that position. Moreover, a mutation to Lys at position 131 would introduce electrostatic repulsion with Lys-129, which is located ~5 Å away. As a result, the salt bridge between OspA Lys-129 and 227-1 V_H_ residue Asp-55 would be perturbed. Thus, a single Glu_131_Lys polymorphism would result in the loss of two salt bridges in the case of 221-11 and 227-1. Second, the Ser_ST1_ > Asn_ST3/7_ polymorphism at position 152 may restrict 221-11 (**Class II**) and 227-1 (**Class III**) recognition of OspA ST3 and 7. 221-11 and 227-1 each form H-bonds with OspA_ST1_ residue Ser-152 via V_H_ residue Arg-59 (**Fig 5C and 5D**). The Asn polymorphism at this position would perturb a corresponding interaction in OspA_ST3_ and OspA_ST7_.

### OspA recognition is driven by V_H_ and V_L_ germline-encoded residues

All eight of the Bin 1 mAbs, including the four whose structures were solved in complex with OspA (857-2, 221-5, 221-11, 227-1) as well as the previously reported 221-7 (PDB 7JWG), are inferred to utilize the same HV5–51 germline lineage (**Table 1** and **S6 Fig**). In the case of 221-5 (**Class I**), the V_H_ is unmutated from the HV5–51 germline, while the V_H_ elements of the other four mAbs with solved co-complexes have only nominal mutations (4–6 amino acids) that are not associated with direct OspA interactions. Additionally, the H-CDR3 residue involved in the well-conserved salt bridge with Lys-107, namely Asp109 (or Asp110 or 114, depending on CDR3 length), is encoded by the J_H_ gene segment in five of the six human germline J genes (J_H_1 being the exception) (**Table 1**). Similarly, in six of the mAbs, the V_L_ is derived from the KV1–13 with germline residues involved in critical contacts, including Glu-55 that forms a salt bridge with OspA Lys-107 (**Fig 4**). In the case of 221-5 and 221-20 (**Class I**), which are derived from KV3–11, a single mutation in codon 55 (C > A, resulting in amino acid mutation Ala > Asp) would be sufficient to form a salt bridge with OspA Lys-107. Therefore, BCR combinations with HV5–51 and either KV1–13 or KV3–11 would be expected to have broad affinity for OspA serotypes.

### Borreliacidal epitopes are conserved across OspA in diverse *Bbsl* genospecies

With a detailed structural understanding of the interactions of Bin 1 mAbs with OspA_ST1_, we sought to examine epitope conservation across the *Bbsl* genospecies complex. To accomplish this, we surveyed OspA protein sequence diversity throughout the *Borrelia* genus to identify novel OspA types beyond the eight STs and nine ISTs that have been reported [38]. To this end, we constructed an unrooted phylogenetic tree consisting of 135 OspA protein sequences collected from all 23 recognized *Bbsl* genospecies [39]. To facilitate the identification of clades corresponding to known ST/ISTs, we included 86 OspA reference sequences obtained from the PubMLST OspA IST collection assembled by Lee [38].

**Fig 6:**
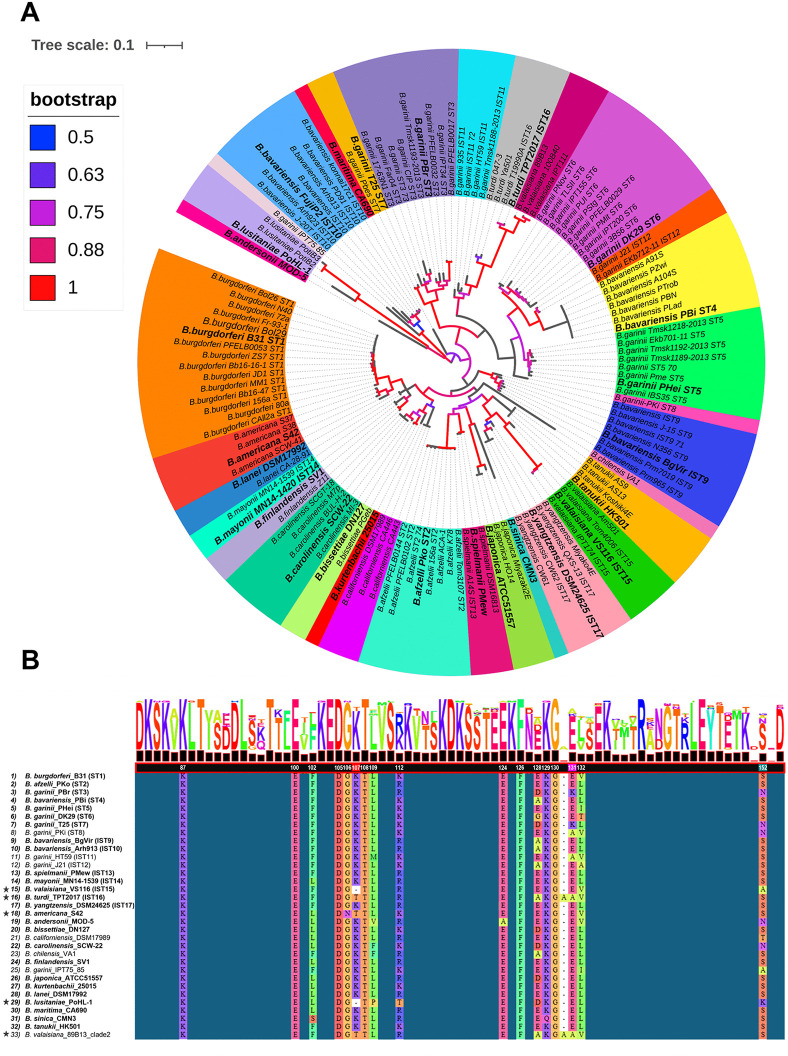
Phylogenic analysis of OspA within the *Borrelia burgdorferi sensu lato* (Bbsl) genospecies complex. **(A)** Unrooted phylogenetic tree comprised of 135 OspA protein sequences from 23 *Bbsl* genospecies. Phylogenetic analysis identified 33 distinct OspA clades corresponding to serotypes (ST) 1-8, *in silico* types (IST) 9-17, and 16 other unclassified OspA IST’s. Clades representing each OspA variant/type are distinguished by color. Enlarged and bolded strain names denote OspA alleles selected for HB19-R1 viability reporter strain construction. Bootstrap values representing approximately maximum likelihood analysis are distinguished by color: > 0.50 = green, > 0.75 = blue, 1.00 = red. Nodes with bootstrap values under 0.50 are not depicted in the figure (gray) **(B)** Simplified multiple sequence alignment of the Bin1 epitope region of 33 unique OspA types. Representative sequences from each OspA clade were aligned using OspA from *B. burgdorferi* strain B31 as a reference. Bolded strain names indicate OspA variants expressed by HB19-R1 viability reporter strains. Amino acid residues numbered in white denote conservation within at least two Bin 1 mAb epitopes with solved Fab-OspA crystal structures (857-2, 221-5, 221-11, 221-7, and 227-1). Numbered amino acid residues with colored background form critical interactions with residues within the paratopes of Bin 1 mAbs with solved crystal structures (Lys-107, Glu-31, Ser-152).

The resulting phylogenetic tree revealed 33 distinct sequence clusters corresponding to OspA ST 1–8, IST 9–17, and 16 other sequence groups representing previously untyped OspA variants **(Fig 6A)**. The 86 OspA reference sequences grouped into discrete clades consistent with their previously designated ST or IST designations [38]. The one exception was *B. valaisiana*, which clustered into two distinct clades: four sequences clustering as IST15, and three other sequences sharing close homology with IST16, principally associated with *B. turdi* (**Fig 6A**). We also identified a single *B. garinii* OspA sequence (isolate IPT75) that grouped independently of the eight recognized OspA types found within this genospecies. The 14 other OspA clades identified corresponded to untyped *B. burgdorferi* sl genospecies, including *B. americana, B. andersonii, B. bissettiae, B. californiensis, B. carolinensis, B. chilensis, B. finlandensis, B. japonica, B. kurtenbachii, B. lanei, B. lusitaniae, B. maritima, B. sinica,* and *B. tanukii* **(Fig 6A)***.*

### Genetically diverse OspA types are susceptible to Bin 1 mAbs

To assess epitope conservation across the *Bbsl* genospecies complex, we generated a multiple sequence alignment (MSA) of representative OspA sequences from each of the 33 clades identified in our phylogenetic analysis. Using OspA ST1 as the reference, the alignment revealed broad conservation of the loop regions between β-strands 7–8 and 9–10, including residues associated with 857-2 and other Bin1 mAb epitopes (**Fig 6B and** **S7 Fig**).

To experimentally examine conservation of 857-2’s and other Bin 1 epitopes beyond OspA ST 1–7, we generated 19 additional HB19-R1 viability reporter strains expressing OspA variants from two additional *B. bavarienisis* types (IST9, IST10) and 17 other *Bb*sl genospecies. Many of the OspA types selected are occasionally associated with Lyme disease in humans such as *B. bavariensis* IST9 and IST10, *B. spielmanii* (IST13), *B. mayonii* (IST14), *B. americana*, *B. bissettiae*, and *B. lusitaniae* or exhibit limited or uncertain disease potential in humans like *B. andersonii,* and *B. yangtzensis* [39–42]. We also generated HB19-R1 viability reporter strains expressing OspA types from non-pathogenic Bbsl genospecies to fully capture Bbsl complex-wide sequence diversity and explore the impact of polymorphisms predicted to disrupt Bin 1 mAb binding. These included *B. valaisiana* (IST15), *B. turdi* (IST16), *B. sinica*, *B. finlandensis*, *B. japonica*, *B. lanei*, *B. carolinensis*, *B. kurtenbachii*, *B. maritima*, and *B. tanuki* [39,41,43]. The source strains for each OspA type or IST are indicated in bold within the phylogenetic tree **(Fig 6A)** and corresponding multiple sequence alignment **(Fig 6B** **and** **S7 Fig****).**

**Fig 7:**
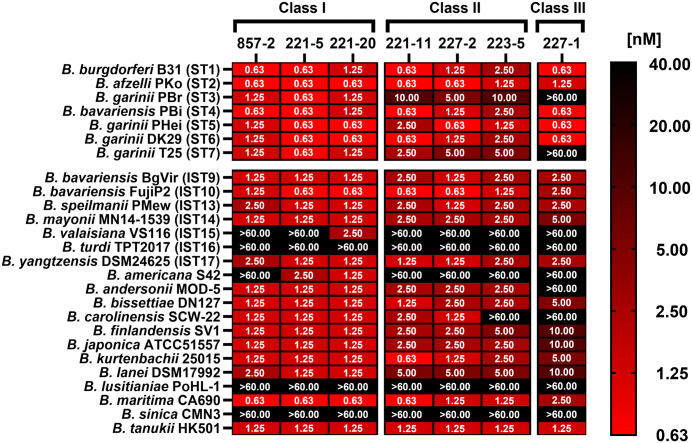
Genetically diverse OspA types are susceptible to complement-mediated killing by anti-OspA_ST1_ Bin 1 mAbs. Complement-dependent bactericidal assays were performed as described in Materials and Methods using anti-OspA Bin 1 mAbs and a panel of *B. burgdorferi* HB19-R1 *mScarlet-I* viability reporter strains expressing 19 OspA *in silico* types (ISTs). The heat map summarizes mean EC_50_ values for complement-mediated killing by Bin 1 mAbs from Classes 1-3. EC_50_ values represent the lowest mean antibody concentration (nM) producing >50% reduction in *mScarlet-I* fluorescence relative to untreated controls and were derived from ≥3 independent experiments. Susceptibility profiles of reporter strains expressing OspA ST1-7 are included for comparison. Strains that exhibited resistance to mAbs at 10 nM were retested at 66.6 nM. Corresponding antibody titration curves and statistical comparisons between the HB19-R1 OspA ST1 reporter strain and strains expressing OspA ST2-7 are shown in **S8 Fig**.

The Bin 1 mAbs were then evaluated for complement-dependent borreliacidal activity against the 19 additional OspA reporter strains described above. **Class I** mAbs (221-20, 221-5, 857-2) were the most broadly functional, as exemplified by 221-20, which exhibited potent borreliacidal activity against 23 of the 26 OspA reporter strains within our collection [EC_50_ 1.25-2.50 nM] (**Fig 7**). The three resistant strains (EC_50_ > 60 nM) were *B. turdi* (IST16), *B. lusitaniae,* and *B. sinica*. 221-5 and 857-2 susceptibility profiles were similar to 221-20, with the addition of both having lessened activity against *B. valaisiana* (IST15), and 857-2 also being ineffective against *B. americana* (**Fig 7**). The **Class II** mAbs (221-11, 227-2 and 223-5) had borreliacidal activity profiles similar to 857-2, albeit with reduced potencies (**Fig 7**). Interestingly, 223-5 was distinct among this class for its inability to kill *B. carolinensis*. Finally, 227-1 (**Class III**) had borreliacidal activity against just 17 of the 26 OspA reporter strains and reduced efficacy (Ec_50_ 5–10 nM) against OspA variants from *B. mayonii* (IST14), *B. finlandensis, B. japonica,* and *B. lanei* (**Fig 7**). These results demonstrate that one or more borreliacidal epitopes are conserved across virtually all the major variants within the *Bbsl* genospecies complex.

### Lys-107 polymorphisms impart resistance to virtually all Bin 1 mAbs

As noted above, HB19-R1 strains expressing OspA from *B. turdi* (IST16) and *B. lusitaniae* PoHL-1 were resistant to all the Bin 1 mAbs tested, while *B. americana* and *B. valaisiana* (IST15) were resistant to a subset of **Class I** mAbs and all the **Class II** and **Class III** mAbs (**Fig 7**). Examination of the OspA MSA (**Fig 6B**) suggested that polymorphisms at OspA_ST1_ Lys-107 may account for all or part of the observed resistance. In the case of *B. turdi* (IST16), for example, there is a Thr residue rather than a Lys at position 107, while OspA from *B. lusitaniae* has a deletion at the equivalent position (**Fig 6B**). Polymorphisms at Lys-107 would preclude 857-2 and other mAbs from forming critical salt bridges with OspA (**Fig 4**).

**Fig 8:**
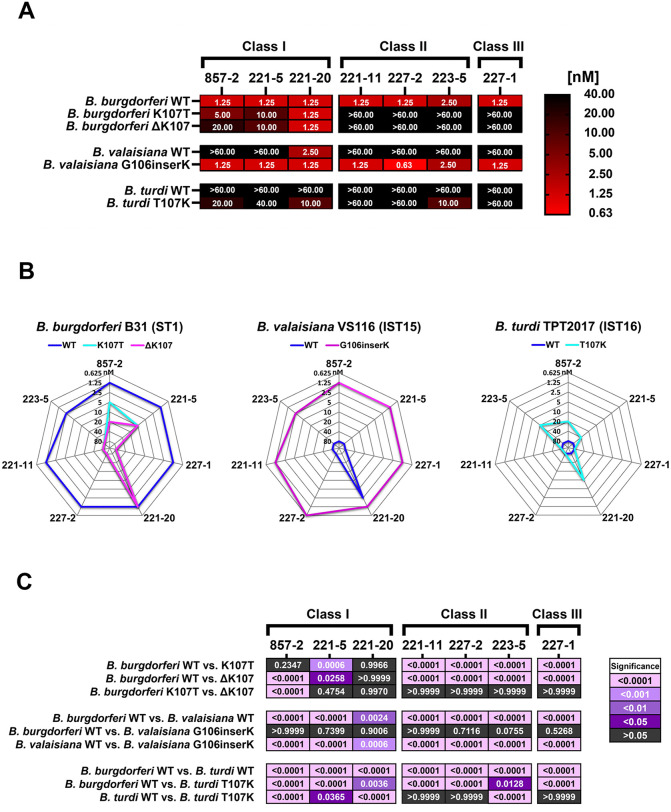
Lys-107 polymorphisms are a determinant of susceptibility to nearly all Bin 1 mAbs Complement-dependent killing assays were performed as described in the Materials and Methods with anti-OspA Bin1 mAbs and *B. burgdorferi* HB19-R1 viability reporter strains expressing “WT” OspA variants from *B. burgdorferi* B31 (ST1), *B. valaisiana* VS116 (IST15), and *B. turdi* TPT2017 (IST16), or mutated derivatives containing deletions, insertions, or substitutions at residue 107. Heat maps **(A)** and radar plots **(B)** depicting the Ec_50_ profiles of WT and muta**t**ed OspA variants. Ec_50_ values denote the lowest antibody concentration (nM) capable of reducing mScarlet-I fluorescence by >50% relative to untreated controls and represent the mean of > 3 independent experiments. Reporter strains exhibiting resistance to killing by mAbs at 20 nM were re-examined at 40 nM and 66.6 nM doses. **(C)** Heat maps graphically depicting significant differences in mAb susceptibility between HB19-R1 reporter strains expressing WT and mutated OspA variants as determined via two-way ANOVA followed by Tukey’s multiple comparisons test. Adjusted P-values reflecting significant differences between strains are shown in shades of purple or pink. Antibody titration curves summarizing borreliacidal activity against each strain are provided in **S9 Fig**.

To investigate the importance of Lys-107 in the context of OspA recognition by the Bin 1 mAbs, we generated HB19-R1 viability reporter strains expressing OspA_ST1_ variants harboring a K107T substitution or Lys-107 deletion (ΔK107). Complment-dependent killing assays revealed that the OspA_ST1_ K107T and ΔK107 reporter strains were completely resistant to **Class II** (227-2, 223-5, 221-11) and **Class III** mAbs (227-1), even at the highest antibody concentrations tested (66.6 nM) (**Fig 8**). Similarly, both modifications were detrimental to **Class I** mAbs 857-2 and 221-5’s borreliacidal activity, as evidenced by 16- fold increases in EC_50_ values (**Fig 8**). For 857-2, the OspA_ST1_ ΔK107 deletion was slightly more deleterious than the K107T substitution (EC_50_ 20 nM vs 5 nM), while 221-5’s resistance was comparable between the two (10 nM). Conversely, the OspA_ST1_ K107T and ΔK107 reporter strains remained sensitive to killing by 221-20, demonstrating the unique nature of 221-20’s paratope-epitope interaction and differentiating 221-20 from the other two Bin 1 mAbs.

In light of these results, we examined whether introduction of Lys-107 in an otherwise resistant OspA variant from *B. valaisiana* (IST15) was sufficient to render cells susceptible to killing by the Bin 1 mAbs. In accordance with that hypothesis, the OspA_IST15__G106insK reporter strain was susceptible to all Bin 1 mAbs with EC_50_ values comparable to OspA_ST1_ (EC_50_ range 0.63- 2.50 nM), thereby demonstrating that antibody recognition is transformed by the addition of a single Lys residue at position 107. We also generated a reporter strain with a *B. turdi* OspA (IST16) allele encoding a T107K substitution to investigate the role of Lys-107 in the context of polymorphisms elsewhere in 857-2 and other Bin 1 mAb epitopes (e.g., residues 128–132). In fact, the T107K substitution was sufficient to render *B. turdi* OspA at least partially susceptible to the **Class I** mAbs (857-2, 221-5, 221-20) and one **Class II** mAb (223-5) with EC_50_ values ranging from 10-40 nM (**Fig 8**). Collectively, these experiments demonstrate that Lys-107 is a key susceptibility determinant for virtually all Bin 1 mAbs.

## Discussion

The development of cross-protective vaccines for Lyme disease has been challenging due to the antigenic variability of OspA among the major pathogenic *B. burgdorferi* genospecies in North America, Europe, and Asia. Efforts to identify conserved epitopes on OspA have been limited, despite evidence in the literature for their existence [17,44]. Wilske and colleagues, for example, described a mouse mAb (L31 1F11) capable of reacting with all seven OspA STs and more than 100 primary isolates [15]. L31 1F11’s epitope was localized through a series of OspA truncations and Western blotting to residues 102–111, which corresponds to OspA’s central β-sheet [15]. Twenty years later, Wang and colleagues “rediscovered” this region of OspA in their effort to identify broadly reactive human mAbs with the potential to serve as preexposure prophylactics for Lyme disease [11]. Indeed, in our current study we identified overlapping protective B cell epitope(s) that are shared across all the major OspA serotypes associated with Lyme disease-causing spirochetes. The core of those epitopes involves residues within OspA_ST1_ β-strands 6–10 and loops 7–8 and 9–10 (**S10 Fig**). Within this region, there are invariant (e.g., Glu-100, Asp-105) and highly conserved residues responsible for cross-serotype recognition. Varying paratope interactions within and outside this core epitope account for the different degrees of mAb reactivity across the seven major OspA serotypes. For example, single amino acid polymorphisms relative to OspA_ST1_ accounts for the reduced binding of 221-11 (**Class II**) and 227-1 (**Class III**) to OspA_ST3_ and OspA_ST7_. Nonetheless, these results suggest that it may be possible to design a “universal” monovalent Lyme disease vaccine antigen focused on OspA’s central β-sheet as an alternative strategy to multivalent chimeric vaccines derived from the variable C-terminal regions of the major OspA serotypes [23–26].

Also relevant to vaccine immunology, we observed a predilection of particular V_H_ and VL combinations for recognition of OspA’s central β-sheet. Namely, the seven Bin 1 mAbs characterized in this study as well as 221-7 are predicted to utilize V_H_ germline HV5–51 with critical epitope interactions mediated largely (if not exclusively) by germline encoded residues. Similarly, critical light chain contacts are associated with KV1–13 or KV3–11 germline residues, suggesting that particular BCR pairings (i.e., HV5–51/ KV1–13) may be sufficient to engage with conserved residues within OspA’s central β-sheet, including Lys-107. However, as these Bin 1 mAbs were generated from hybridomas derived from hyperimmunized transgenic mice containing human immunoglobulin genes [11], we can only speculate that such interactions are clinically relevant. BCR repertoire analysis and the role of unmutated, class switch B cells and particular germline in response to Lyme disease has garnered attention recently [45–47]. Jiang and colleagues examined BCR usage and frequency of somatic hypermutation in memory B cell populations from erythema migrans skin lesions and noted a subpopulation of unmutated memory B cells [46]. Blum and colleagues examined BCR repertoires in circulating plasmablasts and, perhaps coincidently identified HV5–51 as being one of just two V_H_ germlines overrepresented in Lyme disease patients, as compared to healthy controls [45].

The generation of isogenic *B. burgdorferi* reporter strains expressing the seven major OspA serotypes (ST1–7) and numerous ISTs proved instrumental in being able to define cross-borreliacidal epitopes recognized by the seven mAbs in this study. We speculate that the same reporters would be equally valuable in assessing antibody responses elicited by OspA-based vaccines. At present, assessing complement-dependent bactericidal activity across different Bbsl genospecies requires the development of genospecies-specific assays in which culture conditions, complement sources, and assay duration must be optimized for each individual strain [48,49]. For example, because of *B. garinii*’s (ST3) inherent sensitivity to guinea pig, mouse and human complement, Comstedt and colleagues had resorted to freshly isolated chicken blood for bactericidal assays [25]. Moreover, the overwhelming reliance on ATP-based viability assays is problematic due to the nature of *B. burgdorferi* metabolism and high signal to noise ratios. The adoption of a standardized fluorescence-based, serum borreliacidal assay (SBA) based on OspA reporter strains like those described herein would not only streamline the assay itself but would enable different manufacturers and regulatory agencies to compare vaccine efficacies using a single platform.

## Materials and methods

### Cloning and expression of OspA and anti-OspA mAbs

Recombinant human monoclonal IgG1 antibodies were purified via Protein A affinity column from Expi293 cells that had been transiently transfected with V_H_ and V_L_ plasmids, as described [11]. Recombinant OspA_ST1_ (residues 18–273) from *B. burgdorferi* B31 (NCBI:txid224326) was expressed in *Escherichia coli* BL21-DE3 and purified as described [12]. Purified OspA was maintained in 20 mM HEPES, 150 mM NaCl, 20 mM imidazole, pH 7.50. Recombinant non-lipidated OspA serotypes 1–7 purified from *E.coli* were kindly provided by Dr. Meredith Finn (Moderna) and described elsewhere [34].

### ELISA

Immulon 4HBX plates (ThermoFisher, Waltham, MA) were coated overnight at 4°C with 1 µg/mL of the seven OspA serotypes (non-lipidated) and the next morning blocked for 2 h with 2% goat serum in PBS supplemented with 0.1% Tween-20 (PBST). Serial two-fold dilutions of each antibody starting from 1 µg/mL were made in separate dilution plates, and transferred to the serotype coated plates for 1 h. Plates were then washed three times with PBST, and a goat anti-human HRP conjugated secondary antibody (SouthernBiotech) was added for 1 h. Plates were then washed three times with PBST, developed with SureBlue TMB substrate (Seracare, Milford, MA), and quenched with 1M phosphoric acid. Absorption at 450 nm was read by a SpectraMax iD5 spectrophotometer using Softmax Pro v7.1 (Molecular Devices, San Jose, CA).

### Affinity measurements with BioLayer interferometry

Certain monoclonal antibody binding kinetics first reported by Haque and colleagues [32] have been revised to reflect refined and optimized BLI protocols using an Octet RED96e (Sartorius AG, Gottingen, Germany). AHC sensors (#18–5060, Sartorius) were used to capture mAbs at 0.4 µg/mL for 5 min, to a loading level of ~0.5 nm, then dipped into wells containing a three-fold dilution series ofnon-lipidated OspA (ranging from 0.6 µg/mL, 21.58 nM to 0.0025 µg/mL, 0.089 nM) for 10 min. Finally, sensors were dipped into buffer alone for 30 minutes to measure dissociation. Buffer used in all wells was 0.1% BSA in PBS, with 0.01% Tween20. Sensors were regenerated before the first mAb and between each mAb with 0.2 M glycine, pH 2.2. Traces were background corrected with a reference sensor, in which mAb was loaded but not exposed to OspA, and a reference sample, in which a bare sensor was exposed to the highest concentration of OspA. Traces for each mAb were fit to a global 1:1 model. k_obs_ vs concentration was linear for each system.

### Construction of *B. burgdorferi* strains expressing OspA serotypes

The IPTG-inducible *mscarlet-I* viability reporter plasmid, pGW189 [34] was modified to facilitate the expression of *ospA* variants of interest under the regulatory control of the *P*_*ospAB*_ promoter from *B. burgdorferi* strain B31. To accomplish this, transcription terminators were first introduced into pGW189 to prevent read through transcription from the *lacI* and *mscarlet*-*I* ORFs. The lambda_T0_rrnBT1 and rrnbT2 transcription terminators were amplified from plasmids pTN7 [50] and puc18_mtn7-lacZ_gentR [51] using NEB’s Q5 DNA polymerase and tailed primers listed in **S5 Table**. Amplified DNA fragments were then assembled into pGW189 immediately downstream of the *lacI* and *mscarlet*-I ORFs using the SacI/HindIII restriction sites and NEB’s HiFi Assembly kit, creating pGW206.

The *ospA* promoter and ORF from *B. burgdorferi* B31 were next amplified in two overlapping DNA fragments with tailed primers designed to introduce a silent SphI restriction site ~50 bases into the *ospA* ORF and permit assembly into pGW206 via the PvuI and PvuII restriction sites. The resulting viability reporter/OspA ST1 expression plasmid, pGW217, subsequently served as the basis for creating additional OspA expression plasmids. To accomplish this, pGW217 was first digested with SphI and PvuII to remove the *ospA*_ST1_ ORF immediately following the conserved lipoprotein insertion sequence (amino acid residue 17). Gene fragments from additional *ospA* serotypes/*Bbsl* genospecies were then synthesized (IDT) and assembled directly into the vector using HiFi DNA Assembly kit (NEB).

Silent mutations were introduced into *ospA* gene sequences when necessary to reduce sequence complexity for *in vitro* synthesis. Mutations were introduced at sites that resulted in a shift to the most common or second most common codon utilized by *B. burgdorferi* B31. The codon adaptive index for each *ospA* cDNA was assessed prior to synthesis using JCAT [52] to ensure that the introduced silent mutations did not reduce translation efficiency in *B. burgdorferi.*

Gene fragments were also synthesized to create *B. burgdorferi* B31, *B. valaisiana,* and *B. turdi* reporter plasmids with insertions, deletions, or substitutions at position 107 within the *ospA* ORF. To permit direct comparison with *B31* point mutants, a second “wild type” OspA ST1 reporter plasmid was also created containing the same silent mutations that were introduced to increase GC content/reduce sequence complexity for *in vitro* synthesis. The sequences of primers and synthesized *ospA* gene fragments used in this study are provided in **S5** **and** **S6 Tables**.

OspA reporter plasmids were sequenced and transformed into *B. burgdorferi* HB19-R1 [37], a high-passage derivative of the human blood isolate HB19 that lacks linear plasmid 54 (*ospAB*⁻), using previously described methods [53]. Transformants were selected in liquid BSKII supplemented with gentamicin (100 µg/ml) and individual clones were isolated through serial dilution [53]

To evaluate native plasmid content in transformants, the plasmid profiles of *B. burgdorferi* HB19 and HB19-R1 were characterized using NEB’s Taq Quickload 2 × PCR MM with established *B. burgdorferi* plasmid profiling primer sets [54]. Additional primer sets targeting cp26, lp21, lp28–1, lp5, lp28–5, and lp28–6 were also included in the analysis [54,55].Since plasmid content was uniform among clones transformed with mScarlet-I reporter plasmids carrying *ospA* ST1–7 (pGW217–pGW223, pGW250), plasmid profiling was not performed on HB19-R1 reporter strains encoding OspA ISTs due to the labor-intensive nature of the screening process. All *Bbsl* strains used or constructed in this study are listed in **S1** **and** **S2 Tables**.

### Flow cytometry-based antibody binding to strains

To analyze the ability of Bin 1 mAbs to bind to native OspA ST1–7 on the bacterial surface of primary isolates, *Borrelia* strains *B. burgdorferi* B31 (ST1), *B. afzelii* Pko (ST2), *B. garinii* PBr (ST3), *B. bavariensis* PBi (ST4), *B. garinii* PHei (ST5), *B. garinii* TN (ST6), and *B. garinii* T25 (ST7) (kindly provide by Dr. John Leong, Tufts University) were cultured at 33°C with 5% CO_2_ until mid-log phase. Bacterial cells were stored at -80ºC in fresh media containing 20% glycerol, and preparation, incubations, and flow cytometry analysis was performed as described [47]. To analyze the ability of Bin 1 mAbs to bind to OspA ST1–7 on the bacterial surface of reporter strains HB19-R1, (GGW1072–1079; **S2 Table**), bacterial cells were cultured at 33°C with 5% CO_2_ in BSKII media, minus gelatin and containing gentamycin 50 µg/ml, until mid-log phase. Bacterial cell preparation, incubations, and flow cytometry analysis was performed as described [56]. The *Salmonella*-specific mAb, Sal4 IgG, was used as an isotype control [57].

### Complement-dependent borreliacidal assays

Complement-dependent bactericidal assays were performed as previously described [34] with several modifications. Because guinea pig and human-derived complement perform equivalently in this assay with respect to sensitivity and reproducibility (**S11 Fig**), all experiments were conducted using guinea pig complement due to financial constraints. Frozen aliquots of OspA-expressing HB19-R1 reporter strains were thawed at room temperature and 500 µl from each stock (equivalent to 5x10^7^ spirochetes) were transferred to 50 ml conical tubes containing 45 ml of in gelatin-free BSK II supplemented with 50 µg/ml of gentamicin. Tubes were then sealed, and the cultures were incubated at 33ºC under static growth conditions. Three days later, spirochetes were collected via centrifugation (4,000 x *g*), the supernatant was removed, and cell pellets were resuspended in phenol red-free BSK II with gentamicin (50 µg/ml) at a density of 3 x 10^7^ spirochetes/ml.

Spirochetes were next seeded into white bottom 96 well microtiter assay plates (Costar) and mixed 1:1 with serial dilutions of anti-OspA_ST1_ mAbs prepared in phenol red-free BSKII containing 5% guinea pig complement (Sigma-Aldrich) and gentamicin (50 µg/ml), resulting in 3x10^6^ spirochetes per well with a final complement concentration of 2.5% and antibody concentrations between 10.00 nM - 0.08 nM, or 20 nM - 0.16 nM (depending on strain/experiment. Eight untreated (antibody-free) control reactions per strain were also included on each assay plate to determine baseline and peak fluorescence for data normalization.

Following antibody addition, assay plates were incubated overnight at 37ºC with 5% CO_2_ without agitation. The next day (18–20 h later), 1 mM IPTG was added to appropriate wells to induce expression of *mScarlet* in surviving spirochetes. Assay plates were then returned to the incubator for another 24 h period. The following day, median fluorescence intensity (MFI) was measured three times per plate at 569 nm (excitation) and 611 nm (emission) using a Spectromax ID3 plate reader (Molecular Biosystems) and Softmax pro version 7.1 software.

Mean MFI data was independently normalized for each assay plate using the untreated/non-induced and untreated/IPTG-induced controls to establish baseline (0) and peak (1) MFI. Graphpad Prism version 9.5.1 was then used to generate killing curves and heatmaps, while radar plots were constructed in Microsoft Excel. Ec_50_ values were assigned to the lowest antibody dilution resulting in >50% reduction in MFI relative to untreated controls. Ec_50_ values reported in heatmaps and radar plots reflect the mean calculated using normalized MFI data from three to five independent experiments. Strains that exhibited resistance to anti-OspA Bin 1 mAbs at the highest concentration tested (10 or 20 nM) were subjected to additional bactericidal assays with 10 µg/ml of the antibodies in question. Resistance at this dosage is shown as “>60 nm” in heat maps and radar plots.

### Cloning, expression and purification of OspA and anti-OspA Fabs

The PCR amplicon encoding *B. burgdorferi* OspA residues 18–273 was subcloned in frame into the pSUMO expression vector with an N-terminal deca-histidine and SUMO tag. All cloning was performed using a standard ligase independent cloning protocol. OspA was expressed in *E. coli* strain BL21 (DE3). The transformed bacteria were grown at 37°C in TB medium and induced at 20°C with 0.1 mM (IPTG) at an OD_600_ of 0.6 for ~16 hours at 20°C. After induction, cells were harvested and resuspended in 20 mM Hepes pH 7.5 and 150 mM NaCl. The cell suspension was sonicated and centrifuged at 30,000 g for 30 minutes. After centrifugation, the protein-containing supernatant was purified by nickel-affinity and size-exclusion chromatography on an AKTAxpress system (GE Healthcare), which consisted of a 1mL nickel affinity column followed by a Superdex 200 16/60 gel filtration column. The elution buffer consisted of 0.5M imidazole in binding buffer, and the gel filtration buffer consisted of 20mM Hepes pH 7.5, 150mM NaCl, and 20mM imidazole. Fractions containing OspA (18–273) was pooled and subject to TEV protease cleavage (1:10 weight ratio) for 3 hours at room temperature in order to remove their respective fusion protein tags. The cleaved protein was passed over a 1mL Ni-NTA agarose (Qiagen) gravity column to remove the added TEV protease, cleaved residues, and uncleaved fusion protein. Each IgG was subjected to papain digestion followed by affinity depletion of the Fc fragment by Protein A FPLC chromatography to generate each Fab. After purification, each Fab and OspA were mixed in a 1:1 stoichiometry to form a stable complex concentrated to a final concentration of 10 mg/ml for all crystallization trials.

### Crystallization and data collection

Fab-OspA crystals were grown by sitting drop vapor diffusion at 4°C using a protein to reservoir volume ratio of 1:1 with total drop volumes of 0.2 μl. Crystals of each OspA-Fab complex were produced using the crystallization solutions shown in **S3 Table**. All crystals were flash frozen in liquid nitrogen after a short soak in the appropriate crystallization buffers supplemented with 25% ethylene glycol. Data were collected at the 24-ID-E beamline at the Advanced Photon Source, Argonne National Labs. Data resolution limit was determined based on the I/s criterion which was 2.0 in the highest resolution shell. All data was indexed, merged, and scaled using HKL2000 [58] then converted to structure factor amplitudes using CCP4 [59].

### Structure determination and refinement

The structure of each Fab-OspA complex was solved by molecular replacement using Phaser [58]. Molecular replacement calculations were performed using the Fab coordinates from PDB code 2XTJ as the search model for Fab 221-5, the Fab heavy chain coordinates from PDB code 4RIR along with the Fab light chain coordinates from PDB code 4M6O were independently used as search models for Fabs 221-11 and 857-2. The Fab coordinates from PDB code 4M6O were also used as the search model for Fab 227-1. OspA coordinates (PDB code 1FJ1) were used as the search model for OspA in all four Fab-OspA complex structures solved. The resulting phase information from molecular replacement was used for manual model building of the Fab-OspA model using the graphics program COOT [60] and structural refinement employing the PHENIX package [61]. Data collection and refinement statistics are listed in **S4 Table**. Molecular graphics were prepared using PyMOL (Schrodinger) (DeLano Scientific LLC, Palo Alto, CA). The structures generated in this study were deposited in the Protein Data Bank (PDB; http://www.rcsb.org/pdb/) under accession numbers shown in **Table 2**.

### Phylogenetic tree construction

A collection of 135 OspA protein sequences containing representatives from 23 recognized Bbsl genospecies was compiled from borreliabase.org, NCBI’s OspA IST typing database (PubMLST), and Bacterial and Viral Bioinformatics Resource Center (BV-BRC.org). MUSCLE (accessed via EMBL) was then used to generate a multiple sequence alignment of the collection. The resulting MSA file was then used to construct an unrooted approximately maximum likelihood phylogenetic tree using Fasttree and LG modelling (accessed via BV-BRC.ORG). The resulting phylogenetic tree was then formatted and visualized using ITOL version 7 [62]. Files associated with phylogenetic tree construction are provided in **S1 File**.

### OspA multiple sequence alignment (MSA)

A MSA was created containing one representative sequence from each of the 33 OspA clades/types identified through phylogenetic analysis. OspA variants selected for the alignment included OspA sequences utilized in viability reporter strain construction and OspA sequences from type or reference strains (when available). Sequence alignment was performed using the MAFFT algorithm with OspA from *B. burgdorferi* B31 as a reference. Following alignment, the Bin 1 epitope region (β strands 6–10, residues 82–146) was visualized using the MSA viewer interface on BV-BRC.org. A graphic of the alignment was then created with the Taylor color scheme and the sequence logo displayed to emphasize conservation between OspA types/clades.

### Microsphere immunoassay (MIA)

ST 1–7 and OspB were coupled to Magplex-C microspheres (5mg antigen/ 1x10^6^ microspheres) using a xMap Antibody Coupling Kit as recommended by the manufacturer (Luminex Corporation, Austin, TX). Successful coupling of ST 1–7 and OspB was confirmed using LA-2 and H6831, respectively. Beads were protected from light and stored 2–8°C in xMAP AbC Wash Buffer (5x10^6^ microspheres/mL) until use.

OspA Bin 1 mAbs were serially diluted (1:2) in assay buffer (1 x PBS, 2% BSA, pH 7.4) in round bottom, non-treated plates (Costar, Kennebunk, Maine) with starting concentrations of 2.5 mg/mL (223-5), 0.625 mg/mL (857-2, 221-5, 221-20, 221-11, 227-1), or 0.3125 mg/mL (227-2). Coupled microsphere stocks were diluted (1:50) in assay buffer and added (50 μL/well) to black, clear-bottomed, non-binding, chimney 96-well plates (Greiner Bio-One, Monroe, North Carolina). The mAb dilutions (50 μL) were combined with the microspheres and incubated at room temperature for 1 hr in a tabletop shaker (600 rpm). Plates were placed on a magnetic separator and washed three times using wash buffer (1 x PBS, 2% BSA, 0.02% TWEEN-20, 0.05% Sodium azide, pH 7.4). PE labeled goat anti-Human IgG Fc, eBioscience (Invitrogen, Carlsbad, California) secondary antibody diluted 1:500 in assay buffer was added (100 μL/well) and incubated at room temperature for 30 min in a tabletop shaker (600 rpm). Plates were washed as previously stated. The microspheres were resuspended in 100 μL of wash buffer and placed back on the tabletop shaker (600 rpm) for 5 minutes prior to analysis using a FlexMap 3D (Luminex Corporation).

## Supporting information

S1 Table *Borrelia burgdorferi sensu lato s*trains used in this study. (DOCX)

S2 Table Recombinant *B. burgdorferi* strains used in this study. (DOCX)

S3 Table OspA-Fab crystallization solutions. (DOCX)

S4 Table Summary of OspA-Fab structures in this study. (DOCX)

S5 Table PCR primers used in this study. (DOCX)

S6 Table Sequences of *ospA* gene fragments synthesized for reporter construction. (DOCX)

S1 Fig 857-2 binds to OspA serotypes 1–7 expressed on the surface of *Borrelia* primary isolates. Representative flow cytometric analysis of live *Borrelia* primary isolates expressing OspA serotypes (ST) 1–7 with 10 µg/ml of Bin 1 IgG mAb 857-2, followed by addition of Alexa 647-labeled goat anti-human IgG secondary antibody. An isotype control, O5 antigen-specific IgG variant Sal4, was run with each ST, however ST 1 is shown as the representative. OspA ST 1-specific IgG mAb LA-2 was run with ST 1–7 for comparison. The horizontal bracket represents the region on subsequent plots that are positive for fluorescence labeling (647+). The percentage of events positive for Alexa 647 fluorescence labeling and the geometric mean fluorescence intensity (gMFI) are shown. Compared to isotype controls, gMFI values of LA-2 bound to ST1, and 857-2 bound to ST1–7 are significantly different (p < 0.0001). There is no significant difference in gMFI with LA-2 and ST2–7 strains (p > 0.9999). N = 3. Statistical comparisons were performed using two-way ANOVA with Dunnet’s multiple-comparison test. Image was generated in FlowJo. ST 1-*B. burgdorferi* B31; ST 2- *B. afzelii* Pko; ST 3- *B. garinii* PBr; ST 4- *B. bavariensis* PBi; ST 5-*B. garinii* PHei; ST 6- *B. garinii* TN; ST 7- *B. garinii* T25.
(PDF)

S2 Fig Binding of Bin 1 mAbs to recombinant OspA ST1–7. Bin 1 IgG mAbs were subjected to MIA using non-lipidated recombinant ST 1–7 coupled microspheres as detailed in the materials and methods. Recombinant OspB was included as a negative control. Median fluorescent intensity (MFI) is displayed on the y-axis and mAb concentration is shown on the x-axis. The plotted lines show the mean MFI and standard deviation for each serotype across 3 technical replicates with each point representing a single experiment. The concentrations shown are two-fold serial dilutions with starting concentrations of 2.5 µg/mL (223-5), 0.625 µg/mL (857-2, 221-5, 221-20, 221-11, 221-7, 227-1), or 0.3125 µg/mL (227-2).
(PDF)

S3 Fig Anti-OspA_ST1_ Bin1 mAbs promote complement-dependent killing of recombinant *B. burgdorferi* Strains expressing OspA ST1–7. (A) Antibody titration curves depicting bactericidal activity against *B. burgdorferi* HB19-R1 reporter strains expressing OspA_ST1–7_. Complement-dependent bactericidal assays were performed with anti-OspA_ST1_ Bin1 mAbs and *B. burgdorferi* HB19-R1 strains harboring an IPTG-inducible mscarlet-I viability reporter plasmid expressing *ospA* serotypes 1–7 as described in the material and methods. Experimental controls included an HB19-R1 strain carrying the IPTG-inducible viability reporter plasmid without an *ospA* variant and a mAb with bactericidal activity restricted to OspA_ST1_ (LA-2). The data shown encompasses 3–5 independent experiments per strain with data normalized as described within the materials and methods section. (B) Heat map summarizing statistical comparison of differences in susceptibility to anti-OspA ST1 mAbs between the HB19-R1 OspA_ST1_ reporter strain and reporter strains expressing OspA_ST2–7_. Statistical analyses were performed via one-way ANOVA followed by Dunnett’s multiple-comparison test. Significant differences in susceptibility are denoted by purple or pink shading.
(PDF)

S4 Fig Structural similarity of OspA. Superpositioned Cα-traces of the unliganded form of OspA (PDB ID: 2G8C) colored green with OspA form all four Fab-OspA structures (shaded from light to dark gray) depicting the structural similarity of OspA across these different structures.
(PDF)

S5 Fig OspA sequence alignments. The high sequence similarity between OspA residues in *B. burgdorferi* (ST1) that interact with Fabs 857-2, 221-5, 221-11, 227-1 and the OspA sequence conservation from serotypes 2–7, supports our observed cross-reactivity of mAbs 857-2, 221-5, 221-11, 227-1 across nearly all seven serotypes. Sequence alignment of the interacting regions of OspA from *B. burgdorferi* (ST1) with (A) 857-2 (B) 221-5 (C) 221-11 (D) 227-1 aligned with OspA sequences ST2-ST7. The yellow boxes in all four panels highlight residue positions 131 and 152, which mark primary sequence differences among OspA serotypes that influence antibody cross-reactivity. In ST 3 and 7, residue 131 is a lysine (Lys-131) rather than a glutamate (Glu-131), and residue 152 is an asparagine rather than a serine. These substitutions appear to underlie the reduced cross-reactivity of Fabs 221-11 and 227-1 with ST3 and ST7. Magenta asterisks depict VL interacting residues, cyan asterisks depict VH interacting residues, and blue asterisks denote interaction with VL and VH residues. Secondary structural elements (β-strands 4–14 in panels A,B,C and β-strands 4–19 in panels D) are drawn as black arrows above the sequence and labelled accordingly. Red colored sequence denotes sequence identity and white colored sequence shows reduced sequence similarity. Figure made with ClustalW and ESPript 3.0.
(PDF)

S6 Fig Inferred germline usage. (A) Heavy chains of the 8 Bin 1 mAbs aligned to the HV5–51*01 Germline Gene. (B) Light chains of 221-5 and 221-20 aligned to KV3–11*01 Germline Gene. (C) Light chains of the other six mAbs aligned to KV1–13*02 Germline Gene.
(PDF)

S7 Fig Multiple sequence alignment depicting total sequence diversity within the Bin1 epitope region of 33 OspA types. Representative sequences from each OspA clade were aligned via MAFFT using OspA from *B. burgdorferi* strain B31 as a reference. Bolded strain names indicate OspA variants expressed by HB19-R1 viability reporter strains and subjected to complement-dependent bactericidal assays with anti-OspAST1 Bin 1 mAbs. Amino acid residues numbered in white font denote conservation within the epitopes of two or more Bin1 mAbs (857-2, 221-5, 221-11, 221-7, and 227-1). Numbered amino acid residues with colored background form critical interactions with residues within the paratopes of Bin1 mAbs with solved crystal structures.
(PDF)

S8 Fig Genetically diverse OspA types are susceptible to complement-mediated killing by anti-OspA_ST1_ Bin1 mAbs. (A) Antibody titration curves depicting complement-dependent killing of *B. burgdorferi* HB19-R1 reporter strains expressing OspA *in silico* types (ISTs). Complement-dependent killing assays were performed as described in the materials in methods section with anti-OspA Bin1 mAbs and HB19-R1 viability reporter strains expressing diverse OspA ISTs. The data shown encompasses at least three independent experiments per strain with data normalized as described. (B) Heat map summarizing statistical comparison of differences in Bin1 mAb susceptibility between the HB19-R1 OspA_ST1_-expressing reporter strain and reporter strains expressing all other OspA variants as determined via one-way ANOVA followed by Dunnett’s multiple-comparison test. Significant differences in susceptibility relative to OspA_ST1_ are denoted by purple or pink shading.
(PDF)

S9 Fig Lys-107 polymorphisms are a determinant of Bin1 mAb susceptibility. (A) Antibody titration curves summarizing complement-dependent killing of HB19-R1 viability reporter strains expressing OspA variants with polymorphisms at position 107. Complement-dependent killing assays were performed as described in the materials and methods section using anti-OspA Bin1 mAbs and *B. burgdorferi* HB19-R1 viability reporter strains expressing “WT” OspA variants from *B. burgdorferi B31* (ST1), *B. valaisiana* VS116 (IST15), and *B. turdi* TPT2017 (IST16), or mutated derivatives containing deletions, insertions, or substitutions at residue 107. (B) Susceptibility of HB19-R1 reporter strains harboring OspA variants with polymorphisms at position 107 to complement dependent killing mediated by 66.7 nM of Class II & Class III Bin 1 mAbs. All data shown includes at least three independent experiments per strain with data normalized as described.
(PDF)

S10 Fig OspA β-Strand Labels, and Combined Epitope of Bin 1 mAbs. Ribbon diagram of OspA depicting close up of the CBS with residues conserved within the epitopes of 857-2, 221-5, 221-11, and 227-1 labeled and shaded in blue. Strand numbers are labeled in red.
(PDF)

S11 Fig Human and Guinea Pig-derived complement can be used interchangeably in SBAs with *B. burgdorferi* HB19-R1 reporter strains. Complement-dependent bactericidal assays were performed using HB19-R1 reporter strains and mAb 857-2 with either 2.5% guinea pig complement or 5% human complement (Pel-Freeze), under conditions described in the materials and methods section. Data shown encompasses 3–5 independent experiments. Differences in strain susceptibility to mAb 857-2 between complement sources were assessed across all strains by two-way ANOVA followed by Sidak’s multiple-comparisons test. No significant differences in susceptibility were observed.
(PDF)

S1 File Files associated with OspA phylogenetic tree construction. This dataset includes the multiple sequence alignment, phylogenetic tree, and phylogenetic tree formatting files used to construct the OspA phylogenetic tree shown in Fig 6.
(7Z)

S2 File Vector map and sequence of pGW217. This dataset includes the vector map and annotated sequence file for the OspAST1 viability reporter plasmid used to construct all other OspA-expressing mscarlet-I reporter plasmids described in this study.
(ZIP)

## References

[1] Kugeler KJ, Schwartz AM, Delorey MJ, Mead PS, Hinckley AF. Estimating the frequency of Lyme disease diagnoses, United States, 2010-2018. Emerg Infect Dis. 2021;27(2):616–9. doi: 10.3201/eid2702.202731 33496229 PMC7853543

[2] Steere AC, Strle F, Wormser GP, Hu LT, Branda JA, Hovius JWR, et al. Lyme borreliosis. Nat Rev Dis Primers. 2016;2:16090. doi: 10.1038/nrdp.2016.90 27976670 PMC5539539

[3] Aucott JN, Yang T, Yoon I, Powell D, Geller SA, Rebman AW. Risk of post-treatment Lyme disease in patients with ideally-treated early Lyme disease: a prospective cohort study. Int J Infect Dis. 2022;116:230–7. doi: 10.1016/j.ijid.2022.01.033 35066160

[4] Steere AC, Grodzicki RL, Kornblatt AN, Craft JE, Barbour AG, Burgdorfer W, et al. The spirochetal etiology of Lyme disease. N Engl J Med. 1983;308(13):733–40. doi: 10.1056/NEJM198303313081301 6828118

[5] Steere AC, Sikand VK, Meurice F, Parenti DL, Fikrig E, Schoen RT, et al. Vaccination against Lyme disease with recombinant Borrelia burgdorferi outer-surface lipoprotein A with adjuvant. Lyme Disease Vaccine Study Group. N Engl J Med. 1998;339(4):209–15. doi: 10.1056/NEJM199807233390401 9673298

[6] Fikrig E, Barthold SW, Kantor FS, Flavell RA. Protection of mice against the Lyme disease agent by immunizing with recombinant OspA. Science. 1990;250(4980):553–6. doi: 10.1126/science.2237407 2237407

[7] Schaible UE, Kramer MD, Eichmann K, Modolell M, Museteanu C, Simon MM. Monoclonal antibodies specific for the outer surface protein A (OspA) of Borrelia burgdorferi prevent Lyme borreliosis in severe combined immunodeficiency (scid) mice. Proc Natl Acad Sci U S A. 1990;87(10):3768–72. doi: 10.1073/pnas.87.10.3768 2339119 PMC53984

[8] Simon MM, Schaible UE, Kramer MD, Eckerskorn C, Museteanu C, Müller-Hermelink HK, et al. Recombinant outer surface protein a from Borrelia burgdorferi induces antibodies protective against spirochetal infection in mice. J Infect Dis. 1991;164(1):123–32. doi: 10.1093/infdis/164.1.123 1829104

[9] Bockenstedt LK, Fikrig E, Barthold SW, Kantor FS, Flavell RA. Inability of truncated recombinant Osp A proteins to elicit protective immunity to Borrelia burgdorferi in mice. J Immunol. 1993;151(2):900–6. doi: 10.4049/jimmunol.151.2.900 8335917

[10] de Silva AM, Telford SR 3rd, Brunet LR, Barthold SW, Fikrig E. Borrelia burgdorferi OspA is an arthropod-specific transmission-blocking Lyme disease vaccine. J Exp Med. 1996;183(1):271–5. doi: 10.1084/jem.183.1.271 8551231 PMC2192397

[11] Wang Y, Kern A, Boatright NK, Schiller ZA, Sadowski A, Ejemel M, et al. Pre-exposure prophylaxis with OspA-specific human monoclonal antibodies protects mice against tick transmission of lyme disease spirochetes. J Infect Dis. 2016;214(2):205–11. doi: 10.1093/infdis/jiw151 27338767 PMC4918831

[12] Schiller ZA, Rudolph MJ, Toomey JR, Ejemel M, LaRochelle A, Davis SA, et al. Blocking Borrelia burgdorferi transmission from infected ticks to nonhuman primates with a human monoclonal antibody. J Clin Invest. 2021;131(11):e144843. doi: 10.1172/JCI144843 33914704 PMC8159683

[13] Fikrig E, Telford SR 3rd, Barthold SW, Kantor FS, Spielman A, Flavell RA. Elimination of Borrelia burgdorferi from vector ticks feeding on OspA-immunized mice. Proc Natl Acad Sci U S A. 1992;89(12):5418–21. doi: 10.1073/pnas.89.12.5418 1608951 PMC49303

[14] Wilske B, Luft B, Schubach WH, Zumstein G, Jauris S, Preac-Mursic V, et al. Molecular analysis of the outer surface protein A (OspA) of Borrelia burgdorferi for conserved and variable antibody binding domains. Med Microbiol Immunol. 1992;181(4):191–207. doi: 10.1007/BF00215765 1279368

[15] Wilske B, Preac-Mursic V, Göbel UB, Graf B, Jauris S, Soutschek E, et al. An OspA serotyping system for Borrelia burgdorferi based on reactivity with monoclonal antibodies and OspA sequence analysis. J Clin Microbiol. 1993;31(2):340–50. doi: 10.1128/jcm.31.2.340-350.1993 8432821 PMC262762

[16] Gern L, Hu CM, Voet P, Hauser P, Lobet Y. Immunization with a polyvalent OspA vaccine protects mice against Ixodes ricinus tick bites infected by Borrelia burgdorferi ss, Borrelia garinii and Borrelia afzelii. Vaccine. 1997;15(14):1551–7. doi: 10.1016/s0264-410x(97)00066-2 9330467

[17] Golde WT, Burkot TR, Piesman J, Dolan MC, Capiau C, Hauser P, et al. The Lyme disease vaccine candidate outer surface protein A (OspA) in a formulation compatible with human use protects mice against natural tick transmission of B. burgdorferi. Vaccine. 1995;13(5):435–41. doi: 10.1016/0264-410x(94)00027-k 7639011

[18] Schaible UE, Wallich R, Kramer MD, Gern L, Anderson JF, Museteanu C, et al. Immune sera to individual Borrelia burgdorferi isolates or recombinant OspA thereof protect SCID mice against infection with homologous strains but only partially or not at all against those of different OspA/OspB genotype. Vaccine. 1993;11(10):1049–54. doi: 10.1016/0264-410x(93)90132-h 8212826

[19] Koide S, Yang X, Huang X, Dunn JJ, Luft BJ. Structure-based design of a second-generation Lyme disease vaccine based on a C-terminal fragment of Borrelia burgdorferi OspA. J Mol Biol. 2005;350(2):290–9. doi: 10.1016/j.jmb.2005.04.066 15935380

[20] Federizon J, Frye A, Huang W-C, Hart TM, He X, Beltran C, et al. Immunogenicity of the Lyme disease antigen OspA, particleized by cobalt porphyrin-phospholipid liposomes. Vaccine. 2020;38(4):942–50. doi: 10.1016/j.vaccine.2019.10.073 31727504 PMC6980772

[21] Nayak A, Schüler W, Seidel S, Gomez I, Meinke A, Comstedt P, et al. Broadly protective multivalent OspA vaccine against lyme borreliosis, developed based on surface shaping of the C-terminal fragment. Infect Immun. 2020;88(4):e00917-19. doi: 10.1128/IAI.00917-19 31932330 PMC7093141

[22] Schubach WH, Mudri S, Dattwyler RJ, Luft BJ. Mapping antibody-binding domains of the major outer surface membrane protein (OspA) of Borrelia burgdorferi. Infect Immun. 1991;59(6):1911–5. doi: 10.1128/iai.59.6.1911-1915.1991 2037351 PMC257942

[23] Comstedt P, Hanner M, Schüler W, Meinke A, Lundberg U. Design and development of a novel vaccine for protection against Lyme borreliosis. PLoS One. 2014;9(11):e113294. doi: 10.1371/journal.pone.0113294 25409015 PMC4237411

[24] Comstedt P, Hanner M, Schüler W, Meinke A, Schlegl R, Lundberg U. Characterization and optimization of a novel vaccine for protection against Lyme borreliosis. Vaccine. 2015;33(44):5982–8. doi: 10.1016/j.vaccine.2015.07.095 26277070

[25] Comstedt P, Schüler W, Meinke A, Lundberg U. The novel Lyme borreliosis vaccine VLA15 shows broad protection against Borrelia species expressing six different OspA serotypes. PLoS One. 2017;12(9):e0184357. doi: 10.1371/journal.pone.0184357 28863166 PMC5581183

[26] Kamp HD, Swanson KA, Wei RR, Dhal PK, Dharanipragada R, Kern A, et al. Design of a broadly reactive Lyme disease vaccine. NPJ Vaccines. 2020;5(1):33. doi: 10.1038/s41541-020-0183-8 32377398 PMC7195412

[27] Corti D, Voss J, Gamblin SJ, Codoni G, Macagno A, Jarrossay D, et al. A neutralizing antibody selected from plasma cells that binds to group 1 and group 2 influenza A hemagglutinins. Science. 2011;333(6044):850–6. doi: 10.1126/science.1205669 21798894

[28] Ekiert DC, Bhabha G, Elsliger M-A, Friesen RHE, Jongeneelen M, Throsby M, et al. Antibody recognition of a highly conserved influenza virus epitope. Science. 2009;324(5924):246–51. doi: 10.1126/science.1171491 19251591 PMC2758658

[29] Wu X, Yang Z-Y, Li Y, Hogerkorp C-M, Schief WR, Seaman MS, et al. Rational design of envelope identifies broadly neutralizing human monoclonal antibodies to HIV-1. Science. 2010;329(5993):856–61. doi: 10.1126/science.1187659 20616233 PMC2965066

[30] Julien J-P, Wardemann H. Antibodies against Plasmodium falciparum malaria at the molecular level. Nat Rev Immunol. 2019;19(12):761–75. doi: 10.1038/s41577-019-0209-5 31462718

[31] Lanzavecchia A, Frühwirth A, Perez L, Corti D. Antibody-guided vaccine design: identification of protective epitopes. Curr Opin Immunol. 2016;41:62–7. doi: 10.1016/j.coi.2016.06.001 27343848

[32] Haque HME, Ejemel M, Vance DJ, Willsey G, Rudolph MJ, Cavacini LA, et al. Human B Cell Epitope Map of the Lyme Disease Vaccine Antigen, OspA. ACS Infect Dis. 2022;8(12):2515–28. doi: 10.1021/acsinfecdis.2c00346 36350351

[33] Rudolph MJ, Davis SA, Haque HME, Ejemel M, Cavacini LA, Vance DJ, et al. Structure of a transmission blocking antibody in complex with Outer surface protein A from the Lyme disease spirochete, Borreliella burgdorferi. Proteins. 2023;91(11):1463–70. doi: 10.1002/prot.26549 37455569 PMC10592432

[34] Vance DJ, Basir S, Piazza CL, Willsey GG, Haque HME, Tremblay JM, et al. Single-domain antibodies reveal unique borrelicidal epitopes on the Lyme disease vaccine antigen, outer surface protein A (OspA). Infect Immun. 2024;92(4):e0008424. doi: 10.1128/iai.00084-24 38470113 PMC11003225

[35] Lundberg U, Hochreiter R, Timofoyeva Y, Kanevsky I, Meinke A, Anderson AS, et al. Preclinical evidence for the protective capacity of antibodies induced by lyme vaccine candidate VLA15 in people. Open Forum Infect Dis. 2024;11(9):ofae467. doi: 10.1093/ofid/ofae467 39233712 PMC11372474

[36] Barbour AG, Tessier SL, Todd WJ. Lyme disease spirochetes and ixodid tick spirochetes share a common surface antigenic determinant defined by a monoclonal antibody. Infect Immun. 1983;41(2):795–804. doi: 10.1128/iai.41.2.795-804.1983 6192088 PMC264710

[37] Sădziene A, Rosa PA, Thompson PA, Hogan DM, Barbour AG. Antibody-resistant mutants of Borrelia burgdorferi: in vitro selection and characterization. J Exp Med. 1992;176(3):799–809. doi: 10.1084/jem.176.3.799 1339462 PMC2119346

[38] Lee JT, Li Z, Nunez LD, Katzel D, Perrin BS Jr, Raghuraman V, et al. Development of a sequence-based in silico OspA typing method for Borrelia burgdorferi sensu lato. Microb Genom. 2024;10(5):001252. doi: 10.1099/mgen.0.001252 38787376 PMC11165634

[39] Akther S, Mongodin EF, Morgan RD, Di L, Yang X, Golovchenko M, et al. Natural selection and recombination at host-interacting lipoprotein loci drive genome diversification of Lyme disease and related bacteria. mBio. 2024;15(9):e0174924. doi: 10.1128/mbio.01749-24 39145656 PMC11389397

[40] Barbour AG, Qiu W. Borreliella. In: Bergey’s manual of systematics of archaea and bacteria. 1–22.

[41] Clark KL, Leydet B, Hartman S. Lyme borreliosis in human patients in Florida and Georgia, USA. Int J Med Sci. 2013;10(7):915–31. doi: 10.7150/ijms.6273 23781138 PMC3675506

[42] Kim CM, Yun NR, Kim D-M. Case report: the first borrelia yangtzensis infection in a human in Korea. Am J Trop Med Hyg. 2021;106(1):45–6. doi: 10.4269/ajtmh.21-0052 34662863 PMC8733495

[43] Margos G, Sing A, Fingerle V. Published data do not support the notion that Borrelia valaisiana is human pathogenic. Infection. 2017;45(4):567–9. doi: 10.1007/s15010-017-1032-1 28573415

[44] Gern L, Rais O, Capiau C, Hauser P, Lobet Y, Simoen E, et al. Immunization of mice by recombinant OspA preparations and protection against Borrelia burgdorferi infection induced by Ixodes ricinus tick bites. Immunol Lett. 1994;39(3):249–58. doi: 10.1016/0165-2478(94)90166-x 8034340

[45] Blum LK, Adamska JZ, Martin DS, Rebman AW, Elliott SE, Cao RRL, et al. Robust B cell responses predict rapid resolution of lyme disease. Front Immunol. 2018;9:1634. doi: 10.3389/fimmu.2018.01634 30072990 PMC6060717

[46] Jiang R, Meng H, Raddassi K, Fleming I, Hoehn KB, Dardick KR, et al. Single-cell immunophenotyping of the skin lesion erythema migrans identifies IgM memory B cells. JCI Insight. 2021;6(12):e148035. doi: 10.1172/jci.insight.148035 34061047 PMC8262471

[47] Rudolph MJ, Muriuki BM, Chen Y, Vance DJ, Vorauer C, Piazza CL, et al. Germline encoded residues dominate the interaction of a human monoclonal antibody with decorin binding protein A of Borrelia burgdorferi. Front Immunol. 2025;16:1611828. doi: 10.3389/fimmu.2025.1611828 40692775 PMC12277278

[48] Bézay N, Wagner L, Kadlecek V, Obersriebnig M, Wressnigg N, Hochreiter R, et al. Optimisation of dose level and vaccination schedule for the VLA15 Lyme borreliosis vaccine candidate among healthy adults: two randomised, observer-blind, placebo-controlled, multicentre, phase 2 studies. Lancet Infect Dis. 2024;24(9):1045–58. doi: 10.1016/S1473-3099(24)00175-0 38830375

[49] Wressnigg N, Pöllabauer E-M, Aichinger G, Portsmouth D, Löw-Baselli A, Fritsch S, et al. Safety and immunogenicity of a novel multivalent OspA vaccine against Lyme borreliosis in healthy adults: a double-blind, randomised, dose-escalation phase 1/2 trial. Lancet Infect Dis. 2013;13(8):680–9. doi: 10.1016/S1473-3099(13)70110-5 23665341

[50] Jasinska W, Manhart M, Lerner J, Gauthier L, Serohijos AWR, Bershtein S. Chromosomal barcoding of E. coli populations reveals lineage diversity dynamics at high resolution. Nat Ecol Evol. 2020;4(3):437–52. doi: 10.1038/s41559-020-1103-z 32094541

[51] Choi K-H, Gaynor JB, White KG, Lopez C, Bosio CM, Karkhoff-Schweizer RR, et al. A Tn7-based broad-range bacterial cloning and expression system. Nat Methods. 2005;2(6):443–8. doi: 10.1038/nmeth765 15908923

[52] Grote A, Hiller K, Scheer M, Münch R, Nörtemann B, Hempel DC, et al. JCat: a novel tool to adapt codon usage of a target gene to its potential expression host. Nucleic Acids Res. 2005;33(Web Server issue):W526-31. doi: 10.1093/nar/gki376 15980527 PMC1160137

[53] Samuels DS, Drecktrah D, Hall LS. Genetic transformation and complementation. Methods Mol Biol. 2018;1690:183–200. doi: 10.1007/978-1-4939-7383-5_15 29032546 PMC5806694

[54] Iyer R, Kalu O, Purser J, Norris S, Stevenson B, Schwartz I. Linear and circular plasmid content in Borrelia burgdorferi clinical isolates. Infect Immun. 2003;71(7):3699–706. doi: 10.1128/IAI.71.7.3699-3706.2003 12819050 PMC161973

[55] Xiang X, Yang Y, Du J, Lin T, Chen T, Yang XF. Investigation of ospC expression variation among Borrelia burgdorferi strains. Front Cell Infect Microbiol. 2017;7:131. doi: 10.3389/fcimb.2017.00131 28473966 PMC5397415

[56] Rudolph MJ, Davis SA, Haque HME, Weis DD, Vance DJ, Piazza CL, et al. Structural elucidation of a protective B cell epitope on outer surface protein C (OspC) of the lyme disease spirochete, Borreliella burgdorferi. mBio. 2023;14(2):e0298122. doi: 10.1128/mbio.02981-22 36976016 PMC10128040

[57] Richards AF, Doering JE, Lozito SA, Varrone JJ, Willsey GG, Pauly M, et al. Inhibition of invasive salmonella by orally administered IgA and IgG monoclonal antibodies. PLoS Negl Trop Dis. 2020;14(3):e0007803. doi: 10.1371/journal.pntd.0007803 32203503 PMC7117778

[58] Otwinowski Z, Minor W. Processing of X-ray diffraction data collected in oscillation mode. Methods Enzymol. 1997;276:307–26. doi: 10.1016/S0076-6879(97)76066-X 27754618

[59] Winn MD, Ballard CC, Cowtan KD, Dodson EJ, Emsley P, Evans PR, et al. Overview of the CCP4 suite and current developments. Acta Crystallogr D Biol Crystallogr. 2011;67(Pt 4):235–42. doi: 10.1107/S0907444910045749 21460441 PMC3069738

[60] Emsley P, Lohkamp B, Scott WG, Cowtan K. Features and development of Coot. Acta Crystallogr D Biol Crystallogr. 2010;66(Pt 4):486–501. doi: 10.1107/S0907444910007493 20383002 PMC2852313

[61] Adams PD, Afonine PV, Bunkóczi G, Chen VB, Davis IW, Echols N, et al. PHENIX: a comprehensive Python-based system for macromolecular structure solution. Acta Crystallogr D Biol Crystallogr. 2010;66(Pt 2):213–21. doi: 10.1107/S0907444909052925 20124702 PMC2815670

[62] Letunic I, Bork P. Interactive Tree of Life (iTOL) v6: recent updates to the phylogenetic tree display and annotation tool. Nucleic Acids Res. 2024;52(W1):W78–82. doi: 10.1093/nar/gkae268 38613393 PMC11223838

