# Supplementary material for: Broadly conserved protective epitopes on the lyme disease vaccine antigen, OspA
Source: PLoS Pathog. 2026 Apr 21;22(4):e1013740. doi: 10.1371/journal.ppat.1013740 (PMC13138739; doi:10.1371/journal.ppat.1013740)
Supplement: S1 Table — (DOCX) [file ppat.1013740.s001.docx]

| **S1 Table *Borrelia burgdorferi sensu lato s*trains used in this study** | | | |
| --- | --- | --- | --- |
| **Strain** | **OspA ST** | **Native Plasmid Content** | **PMID** |
| *B. burgdorferi* B31-5A4 | ST1 | lp17, lp25, lp21, lp25, lp28-1, lp28-2, lp28-3, lp28-4, lp36, lp38, lp54, lp56  cp9, cp26, cp32-1, cp32-2/7, cp32-3, cp32-4, cp32-6, cp32-8, cp32-9 | 15557639 |
| *B. burgdorferi* HB19 | ST1 | lp5, lp17, lp25, lp28-2, lp28-3, lp28-4, lp28-6, lp36, lp38, lp54, lp56  cp9, cp26, cp32-1, cp32-2/7, cp32-3, cp32-4, cp32-5, cp32-6, cp32-8, cp32-9, cp32-11 | 1339462 |
| *B. burgdorferi* HB19-R1 | N/A (lp54-) | lp17, lp28-2, lp28-6, lp38  cp26, cp32-1, cp32-2/7, cp32-3, cp32-5, cp32-9, cp32-11 | 1339462 |
| *B. afzelii* PKo | ST2 | Uknown | 8432821 |
| *B. garinii* PBr | ST3 | Uknown | 8432821 |
| *B. bavariensis* PBi | ST4 | Uknown | 8432821 |
| *B. garinii* PHei | ST5 | Uknown | 8432821 |
| *B. garinii* TN | ST6 | Uknown | 8432821 |
| *B. garinii* T25 | ST7 | Uknown | 8432821 |
| Abbreviations: ST, serotype; PMID, reference identifier in PubMed ([https://pubmed.ncbi.nlm.nih.gov](https://pubmed.ncbi.nlm.nih.gov/)) | | | |
