# Supplementary material for: Broadly conserved protective epitopes on the lyme disease vaccine antigen, OspA
Source: PLoS Pathog. 2026 Apr 21;22(4):e1013740. doi: 10.1371/journal.ppat.1013740 (PMC13138739; doi:10.1371/journal.ppat.1013740)
Supplement: S2 Table — (DOCX) [file ppat.1013740.s002.docx]

| **S2 Table. Recombinant *B. burgdorferi* strains used in this study** | | | |
| --- | --- | --- | --- |
| **Strain** *^a^* | **mScarlet-I plasmid** | ***ospA* allele derivation** *^b^* | **ST/IST** |
| GGW979 | pGW206 | *B. burgdorferi* B31 | ST1 |
| GGW1072 | pGW206 | N/A (lp54 negative) | - |
| GGW1073 | pGW217 | *B. burgdorferi* B31 | ST1 |
| GGW1074 | pGW218 | *B. afzelli* PKo | ST2 |
| GGW1075 | pGW219 | *B. garinii* PBr | ST3 |
| GGW1076 | pGW220 | *B. bavariensis* PBi | ST4 |
| GGW1078 | pGW222 | *B. garinii* DK29 | ST6 |
| GGW1079 | pGW223 | *B. garinii* T25 | ST7 |
| GGW1081 | pGW224 | *B. speilmanii* PMew | IST13 |
| GGW1098 | pGW225 | *B. bissettiae* DN127 | na |
| GGW1099 | pGW226 | *B. japonica* ATCC 51557 | na |
| GGW1100 | pGW227 | *B. lusitianiae* PoHL-1 | na |
| GGW1101 | pGW228 | *B. mayonii* MN14-1539 | IST14 |
| GGW1102 | pGW229 | *B. turdi* TPT2017 | IST16 |
| GGW1103 | pGW230 | *B. valaisiana* VS116 | IST15 |
| GGW1104 | pGW231 | *B. yangtzensis* DSM24625 | IST17 |
| GGW1123 | pGW239 | *B. americana* S42 | na |
| GGW1124 | pGW240 | *B. finlandensis* SV1 | na |
| GGW1125 | pGW241 | *B. lanei* DSM17992 | na |
| GGW1146 | pGW250 | *B. garinii* PHei | ST5 |
| GGW1163 | pGW253 | *B. andersonii* MOD-5 | na |
| GGW1164 | pGW254 | *B. bavariensis* BgVir | IST9 |
| GGW1165 | pGW255 | *B. bavariensis* FujiP2 | IST10 |
| GGW1166 | pGW256 | *B. carolinensis* SCW-22 | na |
| GGW1168 | pGW258 | *B. kurtenbachi* 25015 | na |
| GGW1169 | pGW259 | *B. tanukii* HK501 | na |
| GGW1170 | pGW260 | *B. maritima* CA690 | na |
| GGW1171 | pGW261 | *B. sinica* CMN3 | na |
| GGW1111 | pGW238 | *B. valaisiana* VS116 G106inserK | IST15 |
| GGW1112 | pGW237 | *B. turdi* TPT2017 T106K | IST16 |
| GGW1116 | pGW232 | *B. burgdorferi* B31 WT | ST1 |
| GGW1117 | pGW233 | *B. burgdorferi* B31 K107T | ST1 |
| GGW1118 | pGW234 | *B. burgdorferi* B31 ∆K107 | ST1 |
| *^a^*, All strains are within the HB19-R1 background except for GGW979, which is in the B31-5A4 background (PMID 38470113); *^b^,* ospA allele (native or mutated) expressed from indicated strain | | | |
