# Supplementary material for: Broadly conserved protective epitopes on the lyme disease vaccine antigen, OspA
Source: PLoS Pathog. 2026 Apr 21;22(4):e1013740. doi: 10.1371/journal.ppat.1013740 (PMC13138739; doi:10.1371/journal.ppat.1013740)
Supplement: S3 Table — (DOCX) [file ppat.1013740.s003.docx]

| **S3 Table, OspA-Fab crystallization solutions** | |
| --- | --- |
| **Fab** | **Crystallization solutions** |
| 221-5 | 100 mM MES pH 6.5, 30% PEG 600, 5% PEG 1000, and 10% glycerol |
| 221-11 | 4% pentaerythritol ethoxylate, 2.2 M ammonium sulfate, 100 mM sodium acetate pH 4.6, 2.0 M Tri-ammonium citrate and 5% MPD |
| 227-1 | 2.0 M Tri-ammonium citrate and 5% MPD |
| 857-2 | 100 mM hepes pH 7.5, 23% PEG 3350, and 200 mM sodium chloride |
