# Supplementary material for: Broadly conserved protective epitopes on the lyme disease vaccine antigen, OspA
Source: PLoS Pathog. 2026 Apr 21;22(4):e1013740. doi: 10.1371/journal.ppat.1013740 (PMC13138739; doi:10.1371/journal.ppat.1013740)
Supplement: S4 Table — (DOCX) [file ppat.1013740.s004.docx]

| **S4 Table. Summary of OspA-Fab structures in this study** | | | | |
| --- | --- | --- | --- | --- |
| **Data Collection** |  |  |  |  |
| OspA-Fab Complex | **221-5** | **221-11** | **227-1** | **857-2** |
| *d*_min_ (Å) | 2.3 | 3.1 | 3.2 | 2.2 |
| No. of reflections | 97352 | 65580 | 30508 | 76590 |
| Average redundancy^a^ | 4.4(3.3) | 15.2(16.1) | 5.7(6.0) | 9.7(9.5) |
| (*I*)/(δ)^a^ | 12.2(1.2) | 9.9(0.8) | 8.9(0.7) | 17.3(1.3) |
| Completeness^a^ (%) | 98.4(96.8) | 99.4(100) | 99.0(100) | 100(100) |
| *R*_merge_*^a,b^* (%) | 13.3(82.8) | 48.9(296.1) | 20.7(167.9) | 25.5(210) |
| *CC^1/2 a,c^* | (0.63) | (0.57) | (0.47) | (0.36) |
| **Refinement** |  |  |  |  |
| Bragg spacings (Å) | 46.9-2.3 | 48.7-3.1 | 48.4-3.2 | 39.9-2.2 |
| Space group | P2_1_2_1_2 | P4_1_22 | I4_1_22 | P2_1_2_1_2_1_ |
| Cell parameters: *a,b,c* (Å) / | 63.8, 90.9, 211.9 | 107.8, 107.8, 316.9 | 109.6, 109.6, 316.2 | 42.8, 124.5, 144.3 |
| *R^d^* / *R*_free_*^e^* (%) | 19.9 / 22.7 | 23.4 / 28.6 | 23.5 / 29.5 | 17.7 / 23.9 |
| No. of reflections | 53698 | 35384 | 16694 | 40676 |
| No. of waters | 230 | 11 | 15 | 284 |
| Rmsd bond length (Å) | 0.005 | 0.002 | 0.002 | 0.007 |
| Rmsd bond angle (°) | 0.77 | 0.589 | 0.569 | 0.947 |
| Ramachandran  favored / allowed*^f^* (%) | 94.4 / 100 | 95.8 / 99.92 | 95.3 / 100 | 95.9 / 100 |
| PDB ID | **8TV3** | **8TVD** | **8TVJ** | **8TUZ** |
| *^a^* Values in outermost shell are given in parentheses.  *^b^ R*_merge_ = (∑│I_i_ - <I_i_>│)/∑│I_i_ │, where I_i_ is the integrated intensity of a given reflection.  *^c^* CC_1/2_= (1+q^2^σ_ε_^2^/<I>2^-1^ , where σ_ε_ denotes the mean error within a half-datase, CC_1/2_ is the correlation coefficient of two split data sets each derived by averaging half of the observations for a given reflection.  *^d^ R* = ∑│\|F*_o_*\| - \|F*_c_*\|│/∑│\|F*_o_*\|│, where F*_o_* and F*_c_* denote observe and calculated structure factors, respectively.  *^e^ R*_free_ was calculated using 5% of data excluded from refinement.  ^f^ Calculated using Molprobity. | | | | |
|  | | | | |
