# Supplementary material for: Broadly conserved protective epitopes on the lyme disease vaccine antigen, OspA
Source: PLoS Pathog. 2026 Apr 21;22(4):e1013740. doi: 10.1371/journal.ppat.1013740 (PMC13138739; doi:10.1371/journal.ppat.1013740)
Supplement: S5 Table — (DOCX) [file ppat.1013740.s005.docx]

| **S5 Table. PCR primers used in this study** | | |
| --- | --- | --- |
| **Primer Name** | **Sequence (5'-3')** | **Description** |
| rrnB_T2_laci_Bb_gibson_F | cagctatgacatgattacgaattcagaaaaataaacaaaagagtttgtagaaacgca | pGW206 construction: Assembly of rrnB_T2 into pGW189 downstream of lacI |
| rrnB_T2_term_R | agaaaaataaacaaaagagtttgtagaaacgca | pGW206 construction: Amplification of rrnB_T2 |
| rrnB_T2_laci_Bb_gibson_R | gcggccagtaataaggatcgagctccgccgggagcggatttgaacgtt | pGW206 construction: Assembly of rrnB_T2 into pGW189 downstream of lacI |
| rrnB_T2_term_F | cgccgggagcggatttgaacgtt | pGW206 construction: Amplification of rrnB_T2 |
| mscarlet_Bb_terminator_gibson_F | CAGGAGGAATGGATGAATTATATAAATAActagcttaattagctgagcttgga | pGW206 construction: Assembly of lambda_T0_rrnB_T1_ into pGW189 downstream of mScarlet-I |
| lambda_rrnB_terminator_F | ctagcttaattagctgagcttgga | pGW206 construction: Incorporation of transcription terminator downstream of lacI in pGW189 |
| mscarlet_Bb_terminator_gibson_R | gtaaaacgacggccagtgccaagcttaagctcctagcggcggatttgtc | pGW206 construction: Assembly of lambda_T0_rrnB_T1_ into pGW189 downstream of mScarlet-I |
| lambda_rrnB_terminator_R | aagctcctagcggcggatttgtc | pGW206 construction: Encorporation of transcription terminator downstream of lacI in pGW189 |
|  | | |
| PospA_pGW163_gibson_F | gcacatccccctttcgccagtactttaaaagacatttaacttttctttttcctgaaagtc | pGW217 construction: Insertion of *ospA_B31_* ORF with silent SphI site after lipidation sequence |
| PospA_F | tactttaaaagacatttaacttttctttttcctg | pGW217 construction: Insertion of *ospA_B31_* ORF with silent SphI site after lipidation sequence |
| ospA_B31_sphI_gibsonR | ggctgctaacattttgcttGCAtgctattaaggctaatattagacctattcccaataaat | pGW217 construction: Insertion of *ospA_B31_* ORF with silent SphI site after lipidation sequence |
| PospA_b31_R | tgctattaaggctaatattagacctattccc | pGW217 construction: Insertion of *ospA_B31_* ORF with silent SphI site after lipidation sequence |
| ospA_ST1_R | ttattttaaagcgtttttaatttcatcaagttttgtaatttcaac | pGW217 construction: Insertion of *ospA_B31_* ORF with silent SphI site after lipidation sequence |
| ospA_B31_pGW206_gibsonR | ttgggaagggcCAGCTGgatttattttaaagcgtttttaatttcatcaagttttgtaatt | pGW217 construction: Insertion of *ospA_B31_* ORF with silent SphI site after lipidation sequence |
|  | | |
| pGW206_pospA_F | gcagcacatccccctttcgccag | ospA ORF sequencing primer |
| pGW206_ospA_R | cgcaactgttgggaagggcgat | ospA ORF sequencing primer |
| ospA_gblock_F | gggaataggtctaatattagccttaatag | ospA ORF sequencing primer |
| Pflgb_scrn_R | cgtctatgcttaagctcttaagttca | ospA ORF sequencing primer |
| ospAB_pGW163_vector_R | tgcgcaactgttgggaagggc | ospA ORF sequencing primer |
