# Supplementary material for: Broadly conserved protective epitopes on the lyme disease vaccine antigen, OspA
Source: PLoS Pathog. 2026 Apr 21;22(4):e1013740. doi: 10.1371/journal.ppat.1013740 (PMC13138739; doi:10.1371/journal.ppat.1013740)
Supplement: S6 Table — (DOCX) [file ppat.1013740.s006.docx]

| **S6 Table. Sequences of *ospA* Gene Fragments Synthesized for Reporter Construction** | | |
| --- | --- | --- |
| **Reporter Plasmid** | **Source of *ospA* Allele** | ***ospA* Sequence** |
| pGW217 | *B. burgdorferi B31 (ST1)* | atgaaaaaatatttattgggaataggtctaatattagccttaatagcaTGCaagcaaaatgttagcagccttgacgagaaaaacagcgtttcagtagatttgcctggtgaaatgaaagttcttgtaagcaaagaaaaaaacaaagacggcaagtacgatctaattgcaacagtagacaagcttgagcttaaaggaacttctgataaaaacaatggatctggagtacttgaaggcgtaaaagctgacaaaagtaaagtaaaattaacaatttctgacgatctaggtcaaaccacacttgaagttttcaaagaagatggcaaaacactagtatcaaaaaaagtaacttccaaagacaagtcatcaacagaagaaaaattcaatgaaaaaggtgaagtatctgaaaaaataataacaagagcagacggaaccagacttgaatacacaggaattaaaagcgatggatctggaaaagctaaagaggttttaaaaggctatgttcttgaaggaactctaactgctgaaaaaacaacattggtggttaaagaaggaactgttactttaagcaaaaatatttcaaaatctggggaagtttcagttgaacttaatgacactgacagtagtgctgctactaaaaaaactgcagcttggaattcaggcacttcaactttaacaattactgtaaacagtaaaaaaactaaagaccttgtgtttacaaaagaaaacacaattacagtacaacaatacgactcaaatggcaccaaattagaggggtcagcagttgaaattacaaaacttgatgaaattaaaaacgctttaaaataa |
| pGW218 | *B. afzelli PKo (ST2)* | aaaaaatatttatgattgggaataggtctaatattagccttaatagcatgcaagcaaaatgttagcagccttgatgaaaaaaacagcgcttcagtagatttgcctggtgagatgaaagttcttgtaagtaaagaaaaagacaaagacggtaagtacagtctaaaggcaacagtagacaagattgagctaaaaggaacttctgataaagacaatggttctggggtgcttgaaggtacaaaagatgacaaaagtaaagcaaaattaacaattgctgacgatctaagtaaaaccacattcgaacttttcaaagaagatggcaaaacattagtgtcaagaaaagtaagttctaaagacaaaacatcaacagatgaaatgttcaatgaaaaaggtgaattgtctgcaaaaaccatgacaagagaaaatggaaccaaacttgaatatacagaaatgaaaagcgatggaaccggaaaagctaaagaagttttaaaaaactttactcttgaaggaaaagtagctaatgataaagtaacattggaagtaaaagaaggaaccgttactttaagtaaggaaattgcaaaatctggagaagtaacagttgctcttaatgacactaacactactcaggctactaaaaaaactggcgcatgggattcaaaaacttctactttaacaattagtgttaacagcaaaaaaactacacaacttgtgtttactaaacaagacacaataactgtacaaaaatacgactccgcaggtaccaatttagaaggcacagcagtcgaaattaaaacacttgatgaacttaaaaacgctttgaaataa |
| pGW219 | *B. garinii PBr (ST3)* | atgaaaaaatatttattgggaataggtctaatattagccttaatagcaTGCaagcaaaatgttagcagccttgatgagaaaaacagcgtttcagtagatttgcctggtggaatgaaagttcttgtaagtaaagaaaaagacaaagacggtaaatacagtctaatggcaacagtagaaaaacttgagctaaaaggaacttctgataaaagcaatggttctggggtacttgaaggtgaaaaagctgacaaaagtaaagcaaaattaacaatttctcaagatttaaatcaaaccacatttgaaattttcaaagaagatggcaaaacattagtgtcaagaaaagtaaattctaaagacaagtcatcaacagaagaaaaatttaatgataaaggtaaattaagtgaaaaagtagtaacaagagcaaacggaactagacttgaatacacagaaataaaaaacgatggatccggaaaagctaaagaagttttaaaaggctttgctcttgaaggaactctaactgatggcggcgaaacaaaattaacagttacagaaggcactgttactttaagcaaaaacatttcaaaatctggagaaataacagttgcacttaatgacactgaaactacccctgctgacaaaaaaactggcgagtggaagtcagatacttctactttaacaattagtaaaaacagtcaaaaaactaaacaacttgtattcacaaaagaaaacacaataacagtacaaaactataacagagcaggcaatgcgcttgaaggcagcccaGCAgaaattaaagatcttgcagagcttaaagccgctttaaaataa |
| pGW220 | *B. bavariensis PBi (ST4)* | atgaaaaaatatttattgggaataggtctaatattagccttaatagcaTGCaagcaaaatgttagcagccttgatgagaaaaacagcgtttcagtagatttacctggtgaaatgaaagttcttgtaagcaaagaaaaagacaaagatggtaaatacagtctaatggcaacagtagacaagctagagcttaaaggaacttctgataaaagcaacggttctggaacacttgaaggtgaaaaatctgacaaaagtaaagcaaaattaacaatttctgaagatctaagtaaaaccacatttgaaattttcaaagaagatggcaaaacattagtatcaaaaaaagtaaattctaaagataagtcatcaatagaagaaaaattcaacgcaaaaggtgaattatctgaaaaaacaatactaagagcaaacggaaccaggcttgaatacacagaaataaaaagcgatggaaccggaaaagctaaagaagttttaaaagactttgctcttgaaggaactctagctgccgacaaaacaacattgaaagttacagaaggcactgttgttttaagcaaacacattccaaactctggagaaataacagttgagcttaatgactctaactctactcaggctactaaaaaaactggaaaatgggattcaaatacttccactttaacaattagtgtgaatagcaaaaaaactaaaaacattgtatttacaaaagaagacacaataacagtacaaaaatacgactcagcaggcaccaatctagaaggcaacgcagtcgaaattaaaacacttgatgaacttaaaaacgctttaaaataa |
| pGW222 | *B. garinii DK29 (ST6)* | atgaaaaaatatttattgggaataggtctaatattagccttaatagcaTGCaagcaaaatgttagcagccttgatgaaaaaaatagcgtttcagtagatttacctggtggaatgacagttcttgtaagtaaagaaaaagacaaagacggtaaatacagtctagaggcaacagtagacaagcttgagcttaaaggaacttctgataaaaacaacggttctggaacacttgaaggtgaaaaaactgacaaaagtaaagtaaaatcaacaattgctgatgacctaagtcaaactaaatttgaaattttcaaagaagatggcaaaacattagtatcaaaaaaagtaacccttaaagacaagtcatcaacagaagaaaaattcaacggaaagggtgaaacatctgaaaaaacaatagtaagagcaaatggaaccagacttgaatacacagacataaaaagcgatggatccggaaaagctaaagaagttttaaaagactttactcttgaaggaactctagctgctgacggcaaaacaacattgaaagttacagaaggcactgttgttttaagcaagaacattttaaaatccggagaaataacaGCAgcacttgatgactctgacactactcgggctactaaaaaaactggaaaatgggattcaaagacttccactttaacaattagtgtgaatagccaaaaaaccaaaaaccttgtattcacaaaagaagacacaataacagtacaaagatacgactcagcaggcaccaatctagaaggcaaagcagtcgaaattacaacacttaaagaacttaaaaacgctttaaaataa |
| pGW223 | *B. garinii T25 (ST7)* | atgaaaaaatatttattgggaataggtctaatattagccttaatagcaTGCaagcaaaatgttagcagccttgatgagaaaaacagcgtttcagtagatttgcctggtgaaatgaaagttcttgtaagtaaagaaaaagacaaagatggtaaatacagtctagaggcaacagtagacaagcttgagcttaaaggaacttctgataaaaacaacggttctggggtgcttgaaggtgtaaaagctgcaaaaagtaaagcaaaattaacaattgctgatgacctaagtcaaactaaatttgaaattttcaaagaagatggcaaaacattagtatcaaaaaaagtaacccttaaagacaagtcatcaacagaagaaaaatttaacgataaaggtaaattaagtgaaaaagtagtaacaagagcaaacggaaccagacttgaatacacagaaatacaaaacgatggatccggaaaagctaaagaagttttaaaaagccttactcttgaaggaactctaactgctgacggcgaaacaaaattaacagttgaagcgggcactgttactttaagcaaaaacatttcagaatctggagaaataacagttgagcttaaggacactgaaactacccctgctgataaaaaatccggaacatgggattcaaagacttctactttaacaattagtaaaaacagtcaaaaaactaaacaacttgtattcacaaaagaaaacacaataacagtacaaaaatacaacacagcaggcactaagcttgaaggcagcccagcagaaattaaagatcttgaagcacttaaagccgctttaaaataa |
| pGW224 | *B. speilmanii PMew (IST13)* | atgaaaaaatatttattgggaataggtctaatattagccttaatagcaTGCaaacaaaatgttagcggccttgacgagaaaaacagcacttcagtagatgtacctggggaacttaaagttcttgtaagcaaagaaaaggacaaagacggtaaatacagcctaatggcaacagtagacaagcttgaactaaaaggaacttctgataaaaatgatggttctggtgtactggaaggcgtaaaagctgacAAGAGCaaagtaAAGttaacaattAGCgaccatttaAGCAAGaccacatttgaagttttcaaaGAGGACggtaaaacaTTGgtgtcaagaaacgtaaatAGCaaagacaaaAGCtcaacaaaagaaAAGttcaatGAGaaaggtgaattgtctGAGAAGacattggtaagagcaaacggaaccaaacttgaatacacaGAGataaaaagcgatggaaccggaaaagctaaagaggttCTTaaagactttactcttgaaggaactctagctaatgaaAAGgcaacattgacagttaaagaaGGTactgttactttaagcaaaaacattgacaaatctggcgaagtaACTgttgcacttaatgacactgacagtactgctgctactaaaaaaactggcgcatgggattcaaaaactAGCactTTGacaattactgttaacagcAAGaaaactAAGGACcttgtatttacaaaacaagacacaataactgtacaaaagtacgactcagcaggaactactttagagggctctgcagtcgaaattaaaacacttgacgaacttaaaaacgctttaaaataa |
| pGW225 | *B. bissettiae DN127* | atgaaaaaatatttattgggaataggtctaatattagccttaatagcaTGCaagcaaaatgttagcggccttgacgagaaaaacagcgtttcagtagatttgcctggtgaaatgaaagttcttgtaagcaaagaaaaagacaaagacggtaagTATagtCTTatggcaacagtagacaagcttgagcttaaaggaacatctgataaaaacaatggatctgggatacttgaaggcgtaaaagctgacaaaagcaaagtaaaattaacagtttctgaggatctcagcacaactacacttgaagttCTTAAGgaagatggcaaaacattggtgtcaAAGaaaacaacttctaaagacaagtcatcaacagaagaaaagttcaatgacaaaggcgaattagctgaaaaaacaatagtaagagcaAATggaaccagacttgaatacacagaagttaaaagcgatggatccggaaaagctaaagaaactttaaaagactatgctcttgaaggaactCTTactgctgaaaaagcaacattggtggttaaagaaggaactgttactttaagtaagcacatttcaaaatccggagaagtaacaGCAgagcttaatgacactgacagtgctcaagctactaaaAAGactggaAAGtgggatGCTggaactAGCactCTTacaattagcgtaaacagcaaaaaaactaaaaaccttgtatttacaaaacaagacacaattacagtacaaaaatacgactcagcaggcACAaacttggaaggcacagcagtcgaaattaaaacacttgatgaacttaaaaacgctttaaaataa |
| pGW226 | *B. japonica ATCC51557* | atgaaaaaatatttattgggaataggtctaatattagccttaatagcaTGCaagcaaaatgttagcaagCTTgatgataaaaacagcacttcagtagacttgcctggtggcatgaaagttcttgtaagcaaagaaAAGgacaagGATggtaaatacaccCTTatggcaacagtaGATaagcttgagCTTaaaggaacttctgatAAGaacaacggttctggaacacttgaaggtgcaAAGactgacAAGagtAAGgtaAAGCTTacaatttctgacgatctaagtaaaaccacacttgaaactCTTaaagaagatggcAAGacaCTTgtatcaagaAAGgtaaactctaaagacatgccatcagaaGAGgaaAAGttcaatgacaaaggtgaattagctgaaaaagtaattCTTagaggtaacggaaccagacttgaaTATacagaaataAAGagcgatggaaccggaaaagctaaagaagttttaAAGaactttactcttgaaggaactctagctaatgacAAGacaacaCTTacagtcAAGgaaggaacagttgctttaaacaagcacattgaaaaatctggaaaaataacagttgatcttaatgatagttcaactactgcttcgactAAGAAGacagcagaatgggatccaAACAACAGCactCTTaccattAGCgttaacagcAAGaaaactacacaacttgtatttacaaagcaagacacaataactatgcaaAAGtacaacacaaatggtgacgctCTTgaaggtgtagcagtagaaattacaacacttgacgcacttAAGaacactCTTAAGtaa |
| pGW227 | *B. lusitianiae PoHL-1* | atgaaaaaatatttattgggaataggtctaatattagccttaatagcaTGCaagcaaaatgctggcgacactgccAGCactgatggcaaaacttcagtagatttacctAGCggagagaaagttcttgtaagcaaagaaAAGAATaaagacggtAAGtacgagCTTatggcaacagtagacAATcttGAGCTTaaagggactAGCgataaaAATgatgggtctggaacacttgaaggcgtaaaagacgacaagAGCaaagttaaattaacagttAGCgatgacttaagtGAGaccaagcttgaaactttaAAGgaagatggtacaccagtgAGCacaaaaacaactAGCaaagacaagAGCgtaacaGAGGAGaaatttgacgacaaaggcgaaCTTactGACAAGataattacaagagcaAATggcaccaagcttGAGttaacaGAGataacaaaagaaggaAAGgcaaaagttaaaGAGactttaaaacacttaacccttgaaggaactCTTgctgacAAGAAGATTacattggcagttAAGGAGGGGactgttactCTTAGCaaagaaattgatGAGaatGAGaaaGTTacagtttcagttaaagatactAGCactactgacgctactaaaAAGactggggcttgggatGAGAACacttccactCTTacaATCactgttAATAGCAAGaaaactaaagacattgtaTTTttagcagatggcacaataactaaacaaAGCtacaacacaAATggggacaagcttgaaggccaagctgaagaagttAAGacacttgatgatctcaaaacagctttaaaataa |
| pGW228 | *B. mayonii MN14-1539 (IST14)* | atgaaaaaatatttattgggaataggtctaatattagccttaatagcaTGCaagcaaaatgttagcagccttgacgagaaaaacagtgtttcagtagatttacctggtgaaattaaagttcttgtaagtaaagaaaaagacaaagacggcaagtacagcctaatggcaacagtagacaagcttgagcttaaaggaacttctgataaaaataatggatctggagtacttgaaggcgtaaaagctgacAAGagtaaagtaAAGCTTacagtttctgacgatctaagcAAGaccacacttgaagttCTTaaagaagatggtAAGacaCTTgtatcaagaAAGgtaacttctAAGgacaagtcaAGCacagaagaaAAGttcaatgaaAAGggcgaattagctgaaAAGacaatgacaagagctgacgaaaccagacttgaatacacagaaattaaaagcgatggatccggaaaagctaaagaagttttaaaaggctatgctcttgaaggaactttaactgccgaaaaaacaacattggtggttaaagaaggaactgttactttaagtaagaacatttcaaaatctggagaagtaacagctgagcttaatgacactgacagtgctgctgctactaaaaaaactggagcttggaattcaggcaccAGCactCTTacaattactgctaacagcAAGaaaactaaagaccttgtgtttacaAAGgaaaacacaattacagtacaaAAGtacgacACTgctggcattAAGttggaaggatcagcagttgaaattAAGacacttgatgaacttAAGaacgctttaAAGtaa |
| pGW229 | *B. turdi TPT2017 (IST16)* | atgaaaaaatatttattgggaataggtctaatattagccttaatagcaTGCaagcaaaatgttagcagccttgatgagaaaaacagcgttAGCgtagatttgcctggtgaaatgAAGgttcttgtaagtaaagaaAAGgacAAGgacggtaaatacagtctaatggcaacaataggcaagcttgaactaaaaggaacttctgatAAGagcaatggttctggggtgcttgaaggtgtaaaagctgacaaaAGCaaagcaAAGCTTacaattgctGAGgacctaggtcaaactAAGtttGAGattttcAAGgaagatggcacaacaCTTatgtcaagaaaagtaacccttaaagacaagtcatcaacagaaGAGaaatttgatgcaaaaggtgctGCTgtaactGAGaaagtaataacaagaaaagacggaaccagacttgaatacacagaaatgAAGagtgatggaagcggaaaagctaaagaagttCTTaaaaactttgcccttgaaggaactttagatactggtggcaaaacaacaTTGacagttAAGcaagacactgttacaCTTacaaaagaaattgataaagatgggaaagtaaaaatctccttagatgatactgcaAGCagttctAAGaaaacaggcgcatgggttgacgctactaatacattaacaatttctgctaacagtAAGaaaactaaagatcttgtgttcacaaaagaaaacacaataacagtacaaagctacgactcaggaggcactactttagaaggcAAGgcagttgaaattacaacacttgaaagccttaaggatgctttaaaataa |
| pGW230 | *B. valaisiana VS116 (IST15)* | atgaaaaaatatttattgggaataggtctaatattagccttaatagcaTGCaagcaaaatgttagcagccttgatgaaaaaaacagcgctAGCgtagatCTTCCTGGAgaaatgaaagttcttgtaagcaaagaaAAGgacAAGgacGGAaaaTATagtCTTgtggcaacagtagacaaagttgagcttaaaggaactAGCgatAAGaacaatggttctggaacacttgaaGGAgtaaaagatgacAAGAGCaaagtaAAGCTTacaatttctgatgatCTTGGAgaaaccaaacttgaaactttcaaagaagatggaacaCTTgtgtcaagaAAGgtaAACttcAAGgacaagtctTTTacagaaGAGaaattcaatGAGaaaGGAgaagtgtctGAGaaaataCTTacaagatcaaacggaactacacttgaatactcacaaatgacagatgctGAGaatgctacaaaagcagtagaaactCTTAAGaatggcattaagcttccaggaaatcttgtaGGAggaAAGacaacaCTTAAGATTacagaaGGAactgttactCTTagcaagcacattgcaaaatctggagaagtaacagttGAGattaacgacacttcaagcactccaaatactAAGAAGactggaAAGtgggatgcaagaAACAGCactCTTacaattattgttgacagcAAGaacaagacaaaacttgtatttacaaaacaagacacaataacagtacaaagctatAATcctgcaggcAATaagCTTgaaGGAacagcagttGAGattaaaacacttcaagaacttAAGaacgctCTTaaataa |
| pGW231 | *B. yangtzensis DSM24625 (IST17)* | atgaaaaaatatttattgggaataggtctaatattagccttaatagcaTGCaagcaaaatgttagcagccttgatgaaAAGaatagcgcttcagtagatttacctggcgaaatgAAGgttcttgtaagcAAGgaaAAGgataaagatggtAAGtacagtctaatggcaacagtagacaaagttgagcttaaaggaacttctgatAAGaacaatggatctggaatgcttgaaggcgtgaaagatgacAAGagtAAGgtaAAGCTTacaattAGCgatgatCTTaacAAGaccacatttGAGactttcAAGgaagatggtAAGacaCTTgtgAGCagaAAGgtaaattccAAGgacaagtcttcaacagtaGAGaaattcaatGAGaaaggtgaattgtccgaaAAGacaataacaagagaaaacggaaccagacttgaatacacagaaataaaaagcgatggaactggaaaagctaaagaagttttaaaagactttactcttgaaggaactctagctgctgataaaacaacattggaagttaaagaaggaacagttactctaagtaagcacattccaaactctggcgaagtaacagttgaaattaatgacacttcaactactcaagctactaagAAGactggaaaatgggatgcaAAGacttcaactCTTacaattgctgttaacaacAAGaatacaAAGagccttgtatttacaAAGGAGgacacaataacagtacaaaactatgactctgcaggcaccaatttagaaggtacagcagttgaaattaaaacacttgatgaacttAAGaacgctttaaaataa |
| pGW238 | *B. valaisiana VS116 (IST15) G106inserK* | atgaaaaaatatttattgggaataggtctaatattagccttaatagcaTGCaagcaaaatgttagcagccttgatgaaaaaaacagcgctAGCgtagatCTTCCTGGAgaaatgaaagttcttgtaagcaaagaaAAGgacAAGgacGGAaaaTATagtCTTgtggcaacagtagacaaagttgagcttaaaggaactAGCgatAAGaacaatggttctggaacacttgaaGGAgtaaaagatgacAAGAGCaaagtaAAGCTTacaatttctgatgatCTTGGAgaaaccaaacttgaaactttcaaagaagatggaAAGacaCTTgtgtcaagaAAGgtaAACttcAAGgacaagtctTTTacagaaGAGaaattcaatGAGaaaGGAgaagtgtctGAGaaaataCTTacaagatcaaacggaactacacttgaatactcacaaatgacagatgctGAGaatgctacaaaagcagtagaaactCTTAAGaatggcattaagcttccaggaaatcttgtaGGAggaAAGacaacaCTTAAGATTacagaaGGAactgttactCTTagcaagcacattgcaaaatctggagaagtaacagttGAGattaacgacacttcaagcactccaaatactAAGAAGactggaAAGtgggatgcaagaAACAGCactCTTacaattattgttgacagcAAGaacaagacaaaacttgtatttacaaaacaagacacaataacagtacaaagctatAATcctgcaggcAATaagCTTgaaGGAacagcagttGAGattaaaacacttcaagaacttAAGaacgctCTTaaataa |
| pGW237 | *B. turdi TPT2017 (IST16) T106K* | atgaaaaaatatttattgggaataggtctaatattagccttaatagcaTGCaagcaaaatgttagcagccttgatgagaaaaacagcgttAGCgtagatttgcctggtgaaatgAAGgttcttgtaagtaaagaaAAGgacAAGgacggtaaatacagtctaatggcaacaataggcaagcttgaactaaaaggaacttctgatAAGagcaatggttctggggtgcttgaaggtgtaaaagctgacaaaAGCaaagcaAAGCTTacaattgctGAGgacctaggtcaaactAAGtttGAGattttcAAGgaagatggcAAGacaCTTatgtcaagaaaagtaacccttaaagacaagtcatcaacagaaGAGaaatttgatgcaaaaggtgctGCTgtaactGAGaaagtaataacaagaaaagacggaaccagacttgaatacacagaaatgAAGagtgatggaagcggaaaagctaaagaagttCTTaaaaactttgcccttgaaggaactttagatactggtggcaaaacaacaTTGacagttAAGcaagacactgttacaCTTacaaaagaaattgataaagatgggaaagtaaaaatctccttagatgatactgcaAGCagttctAAGaaaacaggcgcatgggttgacgctactaatacattaacaatttctgctaacagtAAGaaaactaaagatcttgtgttcacaaaagaaaacacaataacagtacaaagctacgactcaggaggcactactttagaaggcAAGgcagttgaaattacaacacttgaaagccttaaggatgctttaaaataa |
| pGW232 | *B. burgdorferi B31 (ST1) V2* | atgaaaaaatatttattgggaataggtctaatattagccttaatagcaTGCaagcaaaatgttagcagccttgacgagaaaaacagcgtttcagtagatttgcctggtgaaatgaaagttcttgtaagcaaagaaAAGaacaaagacggcaagtacgatctaattgcaacagtagacaagcttgagcttaaaggaacttctgataaaaacaatggatctggagtacttgaaggcgtaaaagctgacAAGagtAAGgtaAAGttaacaatttctgacgatctaggtcaaaccacacttgaagttttcaaagaagatggcAAGacactagtatcaAAGaaagtaacttccaaagacaagtcatcaacagaagaaAAGttcaatgaaAAGggtgaagtatctgaaAAGataataacaagagcagacggaaccagacttgaatacacaggaattAAGagcgatggatctggaAAGgctaaagaggttttaaaaggctatgttcttgaaggaactctaactgctgaaAAGacaacattggtggttAAGgaaggaactgttactttaagcAAGaatatttcaAAGtctggggaagtttcagttgaacttaatgacactgacagtagtgctgctactAAGAAGactgcagcttggaattcaggcacttcaactttaacaattactgtaaacagtAAGAAGactAAGgaccttgtgtttacaAAGGAGaacacaattacagtacaGcaatacgactcaaatggcaccaaattagaggggtcagcagttgaaattacaaaacttgatgaaattaaaaacgctttaaaataa |
| pGW233 | *B. burgdorferi B31 (ST1) K107T* | atgaaaaaatatttattgggaataggtctaatattagccttaatagcaTGCaagcaaaatgttagcagccttgacgagaaaaacagcgtttcagtagatttgcctggtgaaatgaaagttcttgtaagcaaagaaAAGaacaaagacggcaagtacgatctaattgcaacagtagacaagcttgagcttaaaggaacttctgataaaaacaatggatctggagtacttgaaggcgtaaaagctgacAAGagtAAGgtaAAGttaacaatttctgacgatctaggtcaaaccacacttgaagttttcaaagaagatggcACAacactagtatcaAAGaaagtaacttccaaagacaagtcatcaacagaagaaAAGttcaatgaaAAGggtgaagtatctgaaAAGataataacaagagcagacggaaccagacttgaatacacaggaattAAGagcgatggatctggaAAGgctaaagaggttttaaaaggctatgttcttgaaggaactctaactgctgaaAAGacaacattggtggttAAGgaaggaactgttactttaagcAAGaatatttcaAAGtctggggaagtttcagttgaacttaatgacactgacagtagtgctgctactAAGAAGactgcagcttggaattcaggcacttcaactttaacaattactgtaaacagtAAGAAGactAAGgaccttgtgtttacaAAGGAGaacacaattacagtacaGcaatacgactcaaatggcaccaaattagaggggtcagcagttgaaattacaaaacttgatgaaattaaaaacgctttaaaataa |
| pGW234 | *B. burgdorferi B31 (ST1) ∆K107* | atgaaaaaatatttattgggaataggtctaatattagccttaatagcaTGCaagcaaaatgttagcagccttgacgagaaaaacagcgtttcagtagatttgcctggtgaaatgaaagttcttgtaagcaaagaaAAGaacaaagacggcaagtacgatctaattgcaacagtagacaagcttgagcttaaaggaacttctgataaaaacaatggatctggagtacttgaaggcgtaaaagctgacAAGagtAAGgtaAAGttaacaatttctgacgatctaggtcaaaccacacttgaagttttcaaagaagatggcacactagtatcaAAGaaagtaacttccaaagacaagtcatcaacagaagaaAAGttcaatgaaAAGggtgaagtatctgaaAAGataataacaagagcagacggaaccagacttgaatacacaggaattAAGagcgatggatctggaAAGgctaaagaggttttaaaaggctatgttcttgaaggaactctaactgctgaaAAGacaacattggtggttAAGgaaggaactgttactttaagcAAGaatatttcaAAGtctggggaagtttcagttgaacttaatgacactgacagtagtgctgctactAAGAAGactgcagcttggaattcaggcacttcaactttaacaattactgtaaacagtAAGAAGactAAGgaccttgtgtttacaAAGGAGaacacaattacagtacaacaatacgactcaaatggcaccaaattagaggggtcagcagttgaaattacaaaacttgatgaaattaaaaacgctttaaaataa |
| pGW239 | *B. americana S42* | atgaaaaaatatttattgggaataggtctaatattagccttaatagcaTGCaagcaaaatgttagaagccttgacgaaAAGAATagcgtttcagtagatttgcctggtgaaatgaaagttcttgtaagcaaggaaAAGgacaaggacggcaagTATagtctaatggcaacagtagacaagcttgagcttaaaggaacttctgagaaaagcaatggatctggagtacttgaaggcgtaaaagctgacAAGagtaaagtaAAGCTTacaatttctgacgatttaagtcaaaccacacttgaagttCTTAAGGAGgataacacaacactagtatcaAAGAAGgtaacttccaaagacaagtcatcaacagaaGAGaaattcaatGAGaaaggtgaagtatctgaaAAGacaataacaagagcagacggaaccagacttgaaTATacagaaattaaaagcgatggatccggaaaagcaaaagaggttCTTaaaggctatactcttgaaggaacttcaactgctgaaAAGacaacatttatgattAAGgaaggaactgttactCTTagcaagaatatttcaAAGtctggggaagtttcagttgaacttaatgacactgacagcactgctggtactAAGaaaactggagcttggaattcaggcacttcaactttaacaattactgtaAATagcAAGaaaactAAGgacCTTgtgtttacaAAGgaaaacacaattacagtacaaAAGtacgactcaaatggcaccAAGttagaggggtcaGCTgttgaactcAAGacacttgatgaaattaaagcagctttaaaataa |
| pGW240 | *B. finlandensis SV1* | atgaaaaaatatttattgggaataggtctaatattagccttaatagcaTGCaagcaaaatgttagcAGTcttgacgagAAGaacTCTgtttcagtagatttgcctggtgaaataAAGgttcttgtaagcaaaGAGAAGaacaaagacggtaagtacagtctaatggcaacagtagacaagcttgagcttaaaggaacttctgatAAGaacaatggatctggggtacttgaaggtgtaAAGgctgacAAGagtaaagtaAAGttaacagtttctgacgatctaggccaaactacacttgaagttTTGaaagaagatggtAAGacattagtatcaagaAAGgtaacttccaaagacaagtcatcaacagaaGAGaaattcaacGAGaaaggtgaattagctgaaAAGataatgacaagagcaaacggaacaagacttgaatacacagaaattaaaagcgatggatccggaaaagctaaagaagttttaaaagactatgttcttgaaggaactctaactgctgaaAAGacaacattagtggttaaagagggaacagttactttaagcaagcacatttcaaaatctggagaagtaacagctgagcttaatgacactgaaagcagttctgctactAAGaaaactgcagcttggaattcaggtacttcaactTTGacaattactgtaaacagtAAGAAGactaaagaccttgtgtttacaAAGGAGaacacaattacagtacaaAAGtacgacacagctggaaccaacctagaaggatcagcagttgaaattAAGaaacttgatgaacttAAGaacgctttaaaataa |
| pGW241 | *B. lanei DSM17992* | atgaaaaaatatttattgggaataggtctaatattagccttaatagcaTGCaagcaaaatgttagcggccttgacgagAAGaacagcgtttcagtagatttacctggtgaaatgaaagttcttgtaagcaaaGAGaaagacaaagacggcaagtacagtctaatggcaacagtagacaagcttgagcttaaaggaacttccgatAAGaacaatggatctggggtacttgaaggtGTTaaagctgacAAGagtaaagtaAAGttaacagtttctgacgatctaagtcaaaccacacttgaaGTACTTaaaGAGgatggtAAGacattagtgtcaagaAAGgtaacttctAAGgacaagtcaAAGacagaaGAGaaattcaatGAGaaaggtgaaTTGtctgaaAAGataatgacaagagcaaacggaacaagacttgaatacacagaaattAAGagcgatggatccggaaaagctaaagaagttttaaaagactatactcttgaaggaactctagctgctgaaAAGacaacattggtggttaaagaaggaactgttACAttaagtAAGaacatttcaaaatctggagaagtaacagttgagcttaacgatactgacagtactcctgctactAAGaaaactggagcttggaatAGCggtacttcaACATTGacaattactGTTaacagcAAGaaaactAAGgatTTGgtgtttACTAAGGAGaacacaattacagtacaaAAGTATGACggagctggcactaaattggaaggatcagcagttgaaattaaaacacttaatgaacttAAGaacgctttaaaataa |
| pGW250 | *B. garinii PHei (ST5)* | atgaaaaaatatttattgggaataggtctaatattagccttaatagcaTGCaagcaaaatgttagcagccttgatgaaAAGaatagcgtttcagtagatCTTcctggtggaatgaaagttcttgtaagtaaagaaAAGgacaaagatggtaaatacagtctaatggcaacagtaGAGaagcttgagcttaaaggaactAGCgatAAGaacaacggttctggaacacttgaaggtgaaAAGactgacAAGAGCaaagtaAAGttaacaattgctgaggatctaagtAAGaccacatttgaaatcttcaaagaagatggcaaaacaCTTgtatcgAAGaaagtaacccttaaagacaagtcatcaacagaaGAGaaattcaacgaaaagggtgaaatatctgaaAAGacaatagtaagagcaaatggaaccagacttgaatacacagacataAAGagcgataaaaccggaaaagctaaagaagttttaaaagactttactcttgaaggaactctagctgctgacggcaaaacaacattgaaagttacagagggcactgttactCTTagcaagaacatttcaaaatccggagaaataacagttgcacttgatgacactgactctagcggcaatAAGaaatccggaacatgggattcaggtacttctactCTTacaattagtAAGaacagaacaaaaactaaacaacttgtattcacaAAGgaagacacaataacagtacaaaactacgactcagcaggcaccaatctagaaggcaaagcagtcgaaattacaacacttaaagaacttaaaaacgctttaaaatag |
| pGW253 | *B. andersonii MOD-5* | atgaaaaaatatttattgggaataggtctaatattagccttaatagcaTGCaagcaaaatgttagcagccttgacgagaaaaacagcgtttcagtagatgtacctggtggaatgaaagttcttgtaagcaaagaaaaaaacaaagacggcaagtacgatctaatggcaacagtggacaagcttgagcttaaaggaacttctgacaaaaacaatggatctggagtacttgaaggcgtaaaagctgataaaagtaaagtaAAGttaacagttgctgacgatctaagcAAGaccacacttgaagttttaAAGgaagatggtAAGacattagtgtcaagaAAGgtaacttccaaggacaagtcaacaacagaagaaAAGttcaacgaaAAGggtgaattgtctgaaaaaacaatgacaagagcaaacggaaccaaacttgaatacacagaaattaaaagcgatggatccggaaaagctaaagaagttttaaaaggctatgttcttgaaggaactctatctactgacaaagcaacattggtggttaaagaaggaacagttactttaagtaagcacatttcaaaatccggagaagtaacagctgatcttaatgacactgacagcactcctgctactaaaAAGactggaaattggaattcaagtacttcaactCTTacaattactgtaaacagtAAGAAGactAAGgaccttgtgtttacaAAGgaaaacacaattacagtacaaAAGtacaactcagctggcactagcttggaaggatcagcagctgaaattaaaacactcgatgaacttaaaaccgctttaaaataa |
| pGW254 | *B. bavariensis BgVir (IST9)* | atgaaaaaatatttattgggaataggtctaatattagccttaatagcaTGCaagcaaaatgttagcagccttgatgagaaaaatagcgtttcagtagatttacctggtgaaatgaaagttcttgtaagcaaagaaaaagacaaagatggtaaatacagcctaatggcaacagtagacaaacttgagctaAAGggaacttctgataaaagcaatggttctgggatacttgaaggtgtaAAGactgatAAGagtAAGgcaAAGCTTaccatttctgacgatctaagcAAGaccacatttgaagttttcAAGgaagatggtAAGacaCTTgtgtcaagaAAGgtaaattctAAGgacaagtcatcaacagaagaaAAGtttaatgcaAAGggtgaaCTTagtgaaAAGgtagtaacaagagcaaatggaaacagacttgaatacacagaaataAAGagcgatggatccggaAAGgctAAGgaagttCTTAAGgactttactcttgaaggaactctaactgctgacAAGacaacattaacaatacaagagggcagtgttactCTTagcaagaacattgcaaagtctggagaaataacagttgaacttaatgacactgactctagcggtgatAAGaaatctggacaatggaattcaagtacttctactCTTacaattagtgctAAGagcAAGAAGactAAGgatcttgtatttacaAAGcaagacacaataacagtacaaAAGtacgactcagcaggaactaatcttgaaggctccgcagttgaaattAAGacacttgacgaacttAAGaacgctttaaaataa |
| pGW255 | *B. bavariensis FujiP2 (IST10)* | atgaaaaaatatttattgggaataggtctaatattagccttaatagcaTGCaagcaaaatgttagcagccttgatgagaaaaacagcgtttcagtagatttacctggtgaaatgaaagttcttgtaagcaaagaaaaagacaaagacggtaaatacagcctaatggcaacagtagacaagcttgagcttaaaggaacttctgataaaaacaacggttctggaacacttgaaggtgaaaaaaccgacaaaagtaaagcaaaattaaccatttctgacgatctaagcaaaaccacatttgaagttttcaaagaagatggcAAGacaCTTgtgtcaagaAAGgtaaattctAAGgacaagtcatcaacagaagaaAAGtttaatgcaAAGggtgaattaagtgaaAAGgtagtaacaagagcaaatggaaacagacttgaatacacagaaatgAAGagcgatggatccggaAAGgctAAGgaagttCTTaaagactttgctcttgaaggaactctaactgctgacggcAAGacaacgctaacaatacaagagggcactgttactCTTAAGAAGgaaattgaaAAGgctggaacagtaAAGctctttttagatgacacttcaagtggtagtactAAGaaaacagctgtatggagcgatacttctaacaccCTTacagttagtgctgacagcAAGaaaatcaaagatttcgtgttcttaacagacggtacaattacagtacaaaattacgacAAGgcaggaaccaagcttgaaggtacagcaaccgaaattaaagatcttacagcacttaaaacagctttaaaataa |
| pGW256 | *B. carolinensis SCW-22* | atgaaaaaatatttattgggaataggtctaatattagccttaatagcaTGCaagcaaaatgttagcagccttgacgagaaaaacagcgtttcagtagatttacctggtgaaatgaaagttcttgtaagtaaagaaaaagacaaagacggtaagtacagtctaatggcaacagtagacaatcttgagcttaaaggaacttctgagaaaaacaatggatctggggtacttgaaggcgtaaaagctgacaaaagcAAGgtaAAGttaacagtttctgaagatctcagcacaactacacttgaagttCTTaaagaagatggcAAGacattcgtgtcaAAGaaaacaacttctAAGgacaagtcatcaacagaagaaaagttcaatgacaaaggcgaattatctgaaaaaataatggtaagagcaaacggaaccaaacttgaatacacagaaattaaaaacgatggatccggaAAGgctAAGgaaactttaAAGgaatatgttcttgaaggaactctaactgctgaaAAGgcaacattgacagttAAGcaaggaactgttactCTTagtaaacacatttcaaaatccggagaagtaacagctgagcttaatgacactgacagcgctcaagctactaaaaaaactggaaaatgggatgcaggaacttcaaccttaacaattagcgtaaacagcaaaaaaaccaaagaccttgtatttacaaaacaagacacaattacagtacaaaaatacgactcagcaaacaccaacttggaaggtacagcagtcgaaattaaaacacttgatgaacttaaaaacgctttaaaataa |
| pGW258 | *B. kurtenbachii 25015* | atgaaaaaatatttattgggaataggtctaatattagccttaatagcaTGCaagcaaaatgttagcagccttgacgagaaaaacagcgtttcagtagatttgcctggtgaaatgaaagttcttgtaagcaaagaaaaagacaaagacggcaagtacagtctaatggcaacagtagacaagcttgagcttaaaggaacatctgataaaaacaatggatctggggtgcttgaaggcgtaaaagctgacaaaagcaaagtaaaattaacagtttctgacgatctaagcacaaccacacttgaagttttaaaagaagatggcAAGacattagtgtcaAAGAAGacaacttctAAGgataagtcatcaacagaagaaaagttcaatgaaAAGggcgaattagttgaaAAGataatggcaagagcaaacggaaccatacttgaatacacagaaattaaaagcgatggatccggaaaagctaaagaaactttaaaagaatatgttcttgaaggaactctaactgctgaaAAGgcaacattggtggttAAGgaaggaactgttactttaagtaagcacatttcaaaatctggagaagtaacagctgaacttaatgacactgacagtactcaagctactaaaaaaactgggaaatgggatgcaggcacttcaactttaacaattactgtaaacaacAAGAAGactAAGgcccttgtatttacaAAGcaagacacaattacatcacaaAAGtacgactcagcaggaaccaacttggaaggcacagcagtcgaaattaaaacacttgatgaacttaaaaacgctttaagataa |
| pGW259 | *B. tanukii HK501* | atgaaaaaatatttattgggaataggtctaatattagccttaatagcaTGCaagcaaaatgttagcagccttgacgaaaagaacagcgattcagttgatctacccggcgaaatgaaagttcttgtaagcaaagaaaaagataaagacggtaaatacagtctaacagcaacagtagacaaaattgagctgaaaggaacttctgataaaaacaatggatctggggtacttgaaggcttaaaagctgacaaaaatAAGgtaAAGCTTacaatttctgacgatctaagtAAGaccacatttgaaattttcAAGgaagatggcAAGacaCTTgtgttaAAGagagtaaattctAAGgacaagtcatcaacagaaGAGAAGttcaatgaaAAGggtgaattgtatgaaAAGatactaacaagagaaaacggaaccagacttgaatacacagaaataaaaagcgatggaaccggaaaagccaaagaagttCTTaaagactttactcttgaaggaactctagctgctgaaaaagcaacattgatggttaaagaaggaactgttactttaagtaagaacattgcaaaatctggagaagtaaccgttgagattgctgacactaacagtgatgcttctactAAGAAGtctggaAAGtgggattcaaatacttcaactCTTacaattgctattaacagcAAGaacaagAAGaaccttgtatttacaAAGcaagacacaataacagtacaaaactatgactctgcgggcaccaagctagaaggtacagcagttgaaattaaaacacttgatgagcttaaaaacgctttaaaataa |
| pGW260 | *B. maritima CA690* | atgaaaaaatatttattgggaataggtctaatattagccttaatagcaTGCaagcaaaatgttagcagccttgatgaaaaaaacagcgtttcagtaggattgcctggtgaaatgaaagttcttgtaagcaaagaaaaagacaaggacggtaagtacagtctaatggcaacagtagacaagcttgaacttaaaggaacttctgataaaaacaatggttctggagtacttgaaggcgaaaaagctgacAAGagtaaagtaAAGttaacagttgctgaagatctaagcAAGaccacacttgaagttCTTAAGgaagatggtAAGacattagtatcaagaAAGgtaacttccAAGgacaagtcatcaacagaagaaAAGttcaacgaaaaaggcgaaCTTtctgaaAAGacaataacaagagcagacggaacaaggcttgaatacacagaaatcAAGagcgatggaaacggaAAGgctAAGgaagttCTTAAGggctatgctcttgaaggaactCTTactgctgaaAAGacaACTCTTgaagttAAGgaaggaacagttaccctaagtaagaacatttcaAAGtctggagaagtaACCgttgcgcttaatgacactgcatctggtattgctAAGAAGacagctacatgggaaagtagtactaacaccCTTacaattagtgctaacagtAAGAAGactcaagacattgtctttacaAAGgaaaacacaattacagtacaaAAGtatgactcaggtggcaccAAGCTTgaaggtaaagcagttgaaattAAGacacttgaccaactcAAGaccgctttaaaataa |
