# Supplementary figures and images for: Broadly conserved protective epitopes on the lyme disease vaccine antigen, OspA
Source: PLoS Pathog. 2026 Apr 21;22(4):e1013740. doi: 10.1371/journal.ppat.1013740 (PMC13138739; doi:10.1371/journal.ppat.1013740)

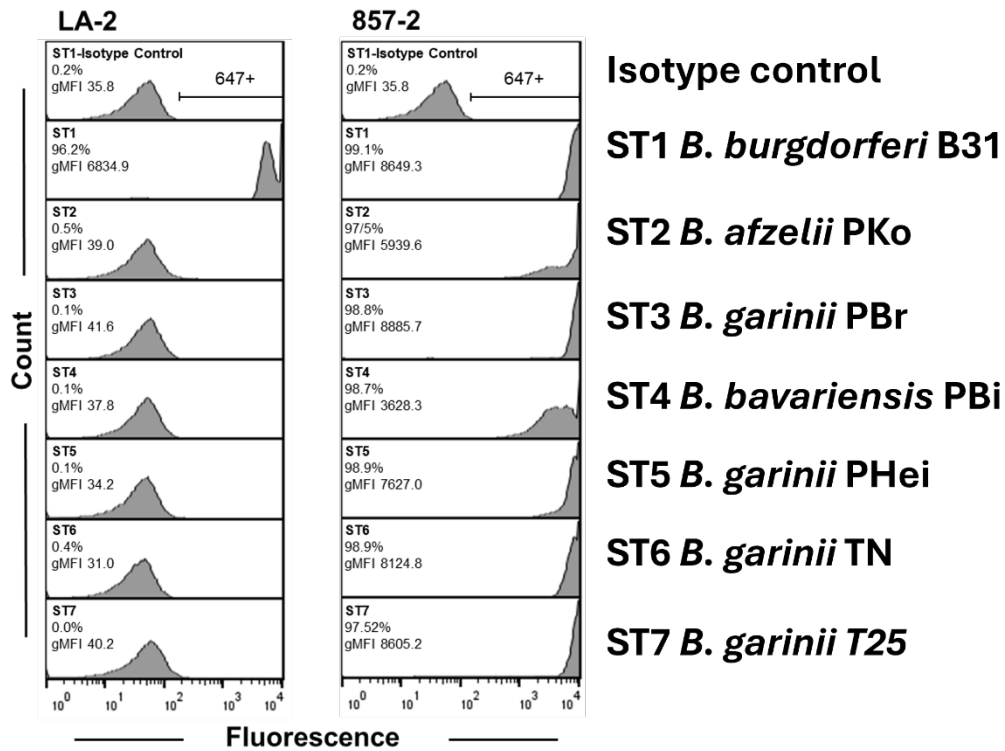

**S1 Fig. 857-2 binds to OspA serotypes 1-7 expressed on the surface of *Borrelia* primary isolates.**

Supplement: S1 Fig — Representative flow cytometric analysis of live Borrelia primary isolates expressing OspA serotypes (ST) 1–7 with 10 µg/ml of Bin 1 IgG mAb 857-2, followed by addition of Alexa 647-labeled goat anti-human IgG secondary antibody. An isotype control, O5 antigen-specific IgG variant Sal4, was run with each ST, however ST 1 is shown as the representative. OspA ST 1-specific IgG mAb LA-2 was run with ST 1–7 for comparison. The horizontal bracket represents the region on subsequent plots that are positive for fluorescence labeling (647+). The percentage of events positive for Alexa 647 fluorescence labeling and the geometric mean fluorescence intensity (gMFI) are shown. Compared to isotype controls, gMFI values of LA-2 bound to ST1, and 857-2 bound to ST1–7 are significantly different (p < 0.0001). There is no significant difference in gMFI with LA-2 and ST2–7 strains (p > 0.9999). N = 3. Statistical comparisons were performed using two-way ANOVA with Dunnet’s multiple-comparison test. Image was generated in FlowJo. ST 1-B. burgdorferi B31; ST 2- B. afzelii Pko; ST 3- B. garinii PBr; ST 4- B. bavariensis PBi; ST 5-B. garinii PHei; ST 6- B. garinii TN; ST 7- B. garinii T25. (PDF) [file ppat.1013740.s007.pdf]

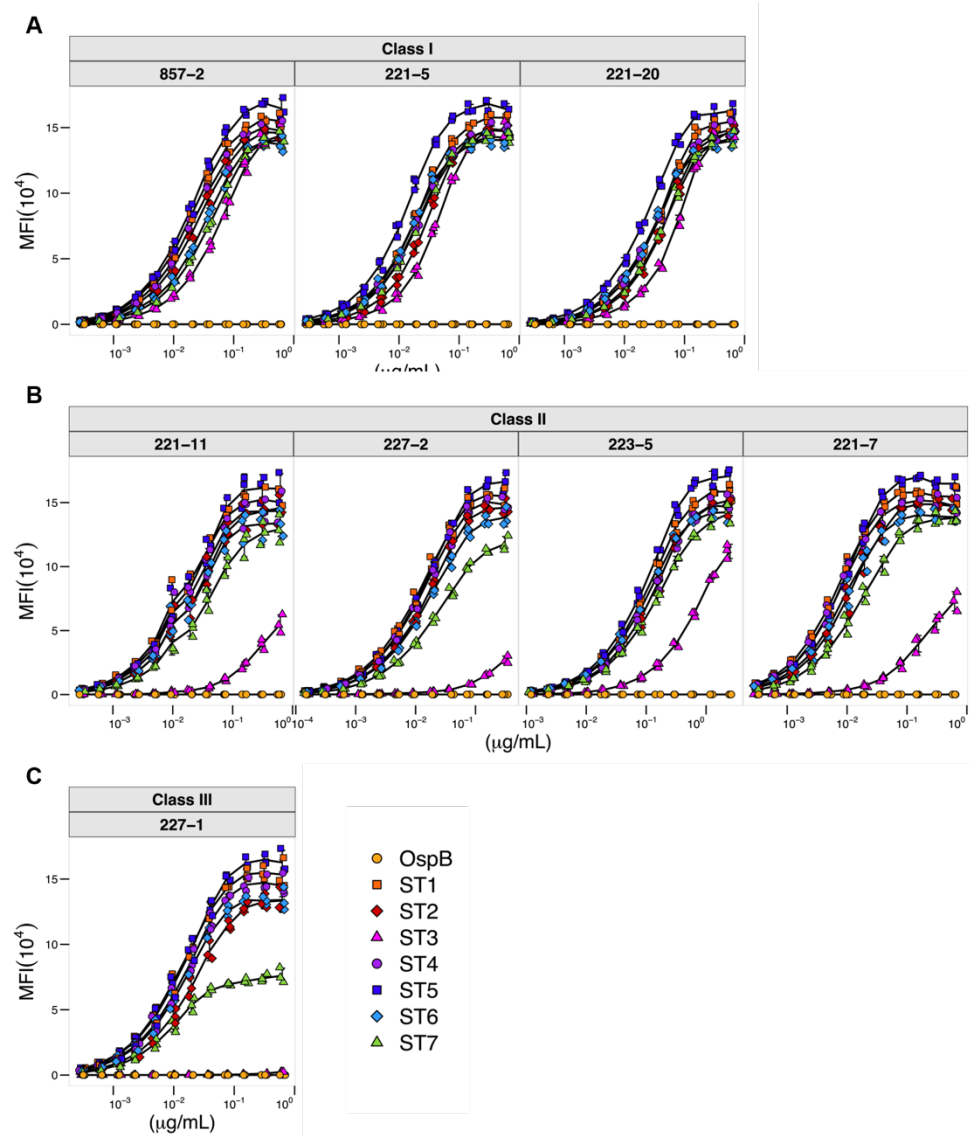

**S2 Fig. Binding of Bin 1 mAbs to recombinant OspA ST1-7.**

Supplement: S2 Fig — Bin 1 IgG mAbs were subjected to MIA using non-lipidated recombinant ST 1–7 coupled microspheres as detailed in the materials and methods. Recombinant OspB was included as a negative control. Median fluorescent intensity (MFI) is displayed on the y-axis and mAb concentration is shown on the x-axis. The plotted lines show the mean MFI and standard deviation for each serotype across 3 technical replicates with each point representing a single experiment. The concentrations shown are two-fold serial dilutions with starting concentrations of 2.5 µg/mL (223-5), 0.625 µg/mL (857-2, 221-5, 221-20, 221-11, 221-7, 227-1), or 0.3125 µg/mL (227-2). (PDF) [file ppat.1013740.s008.pdf]

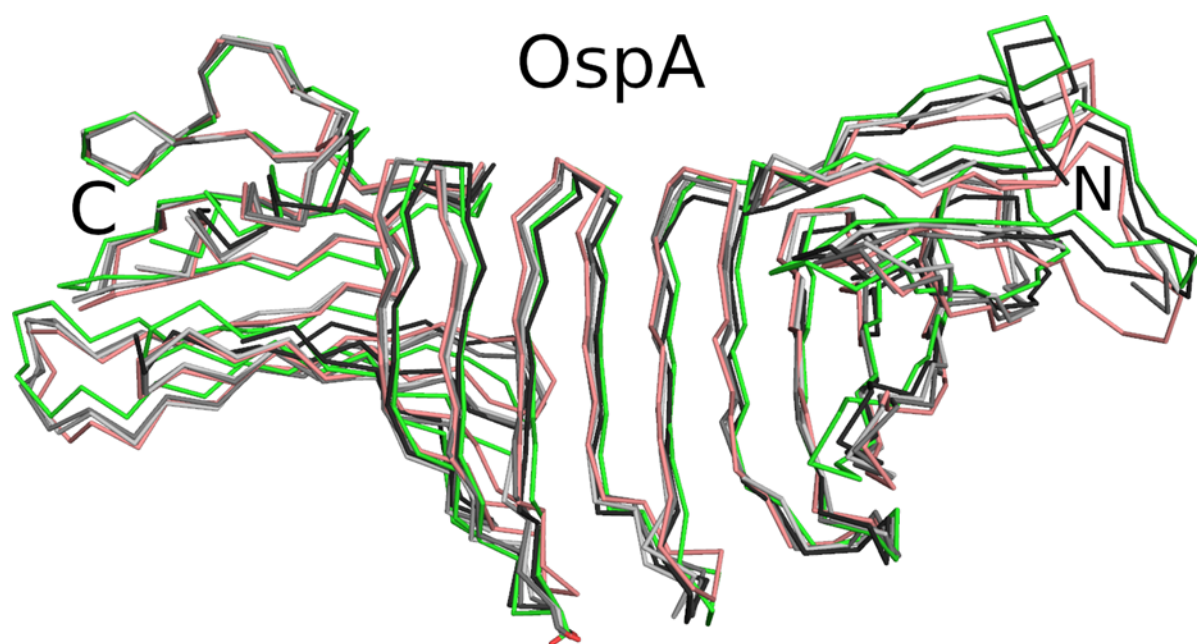

S4 Fig. Structural similarity of OspA.

Supplement: S4 Fig — Superpositioned Cα-traces of the unliganded form of OspA (PDB ID: 2G8C) colored green with OspA form all four Fab-OspA structures (shaded from light to dark gray) depicting the structural similarity of OspA across these different structures. (PDF) [file ppat.1013740.s010.pdf]

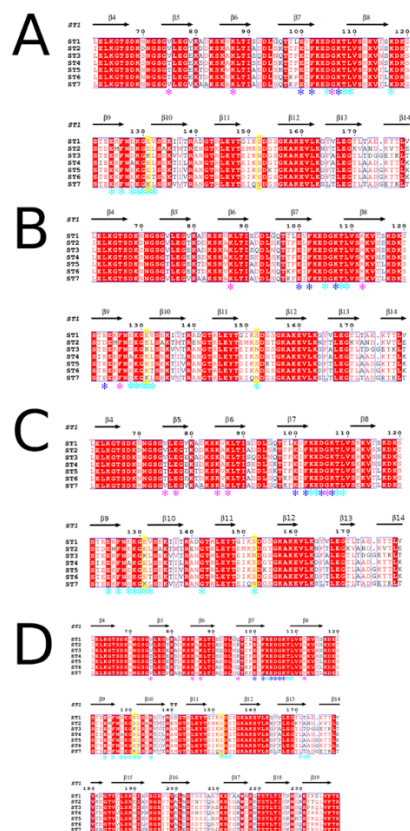

**S5 Fig. OspA sequence alignments.**

Supplement: S5 Fig — The high sequence similarity between OspA residues in B. burgdorferi (ST1) that interact with Fabs 857-2, 221-5, 221-11, 227-1 and the OspA sequence conservation from serotypes 2–7, supports our observed cross-reactivity of mAbs 857-2, 221-5, 221-11, 227-1 across nearly all seven serotypes. Sequence alignment of the interacting regions of OspA from B. burgdorferi (ST1) with (A) 857-2 (B) 221-5 (C) 221-11 (D) 227-1 aligned with OspA sequences ST2-ST7. The yellow boxes in all four panels highlight residue positions 131 and 152, which mark primary sequence differences among OspA serotypes that influence antibody cross-reactivity. In ST 3 and 7, residue 131 is a lysine (Lys-131) rather than a glutamate (Glu-131), and residue 152 is an asparagine rather than a serine. These substitutions appear to underlie the reduced cross-reactivity of Fabs 221-11 and 227-1 with ST3 and ST7. Magenta asterisks depict VL interacting residues, cyan asterisks depict VH interacting residues, and blue asterisks denote interaction with VL and VH residues. Secondary structural elements (β-strands 4–14 in panels A,B,C and β-strands 4–19 in panels D) are drawn as black arrows above the sequence and labelled accordingly. Red colored sequence denotes sequence identity and white colored sequence shows reduced sequence similarity. Figure made with ClustalW and ESPript 3.0. (PDF) [file ppat.1013740.s011.pdf]

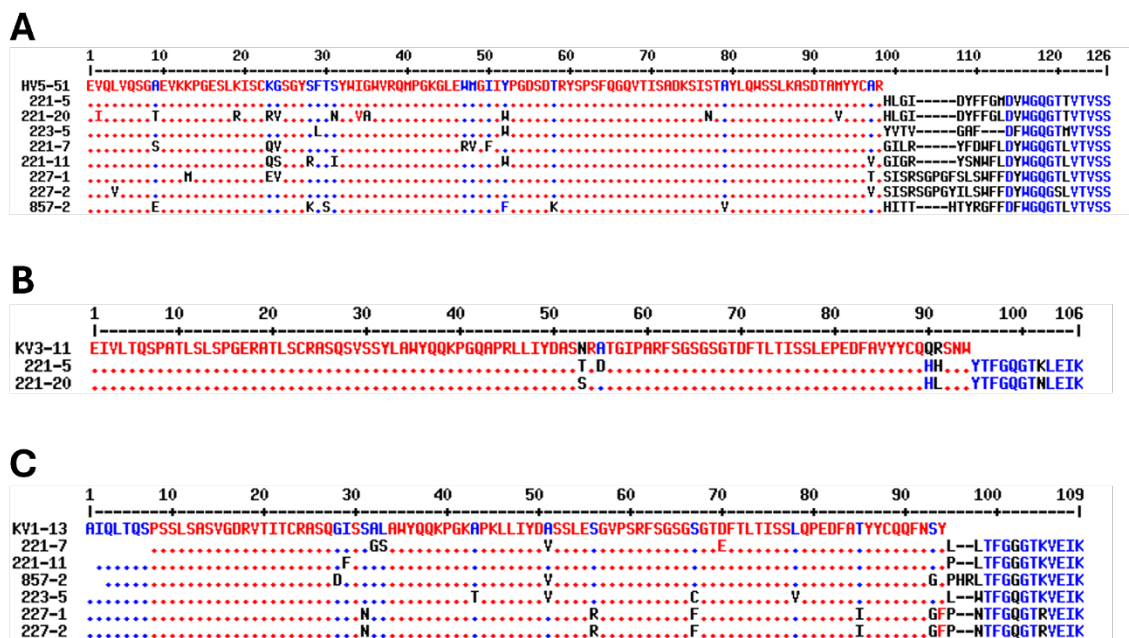

**S6 Fig. Inferred germline usage.**

Supplement: S6 Fig — (A) Heavy chains of the 8 Bin 1 mAbs aligned to the HV5–51*01 Germline Gene. (B) Light chains of 221-5 and 221-20 aligned to KV3–11*01 Germline Gene. (C) Light chains of the other six mAbs aligned to KV1–13*02 Germline Gene. (PDF) [file ppat.1013740.s012.pdf]

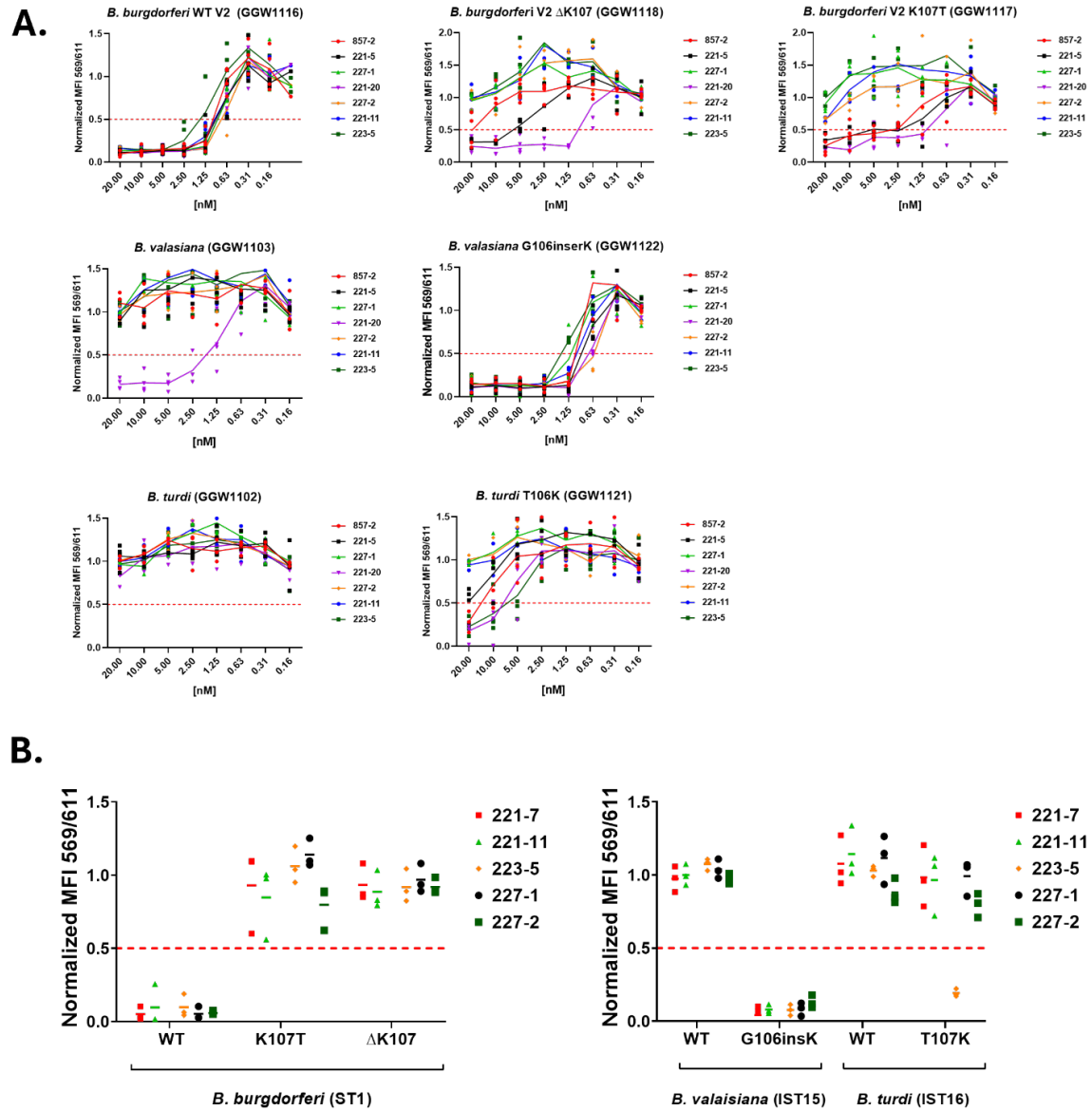

Supplement: S9 Fig — (A) Antibody titration curves summarizing complement-dependent killing of HB19-R1 viability reporter strains expressing OspA variants with polymorphisms at position 107. Complement-dependent killing assays were performed as described in the materials and methods section using anti-OspA Bin1 mAbs and B. burgdorferi HB19-R1 viability reporter strains expressing “WT” OspA variants from B. burgdorferi B31 (ST1), B. valaisiana VS116 (IST15), and B. turdi TPT2017 (IST16), or mutated derivatives containing deletions, insertions, or substitutions at residue 107. (B) Susceptibility of HB19-R1 reporter strains harboring OspA variants with polymorphisms at position 107 to complement dependent killing mediated by 66.7 nM of Class II & Class III Bin 1 mAbs. All data shown includes at least three independent experiments per strain with data normalized as described. (PDF) [file ppat.1013740.s015.pdf]

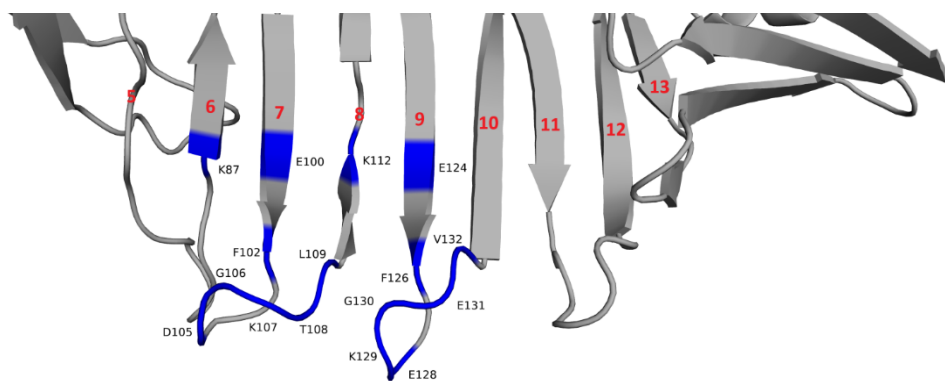

**S10 Fig. OspA  $\beta$ -Strand Labels, and Combined Epitope of Bin 1 mAbs.**

Supplement: S10 Fig — Ribbon diagram of OspA depicting close up of the CBS with residues conserved within the epitopes of 857-2, 221-5, 221-11, and 227-1 labeled and shaded in blue. Strand numbers are labeled in red. (PDF) [file ppat.1013740.s016.pdf]
