# Supplementary material for: Broadly conserved protective epitopes on the lyme disease vaccine antigen, OspA
Source: PLoS Pathog. 2026 Apr 21;22(4):e1013740. doi: 10.1371/journal.ppat.1013740 (PMC13138739; doi:10.1371/journal.ppat.1013740)
Supplement: S3 Fig — (A) Antibody titration curves depicting bactericidal activity against B. burgdorferi HB19-R1 reporter strains expressing OspAST1–7. Complement-dependent bactericidal assays were performed with anti-OspAST1 Bin1 mAbs and B. burgdorferi HB19-R1 strains harboring an IPTG-inducible mscarlet-I viability reporter plasmid expressing ospA serotypes 1–7 as described in the material and methods. Experimental controls included an HB19-R1 strain carrying the IPTG-inducible viability reporter plasmid without an ospA variant and a mAb with bactericidal activity restricted to OspAST1 (LA-2). The data shown encompasses 3–5 independent experiments per strain with data normalized as described within the materials and methods section. (B) Heat map summarizing statistical comparison of differences in susceptibility to anti-OspA ST1 mAbs between the HB19-R1 OspAST1 reporter strain and reporter strains expressing OspAST2–7. Statistical analyses were performed via one-way ANOVA followed by Dunnett’s multiple-comparison test. Significant differences in susceptibility are denoted by purple or pink shading. (PDF) [file ppat.1013740.s009.pdf]

A.

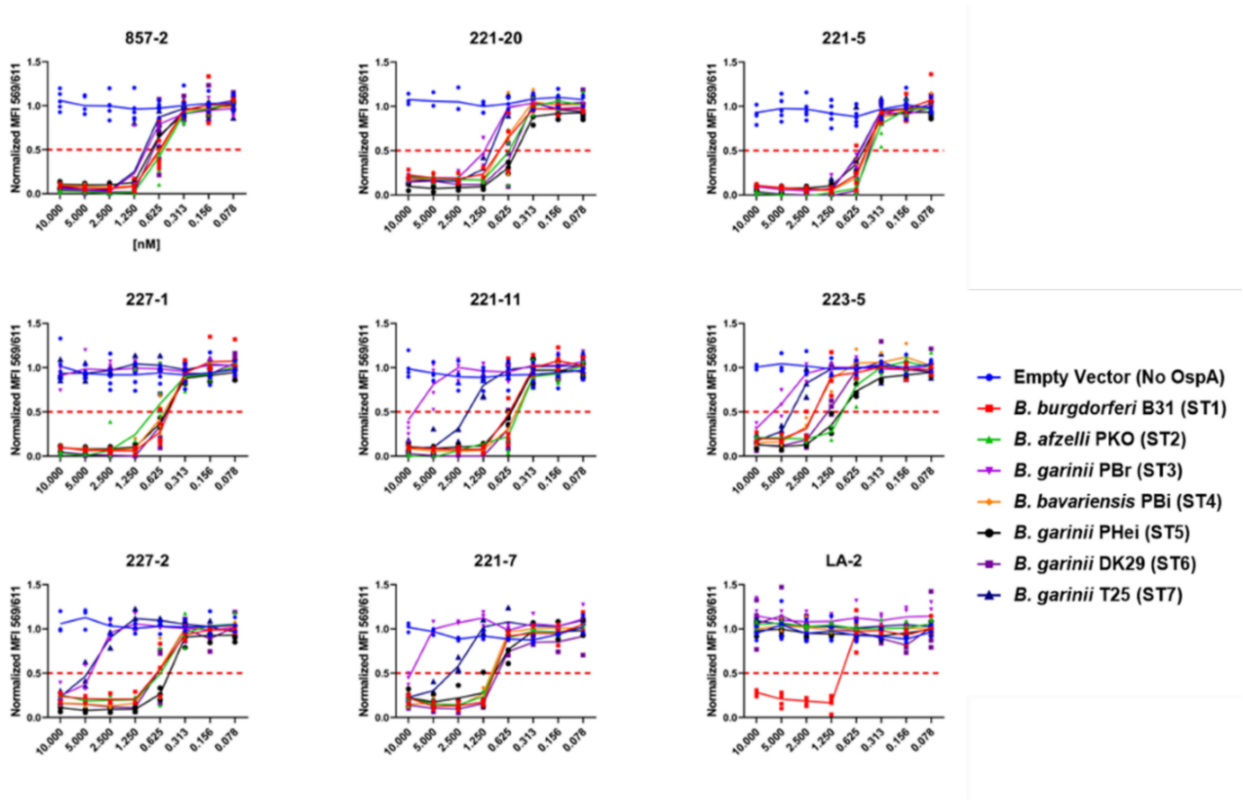

B.

	Class I			Class II				Class III	
	857-2	221-5	221-20	221-11	227-2	223-5	221-7	227-1	LA-2
Empty Vector (No OspA)	<0.0001	<0.0001	<0.0001	<0.0001	<0.0001	<0.0001	<0.0001	<0.0001	<0.0001
B. afzelli PKo (ST2)	0.9947	0.7903	>0.9999	>0.9999	>0.9999	0.1935	>0.9999	0.1710	<0.0001
B. garinii PBr (ST3)	0.0771	>0.9999	0.2142	<0.0001	0.0014	0.0005	<0.0001	<0.0001	<0.0001
B. bavariensis PBi (ST4)	>0.9999	0.7903	>0.9999	>0.9999	>0.9999	0.9123	>0.9999	0.9927	<0.0001
B. garinii PHei (ST5)	0.6380	0.8764	0.5866	>0.9999	0.9994	0.1935	0.2608	0.9969	<0.0001
B. garinii DK29 (ST6)	0.9947	0.7903	0.9660	>0.9999	>0.9999	0.6571	>0.9999	0.9927	<0.0001
B. garinii T25 (ST7)	0.0771	>0.9999	0.9660	0.2697	0.0014	0.3827	<0.0001	<0.0001	<0.0001

Significance
<0.0001
<0.001
<0.01
<0.05
>0.05

S3 Fig. Anti-OspA$_{ST1}$ Bin1 mAbs promote complement-dependent killing of recombinant *B. burgdorferi* Strains expressing OspA ST1-7.
