# Supplementary material for: Broadly conserved protective epitopes on the lyme disease vaccine antigen, OspA
Source: PLoS Pathog. 2026 Apr 21;22(4):e1013740. doi: 10.1371/journal.ppat.1013740 (PMC13138739; doi:10.1371/journal.ppat.1013740)
Supplement: S7 Fig — Representative sequences from each OspA clade were aligned via MAFFT using OspA from B. burgdorferi strain B31 as a reference. Bolded strain names indicate OspA variants expressed by HB19-R1 viability reporter strains and subjected to complement-dependent bactericidal assays with anti-OspAST1 Bin 1 mAbs. Amino acid residues numbered in white font denote conservation within the epitopes of two or more Bin1 mAbs (857-2, 221-5, 221-11, 221-7, and 227-1). Numbered amino acid residues with colored background form critical interactions with residues within the paratopes of Bin1 mAbs with solved crystal structures. (PDF) [file ppat.1013740.s013.pdf]

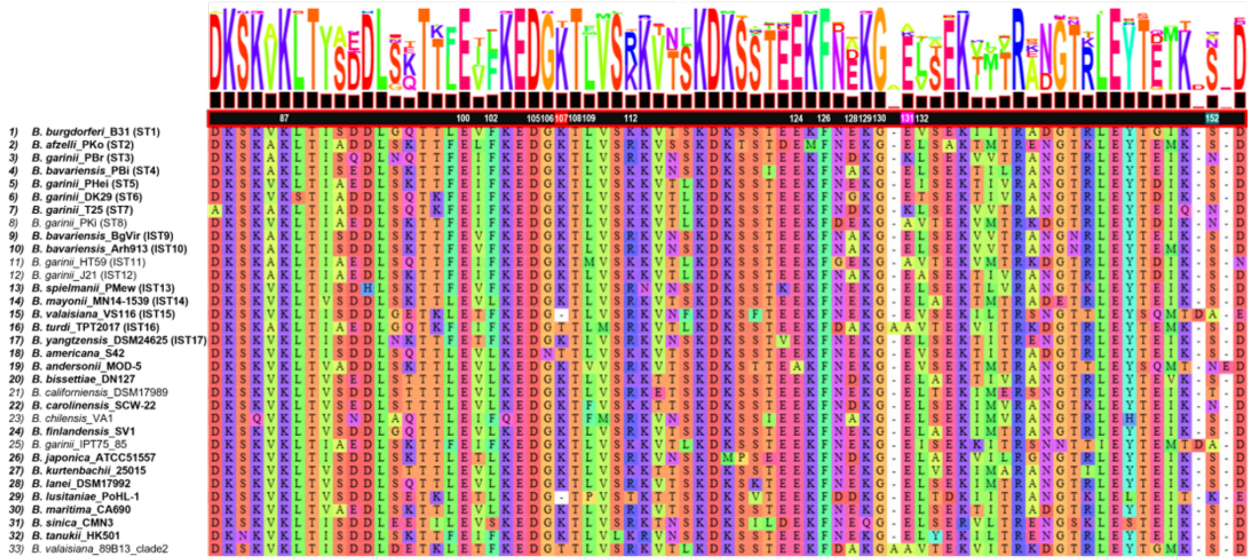

S7 Fig. Multiple sequence alignment depicting total sequence diversity within the Bin1 epitope region of 33 OspA types.
