# Supplementary material for: Broadly conserved protective epitopes on the lyme disease vaccine antigen, OspA
Source: PLoS Pathog. 2026 Apr 21;22(4):e1013740. doi: 10.1371/journal.ppat.1013740 (PMC13138739; doi:10.1371/journal.ppat.1013740)
Supplement: S8 Fig — (A) Antibody titration curves depicting complement-dependent killing of B. burgdorferi HB19-R1 reporter strains expressing OspA in silico types (ISTs). Complement-dependent killing assays were performed as described in the materials in methods section with anti-OspA Bin1 mAbs and HB19-R1 viability reporter strains expressing diverse OspA ISTs. The data shown encompasses at least three independent experiments per strain with data normalized as described. (B) Heat map summarizing statistical comparison of differences in Bin1 mAb susceptibility between the HB19-R1 OspAST1-expressing reporter strain and reporter strains expressing all other OspA variants as determined via one-way ANOVA followed by Dunnett’s multiple-comparison test. Significant differences in susceptibility relative to OspAST1 are denoted by purple or pink shading. (PDF) [file ppat.1013740.s014.pdf]

A.

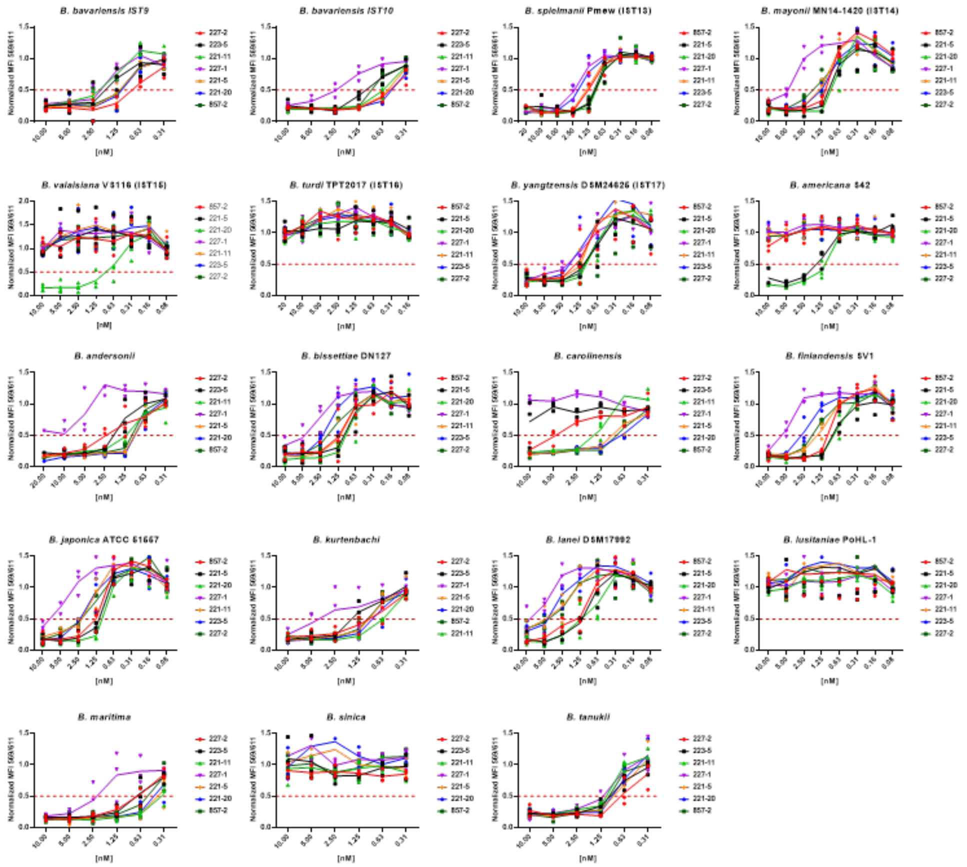

B.

	Class I			Class II			Class III
	857-2	221-5	221-20	221-11	227-2	223-5	227-1
Empty Vector (No OspA)	<0.0001	<0.0001	<0.0001	<0.0001	<0.0001	<0.0001	<0.0001
B. afzelli PKo (ST2)	0.9947	0.7903	>0.9999	>0.9999	>0.9999	0.1935	>0.9999
B. garinii PBr (ST3)	0.0771	>0.9999	0.2142	<0.0001	0.0014	0.0005	<0.0001
B. bavariensis PBi (ST4)	>0.9999	0.7903	>0.9999	>0.9999	>0.9999	0.9123	>0.9999
B. garinii PHei (ST5)	0.6380	0.8764	0.5866	>0.9999	0.9994	0.1935	0.2608
B. garinii DK29 (ST6)	0.9947	0.7903	0.9660	>0.9999	>0.9999	0.6571	>0.9999
B. garinii T25 (ST7)	0.0771	>0.9999	0.9660	0.2697	0.0014	0.3827	<0.0001
B. bavariensis BgVir (IST9)	0.0918	0.0025	0.8864	0.9963	0.2670	>0.9999	>0.9999
B. bavariensis FujiP2 (IST10)	>0.9999	>0.9999	0.9964	0.9963	0.9757	0.7255	>0.9999
B. speilmanii PMew (IST13)	0.3952	0.3006	>0.9999	0.9963	0.6675	>0.9999	>0.9999
B. mayonii MN14-1539 (IST14)	>0.9999	0.3006	0.8864	0.3730	0.6675	0.9136	0.8793
B. valaisiana VS116 (IST15)	<0.0001	<0.0001	0.0048	<0.0001	<0.0001	<0.0001	<0.0001
B. turdi TPT2017 (IST16)	<0.0001	<0.0001	<0.0001	<0.0001	<0.0001	<0.0001	<0.0001
B. yangtzensis DSM24625 (IST17)	0.8949	0.9180	0.3111	0.9995	0.9757	0.9914	>0.9999
B. americana S42	<0.0001	<0.0001	0.8864	<0.0001	<0.0001	<0.0001	<0.0001
B. andersonii MOD-5	>0.9999	0.3006	>0.9999	<0.0001	0.9757	>0.9999	0.0200
B. bissettiae DN127	0.8949	0.3006	>0.9999	0.3730	0.9757	>0.9999	0.2175
B. carolinensis SCW-22	>0.9999	0.3006	>0.9999	<0.0001	0.6675	<0.0001	>0.9999
B. finlandensis SV1	>0.9999	0.3006	0.3111	0.1123	0.6675	>0.9999	0.5164
B. japonica ATCC51557	0.3952	0.3006	>0.9999	0.0253	0.2670	>0.9999	0.5164
B. kurtenbachi 25015	0.9909	0.9180	>0.9999	0.2857	>0.9999	0.9914	>0.9999
B. lanei DSM17992	0.0918	0.0025	0.8864	0.0743	<0.0001	0.1721	<0.0001
B. lusitianiae PoHL-1	<0.0001	<0.0001	<0.0001	<0.0001	<0.0001	<0.0001	<0.0001
B. maritma CA690	>0.9999	0.9910	0.9697	0.9995	>0.9999	0.5003	>0.9999
B. sinica CMN3	<0.0001	<0.0001	<0.0001	<0.0001	<0.0001	<0.0001	<0.0001
B. tanukii HK501	>0.9999	0.3006	>0.9999	>0.9999	>0.9999	0.7255	>0.9999

Significance
<0.0001
<0.001
<0.01
<0.05
>0.05

S8 Fig. Genetically diverse OspA types are susceptible to complement-mediated killing by anti-OspA$_{ST1}$ Bin1 mAbs.
