# Supplementary material for: Broadly conserved protective epitopes on the lyme disease vaccine antigen, OspA
Source: PLoS Pathog. 2026 Apr 21;22(4):e1013740. doi: 10.1371/journal.ppat.1013740 (PMC13138739; doi:10.1371/journal.ppat.1013740)
Supplement: S11 Fig — Complement-dependent bactericidal assays were performed using HB19-R1 reporter strains and mAb 857-2 with either 2.5% guinea pig complement or 5% human complement (Pel-Freeze), under conditions described in the materials and methods section. Data shown encompasses 3–5 independent experiments. Differences in strain susceptibility to mAb 857-2 between complement sources were assessed across all strains by two-way ANOVA followed by Sidak’s multiple-comparisons test. No significant differences in susceptibility were observed. (PDF) [file ppat.1013740.s017.pdf]

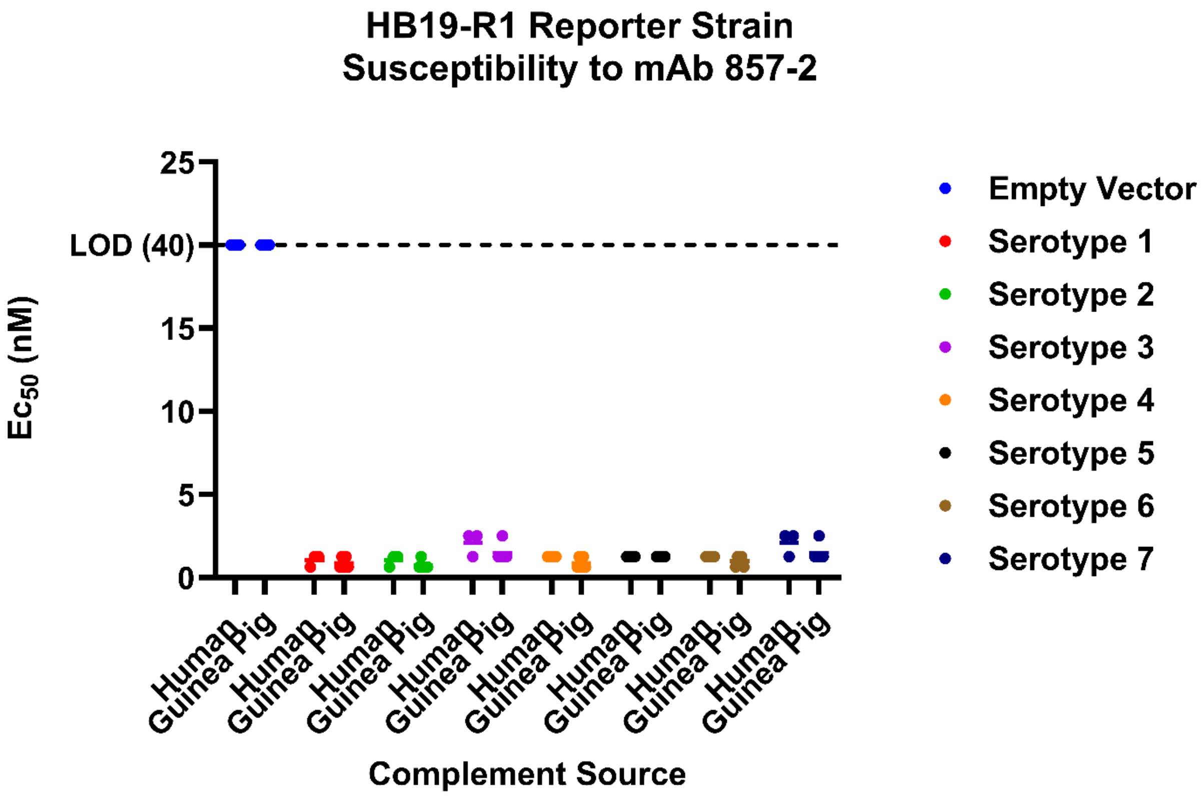

S11 Fig. Human and Guinea Pig-derived complement can be used interchangeably in SBAs with *B. burgdorferi* HB19-R1 reporter strains.
