# Supplementary material for: Broadly conserved protective epitopes on the lyme disease vaccine antigen, OspA
Source: PLoS Pathog. 2026 Apr 21;22(4):e1013740. doi: 10.1371/journal.ppat.1013740 (PMC13138739; doi:10.1371/journal.ppat.1013740)
Supplement: Attachment — Submitted filename: R2R_PP-D-25-02904_R2_Final.pdf [file ppat.1013740.s022.pdf]

Response to Reviewers PP-D-25-02904-R1

Reviewer #1: In response to this Reviewer's Comment 1.1 about whether the mabs recognize multiple epitopes, the authors stated 'The reviewer makes an excellent point and have revisited the text and corrected wording to make the point that there are likely multiple overlapping protective epitopes within this region of OspA.' However, the title of the revised manuscript 'A Broadly Conserved Protective Epitope on the Lyme Disease Vaccine Antigen, OspA' has not been amended to reflect this textual revision.

Response: We thank Reviewer 1 for the feedback. We have updated the title to reflect the comment. The title is now reads: “Broadly Conserved Protective Epitopes on the Lyme Disease Vaccine Antigen, OspA”
