# Supplementary material for: Broadly conserved protective epitopes on the lyme disease vaccine antigen, OspA
Source: PLoS Pathog. 2026 Apr 21;22(4):e1013740. doi: 10.1371/journal.ppat.1013740 (PMC13138739; doi:10.1371/journal.ppat.1013740)
Supplement: Attachment — Submitted filename: R2R_PP-D-25-02904_R1_Final.docx [file ppat.1013740.s021.docx]

**Response to Reviewers PP-D-25-02904-R1**

**Part I - Summary**
**Reviewer #1:** More than three decades after the demonstration that immunization with outer surface protein A (OspA) elicits protective antibodies, there is still no commercially available Lyme disease vaccine. Current OspA vaccine candidates undergoing clinical trials use polyvalent recombinant constructs fusing protective regions of OspAs derived from different serotypes. This meticulously executed, innovative, well-presented study uses a powerful combination of genetically engineered OspA reporter strains, borreliacidal human monoclonal antibodies, and structural analysis of OspA-Fab complexes to define a broadly conserved region of OspA’s central beta sheet that could become a universal Lyme disease vaccine. Some concerns noted elsewhere need the attention of the authors. Response: We thank Reviewer 1 for the feedback. Responses to specific concerns are noted below.

**Reviewer #2:** This manuscript presents a robust and comprehensive study identifying a broadly conserved protective epitope within the central β-sheet of OspA, a major antigenic target for Lyme disease vaccines. The authors integrate genetic engineering, functional immunology, and structural biology in an elegant manner. The creation of an isogenic panel of Borrelia burgdorferi reporter strains expressing OspA serotypes ST1–7 represents a methodological strength, enabling controlled and standardized comparison of cross-serotype antibody binding and complement-dependent borreliacidal activity. The structural analyses of Fab–OspA complexes are of high quality and provide strong mechanistic insight into epitope recognition.

This manuscript presents an ambitious and technically sophisticated study aiming to define a broadly conserved protective epitope within the central β-sheet of OspA, a major antigen for Lyme disease vaccine development. The integration of functional assays, structural biology, and engineered Borrelia reporter strains is a notable strength, and the identification of a potentially cross-serotype epitope is highly relevant to current efforts in next-generation OspA-based vaccine design.

Despite these strengths, several core conclusions rely heavily on engineered reporter strains and in vitro assays. The study would substantially benefit from data that demonstrate epitope accessibility and antibody activity in tick-relevant environments, as well as validation of the proposed susceptibility determinant Lys107 in native Borrelia isolates rather than only in a heterologous expression system. Additionally, variability in OspA expression across constructs has not been quantified, which raises concerns about whether functional differences reflect sequence-specific effects or expression levels.

The work is impactful and of high potential significance, but several experimental gaps currently limit the strength and generalizability of the conclusions. Addressing these issues is essential before acceptance. Response: We thank Reviewer 2 for the extensive review and critique. We have made every effort to respond to each of the comments below.

**Reviewer #3:** This is an excellent study showing that the central core of B. burgdorferi OspA (in the vicinity of B-sheets 6-10, aa82-146) is conserved across all major OspA serotypes. In addition, they identify the invariant aa residues that contribute to antibody recognition (Glu100, Asp105 and Lys107). The results are transformational for development of cross-reactive OspA based LD vaccines. Although the first OspA vaccines were based in B. burgdorferi B31 OspA (ST1), which is fully protective against all Bb strains transmitted by vector Ixodes in the United States (hence a hint of cross-reactive protection, and older evidence identifies OspA’ central B-sheet as cross-reactive), current OspA based LD vaccine iterations contain 7 serotypes to include the European Borreliella genospecies. Several companies are pursuing development of multivalent OspA vaccines, and the most advanced is Valneva/Pfizer recombinant protein which is comprised of 7 C-terminus fractions of OspAs ST1 to ST7 and is undergoing Phase III clinical trials. The study is very well designed, well executed, and the reporting of the results is rigorous. The scientific community working on OspA based vaccines for LD will be grateful for the set of B. burgdorferi spirochetes expressing each of the 7 OspA seroptypes as a tool to test vaccine efficacy in vitro by borreliacidal assay. Response: We thank Reviewer 3 for positive feedback on the scope and significance of the study.

**Part II – Major Issues: Key Experiments Required for Acceptance**

**Reviewer #1:** None needed.

**Reviewer #2:** While the manuscript is strong and presents compelling data, several essential experimental gaps must be addressed before the conclusions can be fully supported. These additional experiments are necessary for acceptance, as they directly impact the central claims of epitope accessibility, cross-serotype generalizability, and mechanistic interpretation.

**2.1. Functional confirmation of epitope accessibility in a tick-derived context. The study demonstrates broad mAb activity using in vitro reporter strains, but OspA expression and conformation differ substantially in the tick midgut. Since the proposed epitope is relevant for vaccine-mediated transmission blocking, the authors should provide at least one line of experimental evidence supporting epitope accessibility in a tick-relevant environment. Required experiment: Evaluate binding of one representative Class I mAb (e.g., 857-2 or 221-20) to Borrelia in dissected tick midguts or to in vitro tick cell–associated OspA, using flow cytometry or immunofluorescence. Rationale: Validates that the structurally defined epitope remains exposed during natural transmission.**

Response: We have revised the Introduction extensively to more explicitly address Reviewer 2’s concern. Mainly, we underscore that mAbs like 221-7 and 857-2 have been validated in both mouse and non-human primate models of tick-mediated *B. burgdorferi* challenge (see references therein and below). In fact, 221-17 has been advanced to Phase 1 clinical trials as a pre-exposure prophylaxis for Lyme disease (Lyme PrEP/TNX-4800). We have also underscored the fact that these and other mAbs used in the study have transmission blocking activities. We hope these key in vivo challenge studies address the Reviewer’s concerns:

Schiller ZA, Rudolph MJ, Toomey JR, Ejemel M, LaRochelle A, Davis SA, Lambert HS, Kern A, Tardo AC, Souders CA, Peterson E, Cannon RD, Ganesa C, Fazio F, Mantis NJ, Cavacini LA, Sullivan-Bolyai J, Hu LT, Embers ME, Klempner MS, Wang Y. Blocking Borrelia burgdorferi transmission from infected ticks to nonhuman primates with a human monoclonal antibody. J Clin Invest. 2021 Jun 1;131(11):e144843. doi: 10.1172/JCI144843. PMID: 33914704; PMCID: PMC8159683.

Wang Y, Kern A, Boatright NK, Schiller ZA, Sadowski A, Ejemel M, Souders CA, Reimann KA, Hu L, Thomas WD Jr, Klempner MS. Pre-exposure Prophylaxis With OspA-Specific Human Monoclonal Antibodies Protects Mice Against Tick Transmission of Lyme Disease Spirochetes. J Infect Dis. 2016 Jul 15;214(2):205-11. doi: 10.1093/infdis/jiw151. Epub 2016 Apr 18. PMID: 27338767; PMCID: PMC4918831.

**2.2 Validation of Lys107’s functional role in a native OspA background. The importance of Lys107 is well supported using engineered OspA variants in the HB19-R1 background. However, the manuscript extrapolates this to natural genospecies without direct functional validation. Required experiment: Complement-dependent killing assays using native isolates of at least one Lys107-deficient species (e.g., B. lusitaniae or B. turdi) and the corresponding engineered “Lys107-restored” mutant. Rationale: Demonstrates that Lys107 determines susceptibility in authentic clinical/genomic backgrounds, not only in the reporter system.**

Response: The reviewer raises a valid point. We would argue that the OspA derivates with the Lys107-deficient species were used in this study solely as as tools for the purpose of epitope mapping. In vivo validation of these strains is complicated in several respects and well beyond the scope of our current structure-function based efforts.

For example, the suggested experiment of using native isolates of at least one Lys107-deficient species (e.g., B. lusitaniae or B. turdi) is not feasible for because these strains are not readily available in the United State. Moreover, there is scant evidence to suggest that B. valaisiana, B. turdi, or B. lusitaniae are capable of causing disease in humans, and these genospecies therefore have minimal clinical relevance. As a result, these genospecies have remained poorly studied and culturable isolates are not readily available. Because these genospecies are only found in Europe and Asia, obtaining viable isolates for work in North America would be extremely difficult Finally, generating gene deletion strains in infectious genetic backgrounds of B. burgdorferi sensu stricto currently requires 2-3 months per strain. To our knowledge, all other B. burgdorferi sensu lato genospecies (including B. garinii and B. afzelii) are not genetically tractable.

**2.3 Statistical treatment of EC₅₀ differences across serotypes. The heatmaps and EC₅₀ values are compelling, but the manuscript lacks statistical comparison across serotypes and mAbs. Required modification of existing data: Provide statistical analysis (e.g., ANOVA or pairwise comparisons) for EC₅₀ differences across OspA serotypes within each mAb class. Rationale: Strengthens claims regarding potency hierarchy and cross-serotype differences.** Response: As requested, we have provided statistical analyses for results (EC50) shown in Figures 2, 7, and 8. Specifically, heat maps were generated that include corrected p-values for each strain/mAb, with significant differences in mAb susceptibility shown in purple or pink shading. The statistical heat maps corresponding Figures 2 and 7 are included in the Supplemental Information because their profiles track with EC50 values and duplicate display would be redundant.

However, in the case for Figure 8, statistical heat maps were added to this Figure as panel C because the analyses (two way ANOVA & Tukey’s multiple comparisons test) substantially improved the presentation of the data by graphically depicting significant changes in mAb susceptibility between reporter strains expressing WT and mutated OspA variants that were not obvious from the EC_50_ maps.

**Reviewer #3:** No major issues

**Part III – Minor Issues: Editorial and Data Presentation Modifications**
Please use this section for editorial suggestions as well as relatively minor modifications of existing data that would enhance clarity.

**Reviewer #1:**

**1.1. The authors vacillate between describing the mab binding region as an epitope or epitopes (e.g., line 197 "overlapping but distinct epitopes"). Per the title, the data give the general impression that the mAbs recognize one epitope with variable binding due to sporadic amino acid substitutions in OspA and Fab variable chains. To help clarify this issue and place the results in a broader context, it would be interesting to know about B cell epitope predictions for this central region of OspA and how they are affected by the amino acid substitutions that impact mAB reactivity.** Response: The reviewer makes an excellent point and have revisited the text and corrected wording to make the point that there are likely multiple overlapping protective epitopes within this region of OspA.

**1.2. Abstract, line 24. "Debilitating" is a bit excessive as a general descriptor for Lyme disease. The vast majority of patients with Lyme disease do not have debilitating manifestations.** Response: Point noted. We removed “debilitating”

**1.3. Introduction, line 60. Substitute "may result in" for "may involve".** Response: Text changed to “may involve.”

**1.4. Introduction, lines 92-93. Are the authors sure about this? The abstract to reference 17 states that protection was not observed when mice were immunized with a heterologous OspA.** Response: We have developed the following paragraph to clarify our interpretation of putative cross reactive epitopes on OspA.


**1.5. Results, line 124-125. This sentence could be worded better to more precisely state what about the strains is being validated.** Response: As requested, the text has been reworded to reflect validation of OspA expression on the surface of reporter strains.

**1.6. Results, line 135 and elsewhere. What does "Bin 1" mean? I have not been able to find a definition of the term.** Response: Apologies for the oversight. We have modified the Introduction to describe the different epitope bins on OspA.

**1.7. Results, lines 147-163. The data clearly indicate that OspA expression levels are the same in both strains but this should be clearly stated given the potential impact of this variable on the results.** Response: Noted & addressed by the addition of text stating that OspA expression is comparable between *B. burgdorferi* genetic backgrounds

**1.8. Figure 3. It would be helpful to indicate mAb class in the figure and label the reactive loops.** Response: As requested, we have modified Figure 3 to indicate which class the different mAbs correspond to.

**1.9. Figure 6B. It would be helpful if the authors could modify the figure to make the strains with the Lys 107 polymorphisms easier to spot.** Response: As requested the figure was modified to include stars next to the names of strains containing Lys 107 polymorphisms.

**1.10. Discussion, lines 374-375. Suggest adding this information to the appropriate place in the Results since it is an obvious question raised by the data presented.** Response: As requested, text was added to the Results section stating that attempts to solve the crystal structure of 221-20 FAbs in complex with OspA were not successful

**Reviewer #2:** 

**2.1 Some figure legends (particularly Figures 4–7) would benefit from additional detail and simplification.** Response: We thank the Reviewer for the recommendations. We have rewritten/revised Figure legends for clarity as requested.

**2.2 Supplementary figure captions (e.g., S7, S10) should better explain what the reader is expected to interpret.** Response: We have rewritten/revised Figure legends for clarity as requested.

**2.3 The choice of guinea pig complement should be justified more explicitly given known strain-specific complement sensitivities.** Response: In our hands, guinea pig complement and human complement are interchangeable and perform equivalently in the assay regarding sensitivity and reproducibility. However, the assay requires twice as much human complement (5%) as guinea pig complement (2.5%). Considering the cost of human complement we performed our experiments with guinea pig complement. The methods section was updated accordingly to reflect our rationale. We also provided an additional supplementary figure (S11) with data demonstrating that human and guinea pig complement can be used interchangeably in this assay.

**2.4 Please clarify whether recombinant OspA variants used in binding assays retain lipidation or are unlipidated forms.** Response:The recombinant OspA variants utilized in our experiments are non-lipidated. The text has been updated to reflect this.

**2.5 Structural PDB accession numbers should be explicitly referenced in the Data Availability Statement, not only in Methods.** Response: Updated, as requested.

**2.6 Figure 1: consider adding statistical information to binding differences.** Response: The statistical We thank the reviewer. We have updated the caption to read:

Compared to the empty (E controls, the gMFI values were statistically significantly greater for binding of LA-2 to ST1 (p<0.0001); 857-2 to ST1-7 (p<0.0001); 221-5 to ST1-7 (p<0.0001); 221-20 to ST1-7 (p<0.0001); 221-11 to ST1 (p<0.001), ST2, 4-7 (p<0.0001), and ST3 (p<0.05); 227-2 to ST1-2, 4-7 (p<0.0001) and ST3 (p<0.05); 227-1 to ST1-2, 4-6 (p≤0.0001). N=3. Statistical comparisons were performed using two-way ANOVA with Dunnet’s multiple-comparison test.

**2.7 Figure 2: adding a summary EC₅₀ table in the main text or supplement would improve readability.** Response: We have added a an supplemental table with statistical analysis to provide referecne for every mAb in tabular form.

**2.8 Figure 6: the phylogenetic tree is difficult to read at current scale; consider including zoomed panels.** Response: In response the reviewer’s comment we have increased the size of the graphic to improve readability. The images provide for online publication are of high enough resolution to enable zooming, as requested. We had difficult otherwise in including an isert.

**Reviewer #3:**

**3.1 Discussion: It may be useful to add a few words in the discussion on the residues that comprise B-sheets 6-10 because I found these buried in the methods section and it is an important fact to highlight vis a vis Valneva/Pfizer C-terminus vax currently in Phase III clinical trials. I am pretty confident these B-sheets fall outside the C-terminus of OspA for the most part. This will open-up discussion and fine-tuning of OspA vaccines, old and new. If I am wrong then that question is resolved with one statement.** Response: The review is indeed correct! We have modified the text to read as follows: “Within this region, there are invariant (e.g., Glu-100, Asp-105) and highly conserved residues responsible for cross-serotype recognition. Varying paratope interactions within and outside this core epitopes account for the different degrees of mAb reactivity across the seven major OspA serotypes. For example, single amino acid polymorphisms relative to OspA_ST1_ accounts for the reduced binding of 221-11 (**Class II**) and 227-1 (**Class III**) to OspA_ST3_ and OspA_ST7_. Nonetheless, these results suggest that it may be possible to design a “universal” monovalent Lyme disease vaccine antigen focused on OspA’s central β-sheet as an alternative strategy to multivalent chimeric vaccines derived from the variable C-terminal regions of the major OspA serotypes [23-26]. “

PLOS authors have the option to publish the peer review history of their article ([what does this mean?](https://gcc02.safelinks.protection.outlook.com/?url=https%3A%2F%2Ftrack.editorialmanager.com%2FCL0%2Fhttps%3A%252F%252Fjournals.plos.org%252Fplospathogens%252Fs%252Feditorial-and-peer-review-process%2523loc-peer-review-history%2F1%2F010f019b4943120e-40d52fcd-0b46-410a-9b8d-cdfb0ba19740-000000%2Fn_u6bbRZA9PDtat0aYJYjJ9ECB5DfQzLJAmnBQEgwm8%3D242&data=05%7C02%7Cdavid.vance%40health.ny.gov%7C39088a7d00bc427113e208de41d3c421%7Cf46cb8ea79004d108ceb80e8c1c81ee7%7C0%7C0%7C639020574954117450%7CUnknown%7CTWFpbGZsb3d8eyJFbXB0eU1hcGkiOnRydWUsIlYiOiIwLjAuMDAwMCIsIlAiOiJXaW4zMiIsIkFOIjoiTWFpbCIsIldUIjoyfQ%3D%3D%7C0%7C%7C%7C&sdata=h21n0B3pz8%2FkZh2gThUktl0sMBhKDCdyI%2BYgSMytcYM%3D&reserved=0)). If published, this will include your full peer review and any attached files.


If you choose “no”, your identity will remain anonymous but your review may still be made public.


**Do you want your identity to be public for this peer review?** For information about this choice, including consent withdrawal, please see our [Privacy Policy](https://gcc02.safelinks.protection.outlook.com/?url=https%3A%2F%2Ftrack.editorialmanager.com%2FCL0%2Fhttps%3A%252F%252Fwww.plos.org%252Fprivacy-policy%2F1%2F010f019b4943120e-40d52fcd-0b46-410a-9b8d-cdfb0ba19740-000000%2FdktrXMK4J6l4aNzKo69VP2iNlzV4mrKPqsvdOxtOSBc%3D242&data=05%7C02%7Cdavid.vance%40health.ny.gov%7C39088a7d00bc427113e208de41d3c421%7Cf46cb8ea79004d108ceb80e8c1c81ee7%7C0%7C0%7C639020574954146752%7CUnknown%7CTWFpbGZsb3d8eyJFbXB0eU1hcGkiOnRydWUsIlYiOiIwLjAuMDAwMCIsIlAiOiJXaW4zMiIsIkFOIjoiTWFpbCIsIldUIjoyfQ%3D%3D%7C0%7C%7C%7C&sdata=l6C2EhdoT16Blfmof8qpqToSsODd9OmQDdgGkbn1zPE%3D&reserved=0).

Reviewer #1: No

Reviewer #2: **Yes:** Dr CHOUMET Valérie

Reviewer #3: **Yes:** Maria Gomes-Solecki

**Figure resubmission:**

While revising your submission, we strongly recommend that you use PLOS’s NAAS tool (<https://ngplosjournals.pagemajik.ai/artanalysis>) to test your figure files. NAAS can convert your figure files to the TIFF file type and meet basic requirements (such as print size, resolution), or provide you with a report on issues that do not meet our requirements and that NAAS cannot fix.

After uploading your figures to PLOS’s NAAS tool - <https://ngplosjournals.pagemajik.ai/artanalysis>, NAAS will process the files provided and display the results in the "Uploaded Files" section of the page as the processing is complete. If the uploaded figures meet our requirements (or NAAS is able to fix the files to meet our requirements), the figure will be marked as "fixed" above. If NAAS is unable to fix the files, a red "failed" label will appear above. When NAAS has confirmed that the figure files meet our requirements, please download the file via the download option, and include these NAAS processed figure files when submitting your revised manuscript.

**Reproducibility:**

To enhance the reproducibility of your results, we recommend that authors of applicable studies deposit laboratory protocols in protocols.io, where a protocol can be assigned its own identifier (DOI) such that it can be cited independently in the future. Additionally, PLOS ONE offers an option to publish peer-reviewed clinical study protocols. Read more information on sharing protocols at <https://plos.org/protocols?utm_medium=editorial-email&utm_source=authorletters&utm_campaign=protocols>
